# Inferring the Direction of Introgression Using Genomic Sequence Data

**DOI:** 10.1093/molbev/msad178

**Published:** 2023-08-08

**Authors:** Yuttapong Thawornwattana, Jun Huang, Tomáš Flouri, James Mallet, Ziheng Yang

**Affiliations:** Department of Organismic and Evolutionary Biology, Harvard University, Cambridge, MA 02138, USA; School of Biomedical Engineering, Capital Medical University, Beijing 100069, P.R. China; Department of Genetics, Evolution and Environment, University College London, London WC1E 6BT, UK; Department of Organismic and Evolutionary Biology, Harvard University, Cambridge, MA 02138, USA; Department of Genetics, Evolution and Environment, University College London, London WC1E 6BT, UK; Department of Genetics, Evolution and Environment, University College London, London WC1E 6BT, UK

**Keywords:** Bpp, direction of gene flow, gene flow, introgression, multispecies coalescent

## Abstract

Genomic data are informative about the history of species divergence and interspecific gene flow, including the direction, timing, and strength of gene flow. However, gene flow in opposite directions generates similar patterns in multilocus sequence data, such as reduced sequence divergence between the hybridizing species. As a result, inference of the direction of gene flow is challenging. Here, we investigate the information about the direction of gene flow present in genomic sequence data using likelihood-based methods under the multispecies-coalescent-with-introgression model. We analyze the case of two species, and use simulation to examine cases with three or four species. We find that it is easier to infer gene flow from a small population to a large one than in the opposite direction, and easier to infer inflow (gene flow from outgroup species to an ingroup species) than outflow (gene flow from an ingroup species to an outgroup species). It is also easier to infer gene flow if there is a longer time of separate evolution between the initial divergence and subsequent introgression. When introgression is assumed to occur in the wrong direction, the time of introgression tends to be correctly estimated and the Bayesian test of gene flow is often significant, while estimates of introgression probability can be even greater than the true probability. We analyze genomic sequences from *Heliconius* butterflies to demonstrate that typical genomic datasets are informative about the direction of interspecific gene flow, as well as its timing and strength.

## Introduction

Gene flow between species occurs as a result of hybridization followed by backcrossing in one of the hybridizing species. While interspecific gene flow has a predominantly homogenizing effect, it may create new beneficial combinations of alleles at multiple loci, facilitating species diversification and adaptation (Arnold and Kunte 2017; Campbell et al. 2018; Feurtey and Stukenbrock 2018; Marques et al. 2019; Edelman and Mallet 2021). The outcome of introgression in each direction is influenced by multiple factors including mate choice (Peters et al. 2017), ecological selection, and hybrid incompatibility (for reviews, see Coyne and Orr 2004; Martin and Jiggins 2017; Moran et al. 2021). Given that these factors typically differ between species and that selection on introgressed material acts independently in different recipient species, it is likely that gene flow is often asymmetrical, being more prevalent in one direction than in the other. Reliable inference of the direction of introgression, as well as its timing and rate, will advance our understanding of this important evolutionary process and its consequences, including the role of gene flow during speciation and the adaptive nature of introgressed alleles.

Two models of interspecific gene flow have been developed in the multispecies coalescent (MSC) framework, representing different modes of gene flow (Jiao et al. 2021; Hibbins and Hahn 2022). The MSC-with-introgression (MSC-I; Flouri et al. 2020) model, also known as multispecies network coalescent (MSNC, Yu et al. 2012; Wen and Nakhleh 2018; Zhang et al. 2018), assumes that gene flow occurs at a particular time point in the past. The magnitude of gene flow is measured by the introgression probability (*φ*), the proportion of immigrants in the recipient population at the time of introgression. The MSC-with-migration (MSC-M) model, also known as the isolation-with-migration (IM) model, assumes that gene flow occurs continuously at a certain rate every generation after species divergence (Nielsen and Wakeley 2001; Hey et al. 2018). The rate of gene flow is measured by the expected number of immigrants from populations *A* to *B* per generation, $M_{AB}=N_{B}m_{AB}$, where $N_{B}$ is the (effective) population size of population *B* and $m_{AB}$ is the proportion of immigrants in population *B* from *A*. In both models, the rates of gene flow (*φ* or *M*) are “effective” rates, reflecting combined effects of gene flow and negative or positive natural selection on introgressed alleles, influenced by the local recombination rate (Petry 1983; Barton and Bengtsson 1986).

Interspecific gene flow alters gene genealogies, causing fluctuations over the genome in the genealogical history of sequences sampled from extant species. Under both the MSC-M and MSC-I models, gene trees and coalescent times have probabilistic distributions specified by the model and parameters, including species divergence times, population sizes for extant and extinct species, and the rate of gene flow (see Yang 2014; Jiao et al. 2021 for reviews). Multilocus sequence alignments are informative about gene tree topologies and coalescent times, and thus about the direction of gene flow as well as its timing and strength. However, opposite directions of gene flow often create similar features in gene genealogies and in the sequence data. For example, gene flow in either direction reduces the average and minimum divergence between the hybridizing species. In the special case of sampling one sequence per species per locus, the data cannot identify introgression direction between two sister species (say *A* and *B*), because the coalescent time ($t_{ab}$) between the two sequences at each locus (a,b) has the same distribution under the models with A→B or B→A introgression (Yang and Flouri 2022, fig. 10; see also Discussion). If multiple sequences are sampled per species per locus, introgression direction becomes identifiable (Yang and Flouri 2022). Even so, inference of introgression direction may be expected to be a challenging task. This is particularly so for heuristic methods for inferring gene flow based on summary statistics. For example, the *D* statistic (Green et al. 2010; Durand et al. 2011) operates on species quartets and cannot identify the direction of gene flow. Although heuristic methods exist for inferring the direction of gene flow, based on estimated local genomic divergences (Green et al. 2010, fig. S39) or genome-wide site-pattern counts $D_{\text{FOIL}}$ (Pease and Hahn 2015), they do not make efficient use of information in the data, often require a specific species phylogeny and sampling setup, and cannot infer gene flow between sister lineages. For recent discussions of the strengths and weaknesses of heuristic versus likelihood methods, see Jiao et al. (2021), Hibbins and Hahn (2022), Huang et al. (2022), and Yang and Flouri (2022).

Here, we study the inference of introgression direction, focusing on the Bayesian method under the MSC-I model (Flouri et al. 2020). Suppose introgression occurs from species A→B but we analyze genomic data assuming B→A introgression. We address the following questions. (a) Will we often detect introgression despite the assumed wrong direction? (b) How will the estimated introgression probability (${\hat{\varphi }}_{B\to A}$) compare with the true introgression probability ($\varphi_{A\to B}$)? (c) How reliable will estimates of the time of introgression be, as well as other parameters such as species divergence times and population sizes? (d) Does the method behavior differ depending on whether gene flow is between sister lineages or between nonsister lineages, and whether gene flow is from a small population to a large one, or in the opposite direction? (e) How can we infer the direction of introgression (A→B vs. B→A)? (f) Are typical genomic data informative about the direction of gene flow? We focus on both Bayesian estimation of parameters, in particular the introgression probability (Flouri et al. 2020), and on Bayesian tests of introgression (Ji et al. 2023).

We use a combination of mathematical analysis and computer simulation to characterize features of sequence data that are informative about the direction of gene flow. We first study the case of two species (A,B) by examining the distribution of coalescent times ($t_{aa},t_{ab},t_{bb}$) under the MSC-I model. The theory allows us to compare and quantify the amount of information in the data under different scenarios. Next, we explore the amount of information gained when a third species is added to branches of the species tree for two species and study the impact of introgression direction when gene flow involves nonsister species. Finally, we test these methods with genomic sequences from three species of *Heliconius* butterflies to verify the applicability of our results derived from the theoretical analysis and computer simulation and to demonstrate how the framework can be applied to infer the direction of gene flow, as well as its timing and strength. Our results provide practical guidelines for inferring introgression and its direction from genomic sequence data.

## Results

### Notation and Problem Setup

**Fig. 1.**
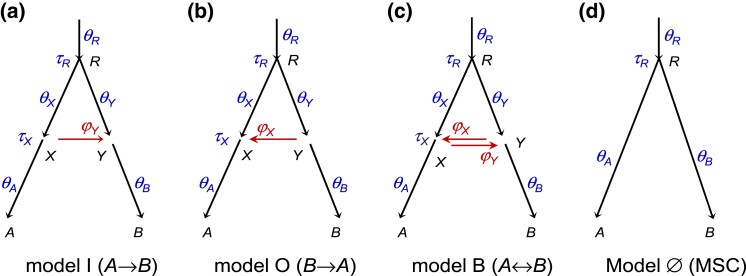
(*a*–*c*) MSC-I models for two species with different introgression directions showing model parameters: (*a*) A→B introgression (I for “inflow”) with $\Theta_{I}=(\tau_{R},\tau_{X},\theta_{A},\theta_{B},\theta_{X},\theta_{Y},\theta_{R},\varphi_{Y})$, (*b*) B→A introgression (O for “outflow”) with $\Theta_{O}=(\tau_{R},\tau_{X},\theta_{A},\theta_{B},\theta_{X},\theta_{Y},\theta_{R},\varphi_{X})$, or (*c*) bidirectional introgression (B) with $\Theta_{B}=(\tau_{R},\tau_{X},\theta_{A},\theta_{B},\theta_{X},\theta_{Y},\theta_{R},\varphi_{X},\varphi_{Y})$. The magnitude of introgression is measured by the introgression probability: $\varphi_{Y}\equiv \varphi_{A\to B}$ in *a* and *c* or $\varphi_{X}\equiv \varphi_{B\to A}$ in *b* and *c*. Note that in the MSC-I models studied in this paper, branches *RX* and *XA* represent distinct populations with different population size parameters ($\theta_{X},\theta_{A}$), as are branches *RY* and *YB*. Horizontal arrows (*XY* and *YX*) represent introgression events rather than real populations and have no *θ* associated with them. The arrow points to introgression direction in the real world (forward in time). (*d*) MSC model with no gene flow, with $\Theta_{⊘}=(\tau_{R},\theta_{A},\theta_{B},\theta_{R})$.

We use the MSC-I model of figure 1*a* with A→B introgression to introduce the notation and set up the problem. Species *A* and *B* diverged at time $\tau_{R}$ and hybridized later at time $\tau_{X}$. The magnitude of introgression is measured by the introgression probability or admixture proportion $\varphi_{Y}$, which is the proportion of immigrants in population *B* from *A* at the time of introgression. There are three types of parameters in the model: species divergence times or introgression times ($\tau_{R},\tau_{X}$), population sizes for extant and extinct species ($\theta_{A},\theta_{B},\theta_{X},\theta_{Y},\theta_{R}$), and the introgression probability ($\varphi_{Y}$). We measure divergence time (*τ*) by the expected number of mutations per site, with τ=Tμ, where *T* is the divergence time in generations and μ is the mutation rate per site per generation. As time *T* and rate *μ* are confounded in analysis of sequence data, only *τ* is estimable. Each branch on the species tree represents a species or population and is associated with a population size parameter, $\theta =4N_{e}\mu$, where $N_{e}$ is the (effective) population size of the species. A branch on the species tree is also referred to by its daughter node so that branch *RX* is also branch *X*, with population size $\theta_{X}$. Both *τ* and *θ* are measured as expected number of mutations per site; that is, one time unit is the expected time to accumulate one mutation per site. At this time scale, coalescence occurs between any two sequences in a population of size *θ* as a Poisson process with rate $\frac{2}{\theta }$.

Each dataset consists of sequence alignments at *L* loci, with $n_{A}$ sequences from *A* and $n_{B}$ sequences from *B* at each locus, and with *N* sites in each sequence. Underlying the sequences at each locus is a gene tree with branch lengths (coalescent times), with its probability distribution specified by the MSC-I model (Yu et al. 2014). We assume no recombination among sites in the sequence of the same locus and free recombination between loci; a recent simulation suggests that inference under the MSC is robust to moderate levels of recombination (Zhu et al. 2022). Under these assumptions, gene trees and sequence alignments are independent among loci. The data are analyzed under three MSC-I models that differ in introgression direction: model I with A→B introgression, model O with B→A introgression, and model B with bidirectional introgression (A⇆B) (fig. 1*a*–*c*). The “inflow” (I) and “outflow” (O) labels are used here in anticipation of models involving more than two species to be analyzed later. We use the multilocus sequence data to estimate parameters in the MSC-I model (Flouri et al. 2020). We also use the Bayesian test to detect the presence of gene flow, comparing an MSC-I model (fig. 1*a*–*c*) with the null model of MSC with no gene flow (fig. 1*d*) (Ji et al. 2023).

### The Case of Two Species

#### Distributions of Coalescent Times and Identifiability of Introgression Direction

We study the distributions of coalescent times between two sequences sampled from the same population ($t_{aa},t_{bb}$) or from different populations ($t_{ab}$). These are analytically tractable and are given in Appendix. Note that likelihood methods under the MSC-I model average over the full distribution of the gene tree (*G*) and coalescent times (t) for sampled sequences at every locus. However, this distribution depends on the number of sequences sampled per species ($n_{A},n_{B}$) and is too complex to analyze. Instead, we examine the pairwise coalescent times ($t_{aa},t_{ab},t_{bb}$) as important summaries of the data, and use their distributions to demonstrate the identifiability of introgression direction, to characterize the information content in estimation of introgression probability, and to predict the behavior of Bayesian parameter estimation (Flouri et al. 2020) and Bayesian test of gene flow (Ji et al. 2023). Note that our theory for coalescent times applies to arbitrary sample configurations ($n_{A},n_{B}$); for example, if multiple sequences are sampled per species, $t_{ab}$ will refer to any pair of sequences, one from *A* another from *B*.

First, we ask whether introgression direction can be inferred using sequence data sampled from extant species. From equation (A2), we have $f_{I}(t_{ab})=f_{O}(t_{ab})$ for all $t_{ab}>0$, with the parameter mapping $\tau_{R}^{\left(O\right)}=\tau_{R}^{\left(I\right)}$, $\tau_{X}^{\left(O\right)}=\tau_{X}^{\left(I\right)}$, $\theta_{Y}^{\left(O\right)}=\theta_{X}^{\left(I\right)}$, $\theta_{R}^{\left(O\right)}=\theta_{R}^{\left(I\right)}$, and $\varphi_{X}=\varphi_{Y}$, where the superscripts indicate the assumed model. Thus, $t_{ab}$ alone cannot distinguish models I and O. In other words, in the case of two species, introgression direction is unidentifiable using data of only one sequence per species per locus (Yang and Flouri 2022, fig. 10; see also Discussion).

**Fig. 2.**
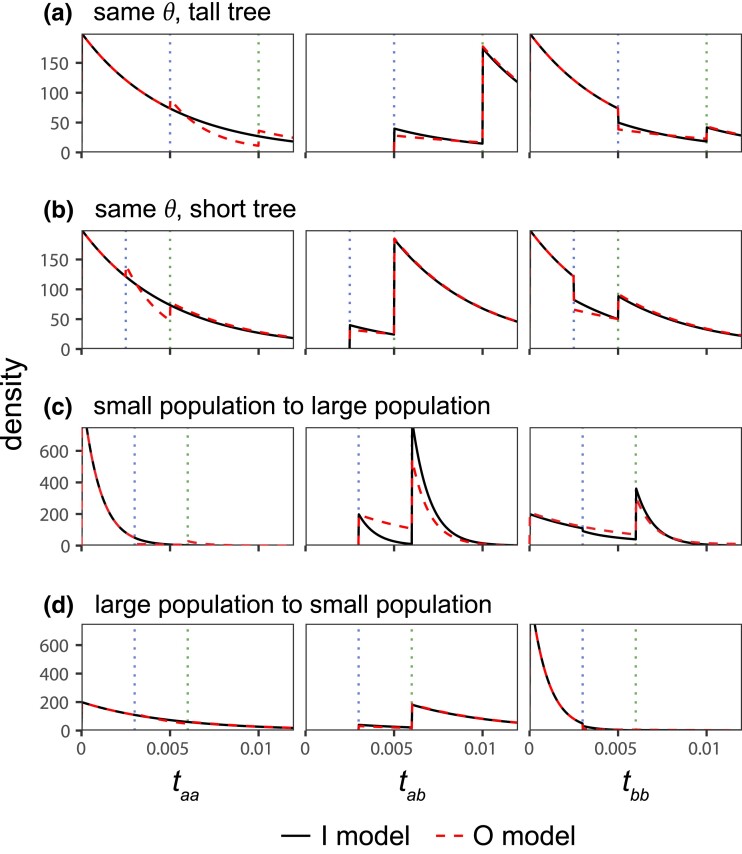
The true (solid line for model I) and fitted (dashed line for model O) distributions of coalescent times ($t_{aa},t_{ab},t_{bb}$) for four sets of parameter values (cases **a**–**d**; panels [a]-[d]). Data are generated under model I and analyzed under model O of figure 1*a* and *b*. Densities for model I are calculated using the true parameter values ($\Theta_{I}$ in supplementary table S1, Supplementary Material online); see equations (A1)–(A3), while those for model O are calculated using the best-fitting parameter values, approximated by average estimates in Bpp analysis of simulated large datasets (with L=4,000 loci, n=4 sequences per species per locus and N=500 sites in the sequence) ($\Theta_{O}^{*}$ in supplementary table S1, Supplementary Material online). Vertical dotted lines indicate discontinuity points at $\tau_{X}$ and $\tau_{R}$.

However, introgression direction is identifiable if multiple sequences are sampled from *A* and *B*. Information for distinguishing models I and O comes mostly from coalescent times between sequences sampled from the same species ($t_{aa},t_{bb}$). If gene flow is A→B, the coalescent time for sequences from the donor species, $t_{aa}$, is not affected by the A→B introgression. If different populations on the species tree have the same size ($\theta_{A}=\theta_{X}=\theta_{R}$), $t_{aa}$ will have a smooth exponential distribution (e.g., fig. 2*a*, model I). Otherwise the distribution is discontinuous at time points $\tau_{X}$ and $\tau_{R}$, because of population size changes. In contrast, $t_{bb}$ has a mixture distribution, depending on the hybridizing species to which each of the two *B* sequences is traced back on the gene genealogy (i.e., either parental species *RX* or *RY* at node *Y*, fig. 1*a*). Thus, the two models make different predictions about coalescent times $t_{aa}$ and $t_{bb}$, and the direction of introgression is identifiable when multiple sequences are sampled per species per locus.

If the introgression direction is specified (i.e., under the unidirectional introgression model), introgression probability (e.g., $\varphi_{Y}$ given model I) is identifiable using data of one sequence per species per locus. However, the bidirectional introgression model (model B) involves an *unidentifiability of the label-switching type*, with two unidentifiable modes or “towers” in the posterior surface if multiple sequences are sampled per species (Yang and Flouri 2022), or four unidentifiable modes if a single sequence is sampled per species; see Discussion for details.

#### Asymptotic Analysis and Best-Fitting Parameter Values

We consider multilocus datasets generated under model I with A→B introgression (fig. 1*a*) and analyzed under both model I and the misspecified model O with B→A introgression. We used four sets of parameter values in model I (fig. 1*a*) in the numerical calculation, referred to as cases **a**–**d** (fig. 2, supplementary table S1, Supplementary Material online). When the amount of data (the number of loci) L→∞, the maximum likelihood estimates (MLEs) under model I (${\hat{\Theta }}_{I}$) will converge to the true parameter values, that is, ${\hat{\Theta }}_{I}\to \Theta_{I}$. Under model O, the MLEs ${\hat{\Theta }}_{O}$ will converge to the *best-fitting* or *pseudo-true* parameter values ($\Theta_{O}^{*}$), which minimize the Kullback–Leibler (KL) divergence from the true model to the fitting model: ${\hat{\Theta }}_{O}\to \Theta_{O}^{*}$ (e.g., Yang and Zhu 2018). With arbitrary data configurations, it does not seem possible to calculate $\Theta_{O}^{*}$ analytically. Instead, we use as a substitute the averages of posterior means of parameters in Bpp analysis of simulated large datasets (with L=4,000 loci, $n_{A}=n_{B}=4$ sequences per species per locus and N=500 sites per sequence), shown in supplementary table S1, Supplementary Material online. At this data size, average estimates under the true model I are extremely close to the true values, that is, $E({\hat{\Theta }}_{I})\approx \Theta_{I}$ (supplementary table S1, Supplementary Material online), suggesting that the average estimate under model O may also be very close to the infinite-data limits, $E({\hat{\Theta }}_{O})\approx \Theta_{O}^{*}$. We aim to understand the estimates $\Theta_{O}^{*}$ by comparing the true distributions of coalescent times under model I, $f_{I}(t_{aa}),f_{I}(t_{ab})$, and $f_{I}(t_{bb})$ (eqs. A1–A3), with fitted distributions $f_{O}(t_{aa}),f_{O}(t_{ab})$, and $f_{O}(t_{bb})$, calculated using $\Theta_{O}^{*}$. In other words, we treat the true distributions of coalescent times under model I as data, and attempt to derive parameter estimates under the fitting model O to achieve the best fit.

Our theory is summarized in table 1. Note that parameters $\tau_{R},\tau_{X},\theta_{A},\theta_{B},\theta_{R}$ in model O are typically well estimated. Introgression time $\tau_{X}^{\left(O\right)}$ is largely determined by the smallest coalescent time between sequences from the two species ($t_{ab}$), while the discontinuity in the distributions of $t_{aa},t_{ab},t_{bb}$ should be informative about $\tau_{R}^{\left(O\right)}$. Thus, we expect estimates of those parameters to be close to the true values despite the model misspecification: $\tau_{R}^{*\left(O\right)}\approx \tau_{R}^{\left(I\right)}$ and $\tau_{X}^{*\left(O\right)}\approx \tau_{X}^{\left(I\right)}$. Population sizes $\theta_{A}^{\left(O\right)}$ and $\theta_{B}^{\left(O\right)}$ for the extant species should be well estimated from multiple samples from the same species, while $\theta_{R}^{\left(O\right)}$ should be well estimated based on coalescent events in the root population. Below we focus on parameters $\theta_{X}^{\left(O\right)},\theta_{Y}^{\left(O\right)}$, and $\varphi_{X}$, which are harder to estimate.

**Table 1.** Features of the Data that are Informative About Parameters in the Wrong Model O When Data are Generated Under Model I with Parameter $\Theta^{\left(I\right)}$ (fig. 1*a*).

	Parameter Estimates in Model O	Information in Data	Notes
(a)	Introgression time: ${\hat{\tau }}_{X}^{\left(O\right)}\approx \tau_{X}^{\left(I\right)}$	$\min\{t_{ab}\}$	In the fitting model O, $t_{ab}>\tau_{X}^{\left(O\right)}$. Thus, introgression time $\tau_{X}^{\left(O\right)}$ is determined by the minimum between-species coalescent time ($t_{ab}$)
(b)	Species divergence time: ${\hat{\tau }}_{R}^{\left(O\right)}\approx \tau_{R}^{\left(I\right)}$	Discontinuities in $f(t_{aa})$, $f(t_{ab})$, and $f(t_{bb})$	Species divergence time is informed by the discontinuities in the coalescent times ($t_{aa},t_{ab},t_{bb}$)
(c)	Population sizes for extant species: ${\hat{\theta }}_{A}^{\left(O\right)}\approx \theta_{A}^{\left(I\right)}$, ${\hat{\theta }}_{B}^{\left(O\right)}\approx \theta_{B}^{\left(I\right)}$	$f(t_{aa})$ and $f(t_{bb})$ over ($0,\tau_{X}$)	Population size for an extant species is easily estimated by the heterozygosity in the species
(d)	Population sizes for ancestral species not involved in introgression: ${\hat{\theta }}_{R}^{\left(O\right)}\approx \theta_{R}^{\left(I\right)}$	$f(t_{aa})$ , $f(t_{ab})$, and $f(t_{bb})$ over ($\tau_{X},\infty$)	Population sizes for ancestral species not involved in introgression are determined by coalescent times in the ancestral species
(e)	Ancestral population size: ${\hat{\theta }}_{X}^{\left(O\right)}<\theta_{X}^{\left(I\right)}$	$f(t_{aa})$ over $\left(\tau_{X},\tau_{R}\right)$	The fitting model O predicts a deficit of coalescence of *A* sequences ($t_{aa}$) over $\left(\tau_{X},\tau_{R}\right)$ due to introgression but there is no such deficit in the true model I or in the data. Having a larger coalescent rate (or smaller population size $\theta_{X}^{\left(O\right)}$) in model O thus helps to improve the model fit to coalescence of *A* sequences in the data
(f)	Ancestral population size: ${\hat{\theta }}_{Y}^{\left(O\right)}>\theta_{Y}^{\left(I\right)}$	$f(t_{bb})$ over $\left(\tau_{X},\tau_{R}\right)$	There is a deficit of coalescence of *B* sequences ($t_{bb}$) over $\left(\tau_{X},\tau_{R}\right)$ in the true model I or in the data. Having a smaller coalescent rate (or larger population size $\theta_{Y}^{\left(O\right)}$) in model O thus helps with the model fit
(g)	Introgression probability: ${\hat{\varphi }}_{X}(1-e^{-2\Delta \tau /{\hat{\theta }}_{Y}^{\left(O\right)}})\approx \varphi_{Y}(1-e^{-2\Delta \tau /\theta_{X}^{\left(I\right)}})$ (eq. 1): ${\hat{\varphi }}_{X}>\varphi_{Y}$ if ${\hat{\theta }}_{Y}^{\left(O\right)}>\theta_{X}^{\left(I\right)}$, ${\hat{\varphi }}_{X}<\varphi_{Y}$ if ${\hat{\theta }}_{Y}^{\left(O\right)}<\theta_{X}^{\left(I\right)}$.	$f(t_{ab})$ over $\left(\tau_{X},\tau_{R}\right)$	Introgression probability is informed by the amount of between-species coalescence ($t_{ab}$) over $\left(\tau_{X},\tau_{R}\right)$. Equation (1) means the same amount of coalescence in species *Y* in the fitting model as in species *X* in the true model

Note.—The introgression model assumes different population sizes (θs) for species on the tree (fig. 1); the behavior of the method may differ if all populations are assumed to have the same size. Also the reasoning here is based on coalescent times and ignores sampling errors in gene trees and estimated coalescent times in the analysis of sequence data.

First, by considering the distributions of $t_{aa}$, we predict $\theta_{X}^{*\left(O\right)}<\theta_{X}^{\left(I\right)}$ (table 1). In the true model I, both *A* sequences enter *X* and may coalesce during $\left(\tau_{X},\tau_{R}\right)$. In the fitting model O, the two *A* sequences may be separated into different populations due to introgression (one in *X* and the other in *Y*), so they may not coalesce in $\left(\tau_{X},\tau_{R}\right)$ as often. Thus, having $\theta_{X}^{*\left(O\right)}<\theta_{X}^{\left(I\right)}$ will increase the coalescent rate in *X* and help to fit model O to $f(t_{aa})$ over $\left(\tau_{X},\tau_{R}\right)$.

Next from $t_{bb}$, we predict $\theta_{Y}^{*\left(O\right)}>\theta_{Y}^{\left(I\right)}$ (table 1). In the true model, A→B introgression reduces the chance of coalescence between sequences from *B* during $\left(\tau_{X},\tau_{R}\right)$. In the fitting model, both *B* sequences enter *Y*, leading to a higher chance of coalescence during $\left(\tau_{X},\tau_{R}\right)$. Thus, having $\theta_{Y}^{*\left(O\right)}>\theta_{Y}^{\left(I\right)}$ helps to reduce the chance of coalescence in $\left(\tau_{X},\tau_{R}\right)$.

Finally, by matching the amount of coalescence between sequences *a* and *b* over the time interval ($\tau_{X},\tau_{R}$), or by matching the probability densities $f_{I}(t_{ab})$ and $f_{O}(t_{ab})$ over $\tau_{X}<t_{ab}<\tau_{R}$, we have approximately


(1) $$\varphi_{X}^{*}(1-e^{-2\Delta \tau /\theta_{Y}^{*\left(O\right)}})\approx \varphi_{Y}(1-e^{-2\Delta \tau /\theta_{X}^{\left(I\right)}}),$$

where $\Delta \tau =\tau_{R}-\tau_{X}$ is assumed to be the same under models I and O based on the arguments above. Equation (1) predicts that more gene flow will be inferred under model O ($\varphi_{X}^{*}>\varphi_{Y}$) when $\theta_{Y}^{*\left(O\right)}>\theta_{X}^{\left(I\right)}$; if the coalescent rate between sequences *a* and *b* during ($\tau_{X},\tau_{R}$) is lower in the fitting model than in the true model, a higher $\varphi_{X}^{*}$ than the true $\varphi_{Y}$ will increase the chance of such coalescence and achieve a better fit to $f_{I}(t_{ab})$. Similarly, less gene flow is expected (with $\varphi_{X}^{*}<\varphi_{Y}$) if $\theta_{Y}^{*\left(O\right)}<\theta_{X}^{\left(I\right)}$.

Equation (1) predicts $\varphi_{X}^{*}$ to be 0.31, 0.35, 0.44, and 0.22 for cases **a**–**d**, respectively, compared with the inferred values $\varphi_{X}^{*}$ of 0.27, 0.30, 0.98, and 0.17 (supplementary table S1, Supplementary Material online). The approximation is reasonably good except for case *c*, where $\varphi_{X}^{*}$ was very high. We discuss these cases further when describing simulation results below.

#### Simulation Results Under the True Models I and B: Parameter Estimates Have Drastically Different Precisions

**Fig. 3.**
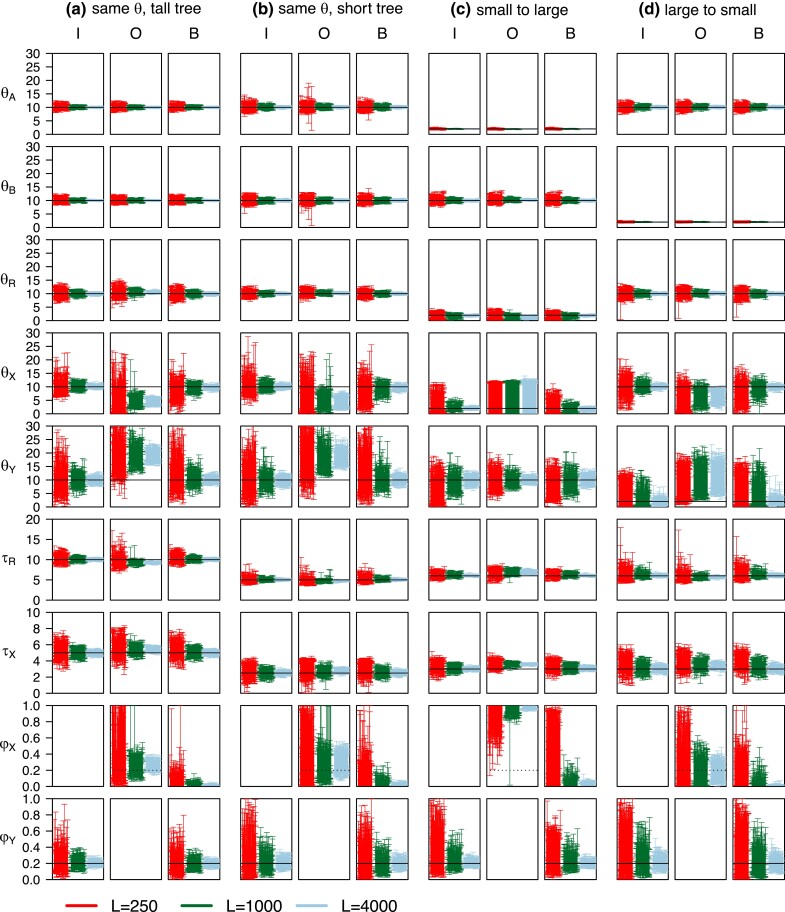
The 95% HPD CIs for parameters in 100 replicate datasets (each of *L* loci) simulated under model I and analyzed under models I, O, and B of figure 1*a*–*c*. Four sets of parameter values are used (cases **a**–**d; panels [a]-[d]**) (supplementary table S1, Supplementary Material online). Parameters *θ*s and *τ*s are multiplied by $10^{3}$. Black solid lines indicate the true values. Dotted lines for $\varphi_{X}$ in model O indicate the true value of $\varphi_{Y}$ in model I.

To verify and extend our theoretical analysis, we simulated datasets under model I (fig. 1*a*) and analyzed them under models I, O, and B (fig. 1*a*–*c*) using four sets of parameter values. Each dataset consists of L=250,1,000 or 4,000 loci, with $n_{A}=n_{B}=4$ sequences sampled per species per locus and N=500 sites in the sequence. Posterior means and 95% highest-probability-density (HPD) credibility intervals (CIs) are plotted in figure 3 (see also supplementary table S1, Supplementary Material online for L=4,000).

Model I is the true model, so that the performance under this model constitutes the best-case scenario. Indeed all parameters are well estimated, with the posterior means approaching true values and the CI width approaching 0 when the amount of data L→∞ (fig. 3 and supplementary table S1, Supplementary Material online, cases **a**-**d**, model I). However, the amount of information in the data varies hugely for different parameters, as reflected in the relative error, measured, for example, by the CI width divided by the true value. Population sizes for extant species ($\theta_{A},\theta_{B}$) are much better estimated than those for ancestral species ($\theta_{X},\theta_{Y}$). Divergence times ($\tau_{R},\tau_{X}$) are well estimated as well. Introgression probability ($\varphi_{Y}$) has substantial uncertainties with wide CIs but with L=4,000 loci in the data, the estimates are fairly precise, suggesting that thousands of loci are necessary to estimate introgression probability precisely. The results parallel those found in a previous simulation examining the impact of data size (such as the number of loci, the number sequences per species, and the number of sites) on inference under the MSC-I model (Huang et al. 2020).

Model B allows bidirectional introgression and thus is a correct model, although it is overparametrized with an extra parameter $\varphi_{X}$. As the amount of data increases, ${\hat{\varphi }}_{Y}$ should converge to the true value while ${\hat{\varphi }}_{X}$ to 0. Estimates of other parameters are very similar to those under model I, and the CI widths under models I and B are also very similar. In particular, $\varphi_{Y}$ is estimated with similar precision in the two models. In large datasets of L=4,000 loci, the average CI width is 0.07, 0.12, 0.08, and 0.16 for cases **a**–**d** under model I, compared with 0.07, 0.12, 0.09, 0.17 under model B. Even in small or intermediate datasets with L=250 or 1,000 loci, the CIs for $\varphi_{Y}$ are similar between the two models. Thus, overparametrization incurred little cost to statistical performance of model B. This might seem surprising, because, given the difficulty of inferring introgression direction, one might expect the assumed incorrect B→A introgression in model B would interfere with estimation of $\varphi_{Y}$ in the correct direction, so that ${\hat{\varphi }}_{Y}$ would have a much larger variance under model B than under model I. However, information concerning $\varphi_{Y}$ is largely determined by 1) the number of sequences reaching the hybridization node *Y* and 2) the ease with which one can tell the parental path taken by each *B* sequence at *Y* (see the next subsection for detailed discussions). Thus, there may be little difference in information content about $\varphi_{Y}$ between models I and B. Computationally, model B is much more expensive than model I due to sampling an extra parameter in the Markov chain Monte Carlo (MCMC) algorithm and to MCMC mixing issues (Yang and Flouri 2022).

#### Information Content for Estimating Introgression Probability Under the True Model

Here, we consider estimation of introgression probability $\varphi_{Y}$ in model I in the four cases (fig. 3, cases **a**–**d**, model I). We characterize the amount of information concerning $\varphi_{Y}$ when the correct model is assumed, and explain why $\varphi_{Y}$ was much better estimated in case **a** (same *θ* tall tree) than in case **b** (same *θ* short tree), and in case **c** (small to large) than in case **d** (large to small) (fig. 3; supplementary table S1, Supplementary Material online: cases **a**–**d**, model I), even though the data size is the same and the true $\varphi_{Y}$ is the same (0.2) in all cases. The theory is also useful for understanding later simulation results for larger species trees.

Consider tracing the genealogical history of sequences at a locus backwards in time. When sequences from *B* reach the hybridization node *Y* (fig. 1*a*), there is a binomial sampling process, with each sequence taking the horizontal (introgression) parental path (into *RX*) with probability $\varphi_{Y}$ and the vertical parental path (into *RY*) with $1-\varphi_{Y}$. However, there are two differences from a typical binomial sampling. First, the number of *B* sequences reaching node *Y* is a random variable. Second, the outcome of the sampling process (i.e., the parental path taken by the sequence) is not observed but instead reflected in the gene tree and coalescent times (and thus in mutations in the sequences). Using a coin-tossing analogy, the number of coin tosses is random, and the outcome of the toss is visible only probabilistically. If a *B* sequence coalesces with an *A* sequence during the time interval ($\tau_{X},\tau_{R}$), it will be clear that the *B* sequence has taken the introgression parental path.

Thus, the amount of information in the data concerning $\varphi_{Y}$ is determined by two factors: 1) the number of *B* sequences reaching *Y* and 2) the ease with which one can tell the parental path taken by each *B* sequence at *Y*. The number of *B* sequences reaching *Y* at the locus is given as $n_{B}-c_{B}$, where $n_{B}$ is the number of *B* sequences sampled at the locus and $c_{B}$ is the number of coalescent events among them in *B* before reaching *Y*. The distribution of $n_{B}-c_{B}$ can be easily calculated as a function of $n_{B}$ and $2\tau_{Y}/\theta_{B}$, the length of branch *B* measured in coalescent units (Tavaré 1984: eqs. 6.1 and 6.2; Wakeley 2009: eqs. 3.39 and 3.41). More *B* sequences will reach *Y* the larger $n_{B}$ is and the smaller $2\tau_{Y}/\theta_{B}$ is. As a result, it will be harder to estimate $\varphi_{Y}$ if introgression is older (larger $\tau_{Y}$).

The second factor—the ease with which one can tell the parental path taken by each *B* sequence at *Y*—concerns the probability that two sequences entering *X* coalesce in *X* before reaching *R*; there is more information about $\varphi_{Y}$ the longer the internal branch *RX* is or the smaller the population size $\theta_{X}$ is (fig. 1*a*). This may be seen by considering the special case where the data consist of one sequence per species per locus and where the true coalescent time ($t_{ab}$) is available at each locus. Then the information content for estimating $\varphi_{Y}$ may be measured by the Fisher information, given by


(2) $$I_{I,t_{ab}}(\varphi_{Y})\approx E\left[-\frac{\partial^{2}}{\partial \varphi_{Y}^{2}}\log\;f_{I}(t_{ab})\right]=\frac{P_{X}}{\varphi_{Y}(1-\varphi_{Y}P_{X})},$$

where the expectation is with respect to $t_{ab}$ (eq. A3), and where $P_{X}=1-e^{-(2/\theta_{X})(\tau_{R}-\tau_{X})}$ is the probability that two sequences (a,b) entering population *X* coalesce in *X*. The asymptotic variance of the estimate (${\hat{\varphi }}_{Y}$) is


(3) $$V({\hat{\varphi }}_{Y})\approx \frac{1}{IL}=\frac{\varphi_{Y}(1-\varphi_{Y}P_{X})}{LP_{X}}\geq \frac{\varphi_{Y}(1-\varphi_{Y})}{L},$$

with equality holding if $P_{X}=1$. There is thus more information for estimating $\varphi_{Y}$ the closer $P_{X}$ is to 1, or in other words if the branch length in coalescent units, $\left(2/\theta_{X})(\tau_{R}-\tau_{X}\right)$, is greater. Increasing the number of sequences reaching *Y* per locus ($n_{B}-c_{B}$) may be expected to have a similar effect to increasing the number of loci (*L*) as both increases the binomial sample size. Equation (3) thus suggests that increasing $P_{X}$ is more effective in reducing $V({\hat{\varphi }}_{Y})$ than increasing the number of loci (*L*) by the same factor, which is in turn more effective than increasing the number of sampled sequences per locus ($n_{B}$) by the same factor. For example, doubling $n_{B}-c_{B}$ reduces the variance for ${\hat{\varphi }}_{Y}$ by a half, but doubling $P_{X}$ reduces the variance by more than a half.

In our simulation (fig. 3, model I), the introgression probability $\varphi_{Y}$ was better estimated in case **a** (same *θ* tall tree) than in case **b** (same *θ* short tree). At L=4,000, the 95% HPD CI width was 0.07 for case **a**, and 0.12 for case **b**. Consider the two factors. First, in case *a* (tall tree), branch *YB* is longer, with length $2\tau_{Y}/\theta_{B}$ in coalescent units, with a smaller number of sequences reaching *Y* than in case **b** (short tree). Indeed, given $n_{B}=4$ sequences from *B*, the probability that $n_{B}-c_{B}=$ 1, 2, 3, and 4 sequences remain by time $\tau_{Y}$ is 0.388, 0.515, 0.095, and 0.002, respectively in case **a**, with an average of 1.71 (supplementary fig. S1, Supplementary Material online). For the short tree of case **b**, the corresponding probabilities are 0.122, 0.481, 0.347, and 0.050, with average 2.32. The average number of sequences reaching *Y* differ by a factor 1.36. Second, in case **a** (tall tree), any *B* sequence reaching *Y* and taking the left parental path is more likely to coalesce with *A* sequences in *X* than in case **b** (short tree), with $P_{X}=1-e^{-1}=0.632$ in case **a** and $P_{X}=1-e^{-0.5}=0.393$ in case **b**, differing by a factor of 1.61. As increasing $P_{X}$ is more effective than increasing $n_{B}-c_{B}$ (eq. 3), $\varphi_{Y}$ was more precisely estimated (with smaller variance) in case **a** than in **b** (fig. 3; supplementary table S1, Supplementary Material online).

The difference between case **c** (small to large) and case **d** (large to small) was even greater, with $\varphi_{Y}$ much better estimated in **c** (fig. 3). At L=4,000, the CI width was 0.08 for case **c** and 0.16 for case **d** (supplementary table S1, Supplementary Material online). In case **c**, more *B* sequences reach *Y* because of the large $\theta_{B}$ than in case **d**. Furthermore, *B* sequences reaching *Y* into *X* have a high chance of coalescence with other sequences in population *X*. Both effects make it easier to estimate $\varphi_{Y}$ in case **c** than in case **d** (eq. 3). It is thus easier to estimate $\varphi_{Y}$ if introgression is from a small population to a large one than in the opposite direction (supplementary fig. S2, Supplementary Material online). Note that $\varphi_{Y}$ is the proportion of immigrants in the recipient population, so that with the same $\varphi_{Y}$, there are many more migrants in case **c** than in **d**.

#### Parameter Estimation Under Misspecified Introgression Direction

When model O was used to analyze data simulated under model I (fig. 1), the introgression direction is misspecified. As discussed above (table 1), species divergence and introgression times ($\tau_{R},\tau_{X}$) are well estimated despite misspecification, as are population sizes for extant species and for the root ($\theta_{A},\theta_{B},\theta_{R}$). Indeed, those parameters are estimated with the same precision under models O and I (fig. 3).

Here, we focus on parameters $\varphi_{X}$, $\theta_{X}$, $\theta_{Y}$ (fig. 3, model O). Our arguments from the asymptotic analysis (table 1) also apply, although in simulations the results are affected by random sampling errors due to finite data size.

In cases **a** and **b**, all populations have the same size. Biases in parameter estimates under model O are well predicted by the theory (table 1): based on coalescent times $t_{aa},t_{bb}$, and $t_{ab}$, we expect $E({\hat{\theta }}_{X}^{\left(O\right)})<\theta_{X}^{\left(I\right)}$, $E({\hat{\theta }}_{Y}^{\left(O\right)})>\theta_{Y}^{\left(I\right)}$, and $E({\hat{\varphi }}_{X})>\varphi_{Y}$.

In case **c** (small to large), introgression is from a small population to a large one. As the coalescent rate for sequences *a* and *b* over ($\tau_{X},\tau_{R}$) is much slower in the fitting model than in the true model, consideration of $t_{ab}$ predicts a large ${\hat{\varphi }}_{X}$ or a small ${\hat{\theta }}_{Y}^{\left(O\right)}$ (table 1). Consideration of $t_{bb}$ suggests ${\hat{\theta }}_{Y}^{\left(O\right)}>\theta_{Y}^{\left(I\right)}$ will compensate for reduced coalescence between *B* sequences caused by the A→B introgression (table 1). Thus, predictions about ${\hat{\theta }}_{Y}^{\left(O\right)}$ based on $t_{ab}$ and $t_{bb}$ are somewhat conflicting. In the simulation, ${\hat{\theta }}_{Y}^{\left(O\right)}$ is close to $\theta_{Y}^{\left(I\right)}$, much larger than $\theta_{X}^{\left(I\right)}$. The estimate is ${\hat{\varphi }}_{X}\approx 100\%$ (supplementary table S1, Supplementary Material online). The extreme estimate causes small biases in $\tau_{R}$ and $\tau_{X}$ and poor estimates of ${\hat{\theta }}_{X}^{\left(O\right)}$ (fig. 3).

Case **d** (large to small) assumes introgression from a large population to a small one (fig. 1*a*). We expect ${\hat{\theta }}_{X}^{\left(O\right)}<\theta_{X}^{\left(I\right)}$ based on $t_{aa}$, and ${\hat{\theta }}_{Y}^{\left(O\right)}>\theta_{Y}^{\left(I\right)}$ based on $t_{bb}$ (table 1). Moreover, the larger source population in the true model ($\theta_{X}^{\left(I\right)}$) means $t_{ab}$ is less common in $\left(\tau_{X},\tau_{R}\right)$, with most coalescence occurring in the common ancestor *R*. Thus, based on $t_{ab}$ we predict a larger ${\hat{\theta }}_{Y}^{\left(O\right)}$ or a smaller ${\hat{\varphi }}_{X}$ to reduce the amount of coalescence in $\left(\tau_{X},\tau_{R}\right)$ in the fitting model (eq. 1). Thus, considerations of both $t_{bb}$ and $t_{ab}$ suggest ${\hat{\theta }}_{Y}^{\left(O\right)}>\theta_{Y}^{\left(I\right)}$. Depending on whether ${\hat{\theta }}_{Y}^{\left(O\right)}$ is smaller or greater than $\theta_{X}^{\left(I\right)}$, the introgression probability ${\hat{\varphi }}_{X}$ may be greater or smaller than the true $\varphi_{Y}$, according to equation (1). In our setting, ${\hat{\theta }}_{Y}^{\left(O\right)}=0.0107$, slightly greater than $\theta_{X}^{\left(I\right)}=0.01$, and ${\hat{\varphi }}_{X}=0.17$, slightly smaller than $\varphi_{Y}=0.2$ (supplementary table S1, Supplementary Material online).

#### Bayesian Test of Introgression: Power and False Positive Rate

We applied the Bayesian test of gene flow (Ji et al. 2023) to the data analyzed in figure 3. We are interested in the power of the test under the correct model I. Also we ask how often the test is significant if it is conducted under model O, with introgression direction misspecified.

Note that the behavior of the test or the asymptotic behavior of posterior probabilities of the compared models is determined by the parameter values in the limit of L→∞ (Yang and Zhu 2018). If data are simulated under model I (with $\varphi_{Y}>0$) and analyzed under model I, the posterior probability for the true model I should approach 1, the Bayes factor in support of model I against model Ø of no gene flow (fig. 1*d*) $B_{I⊘}\to \infty$, and the power of the test should approach 100%, when the data size L→∞ (Yang and Zhu 2018). If the data are simulated under model I and analyzed under model B, the power for testing $\varphi_{Y}$ (which has the true value $\varphi_{Y}>0$) should approach 100%, and the false positive rate for testing $\varphi_{X}$ (which has the true value $\varphi_{X}=0$) should approach 0, when the data size L→∞.

If the data are generated under model I and analyzed under model O, both the null and alternative models are incorrect. According to our analysis $\varphi_{X}^{*}>0$, and model O is a “less wrong” model than model Ø, judged by the KL divergence (Yang and Zhu 2018). Thus, when L→∞, $B_{\text{O}Ø}\to \infty$, and the probability of rejecting $H_{⊘}\;:\;\varphi_{X}=0$ will approach 100%. Here, the biological interpretation of test results is somewhat ambiguous. If one emphasizes the fact that model O allows gene flow while model Ø does not, detecting gene flow may be considered a correct result. However, if one emphasizes misspecification of introgression direction in model O, accepting model O may be considered a rather severe false positive error. In this paper, we use the second interpretation.

The MCMC samples generated in Bpp runs of figure 3 were processed to calculate the Bayes factor $B_{10}$ in favor of the introgression model ($H_{1}$, fig. 1*a*–*c*) against the null MSC model of no gene flow ($H_{0}$, fig. 1*d*) via the Savage–Dickey density ratio (see Materials and Methods). The results are summarized in supplementary figure S2, Supplementary Material online, where a 1% significance level was used (i.e., the test is significant if $B_{10}>100$). When the data were simulated and analyzed under model I and with L=250 loci in the data, power was between 60–100% (supplementary fig. S2, Supplementary Material online, cases **a**-**d**, model I). In such small datasets, $\varphi_{Y}$ was poorly estimated with extremely wide CIs (fig. 3, cases **a**–**d**, model I). At L=1,000 loci, power was 100% in all four cases. It is thus easier to detect gene flow than to estimate its magnitude reliably. As with our findings on estimation of $\varphi_{Y}$, it is easier to detect gene flow in case **a** (tall tree) than in case **b** (short tree), and in case **c** (small → large) than in case **d** (large → small) (supplementary fig. S2, Supplementary Material online).

When the data are analyzed under model O, with the introgression direction misspecified, the false positive error is comparable to the power in the analysis under true model I (supplementary fig. S2, Supplementary Material online, cases **a**–**d**, model O). When the data are analyzed under model B, power to detect the A→B introgression is slightly lower than under model I, also reaching 100% at L=1,000, while the false positive rate for detecting the nonexistent B→A introgression is low, below the nominal 1%.

### Additional Information that Results from Including a Third Species

**Fig. 4.**
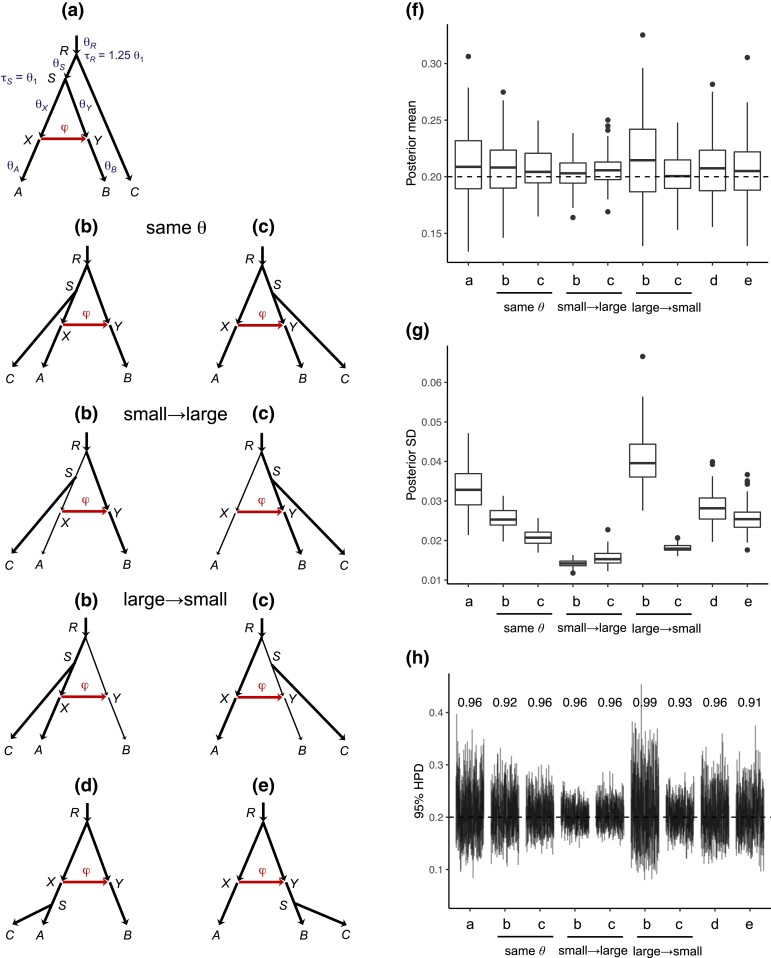
(*a*–*e*) MSC-I models for three species (A,B,C), with introgression from *A* to *B*, obtained by adding a third species *C* onto the two-species tree of figure 1*a* at five possible locations: (*a*) root population, (*b*,*c*) source and target populations before gene flow, and (*d*,*e*) source and target populations after gene flow. (*f*) Box plots of the posterior means for *φ* among 100 replicate datasets simulated under each of the five cases (**a**–**e**). The dashed line indicates the true value (φ=0.2). (*g*) Box plots of the posterior SD for *φ*. (*h*) 95% HPD CIs for *φ*, with the CI coverage above the CI bars. See supplementary figure S3, Supplementary Material online for CIs for other parameters.

Given two species (A,B) with introgression from A→B at the rate of *φ* (fig. 1*a*), we consider the information gain for estimating *φ* from including a third species (*C*). There are five branches on the two-species tree onto which *C* can be attached (fig. 4*a*–*e*): (*a*) the root population, (*b*,*c*) the source and target populations before gene flow, and (*d*,*e*) the source and target populations after gene flow. Case **c** is one of “inflow,” with gene flow from the outgroup species (*A*) into one of the ingroup species (*B*), while **b** represents “outflow,” with gene flow from an ingroup species (*A*) into the outgroup (*B*). Note that in all cases the correct MSC-I model is used in the analysis, so that the estimate (posterior mean) of *φ* will converge to the true value (which is 0.2). However, the information content may differ among the five cases. As in the case of two species, the amount of information concerning *φ* is determined by two factors: 1) the number of sequences reaching the hybridization node and 2) the ease with which one can tell the parental path taken by each sequence at the hybridization node. When introgression is between nonsister species, information concerning the parental path taken by each sequence may be in the change of gene-tree topology rather than in the change of between-species coalescent time.

We assumed the same population size $\theta_{1}=0.01$ for all populations, but examined the impact of different population sizes in cases **b** and **c**. We simulated 100 replicate datesets in each case. The posterior means, the posterior standard deviation (SD), and the width of the HPD CI for *φ* are summarized in figure 4*f*–*h*. The 95% CIs for other parameters are shown in supplementary figure S3, Supplementary Material online.

#### Equal Population Sizes on the Species Tree

If all populations on the species tree have the same size (*θ*), we expect the amount of information for estimating *φ* to be in the order **a**≺**d**≺ (**b**, **e**) ≺**c**, with the order of **b** and **e** undecided (fig. 4*f*–*h*).

First, **a**≺**d**. Cases **a** and **d** are the least informative. Adding an outgroup species *C* in case **a** adds little information about *φ*. In **d**, the *C* sequences may reach node *X* and coalesce with a *B* sequence in *RX*, providing information about whether sequences from *B* take the introgression parental path at node *Y*. Thus, we expect more information in the data in **d** than in **a**.

Next, **d**≺**b**. The number of *B* sequences reaching node *Y* is the same in the two cases, so the only difference is in the difficulty of inferring the parental path taken by *B* sequences at *Y*. In case **b**, coalescence of a *B* sequence with an *A* sequence causes a change to gene tree topology. In case **d**, introgression does not cause such topological change to the gene tree. The information content may thus be higher in **b** than in **d**.

Next, **d**≺**e**. In case **e**, sequences from both *B* and *C* may reach the hybridization node *Y* while in **d** only sequences from *B* may reach *Y*, so that the sample size at node *Y* is larger (less than twice as large) in **e** than in **d**. In **d**, more sequences enter population *RX*, increasing slightly the probability of coalescence for any *B* sequence that takes the introgression parental path at *Y*, but this effect may be less important than that of increased sample size in **e**.

Next, **b**≺**c** (i.e., it is easier to infer inflow than outflow). In both cases, the number of *B* sequences reaching node *Y* or the sample size at *Y* is the same. However, the two cases differ in the ease with which one can tell the parental path taken by each *B* sequence at *Y*. In **c**, coalescence of a *B* sequence with an *A* sequence over $\left(\tau_{X},\tau_{R}\right)$ causes a change to gene tree topology. In case **b**, such topology change occurs only if the coalescence occurs in the shorter time interval $\left(\tau_{X},\tau_{S}\right)$, and the resulting gene tree is harder to infer because of the shorter internal branch. It is thus harder to resolve the parental path taken by each *B* sequence at *Y* in **b** than in **c**, and the data are less informative about *φ* in **b**. It is harder to infer outflow than inflow.

Finally, **e**≺**c**. In case **c**, introgression leads to changes in gene tree topology whereas in **e**, more sequences reach *Y* with a larger sample size. The relative effects depend on the parameter values. In the simulation here, the increased sample size was less effective than the gene tree topology change (fig. 4*g* and *h*, case **c** same-*θ* vs. case **e**). Note that in **e** the data are more informative about *φ* the closer $\tau_{S}$ is to $\tau_{Y}$, and in both **c** and **e** the data are more informative the smaller $\tau_{X}$ is.

#### Different Population Sizes on the Species Tree

For cases **b** (outflow) and **c** (inflow), we also consider different population sizes. The results are shown in figure 4*f*–*h*.

First, in case **b**, *φ* is most poorly estimated in the large→small setting, much better estimated in the same-*θ* (or large→large) setting, and best in the small→large setting. This can be explained easily by the theory we developed in analysis of the two species case: a large recipient population means many sequences reaching the hybridization node *Y* and a large sample size, while a small donor species ($\theta_{X}$) means fast coalescence and easy determination of the parental path taken at node *Y*. For example, the probability that more than one *B* sequence reaches *Y* is 0.613 in case **b** (same *θ* or small→large), and 0.012 in case **b** (large→small), with a large difference in the sample size.

Similarly in case **c** (inflow), *φ* is more poorly estimated in the large→small and same-*θ* (large→large) settings, and was better in the small→large setting. The differences among the three settings are much smaller than in case **b**.

Although case **b** outflow is less informative about *φ* than **c** inflow in the case of same-*θ*, the order is reversed in the small→large setting (fig. 4). The same number of *B* sequences reaches node *Y* in both cases, so the difference must be due to the different levels of difficulty by which one can tell the parental paths taken by *B* sequences at node *Y*. In case **b**, *B* sequences taking the introgression parental path go through the small population *SX* and may coalesce at a high rate with sequences from *A* (which lead to changes to the gene tree topology informative about introgression), and with sequences from both *A* and *C* in population *RS*. In case **c**, *B* sequences taking the vertical parental path may coalesce in population *RS* with *C* sequences, but given that both populations *SY* and *RS* are large, this effect may be expected to be minor. While multiple factors can have opposing effects on the relative information content concerning *φ* in cases **b** versus **c** small→large, the data are more informative in case **b** than in **c** overall.

### Simulation Results in the Case of Four Species

**Fig. 5.**
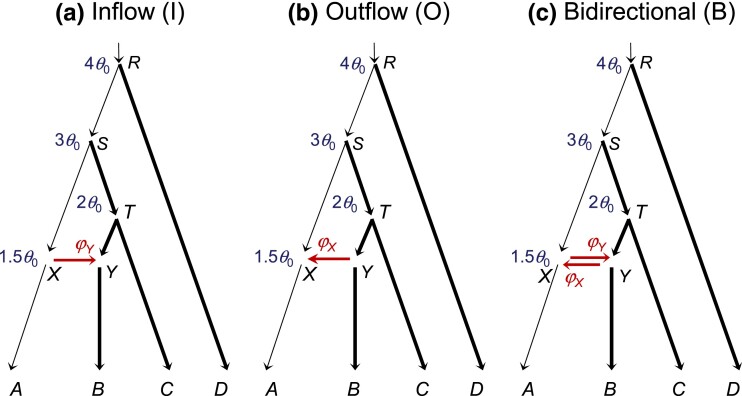
(4s-trees) Three MSC-I models for four species differing in introgression direction assumed to simulate and analyze data: (*a*) inflow from *A* to *B* (I); (*b*) outflow from *B* to *A* (O); and (*c*) bidirectional introgression between *A* and *B* (B). Divergence times used are shown next to the nodes: $\tau_{R}=4\theta_{0}$, $\tau_{S}=3\theta_{0}$, $\tau_{T}=2\theta_{0}$, and $\tau_{X}=\tau_{Y}=1.5\theta_{0}$, with population sizes $\theta_{0}=0.002$ for the thin branches and $\theta_{1}=0.01$ for the thick branches. We also used a setting in which all populations on the species tree have the same size, with $\theta_{0}=\theta_{1}=0.01$. Introgression probabilities are $\varphi_{X}=\varphi_{Y}=0.2$. Data simulated under models I, O, and B are analyzed under models I, O, and B, resulting in nine combinations, with parameter estimates summarized in supplementary figure S4, Supplementary Material online (for the same population size) and supplementary figure S5, Supplementary Material online (for different population sizes), while results of the Bayesian test are presented in supplementary figure S6, Supplementary Material online (for the same population size) and supplementary figure S7, Supplementary Material online (for different population sizes).

We conducted simulations under the MSC-I models of figure 5 for four species on the species tree ((A,(B,C)),D), with introgression between nonsister species *A* and *B* in different directions: inflow (I), outflow (O), and bidirectional introgression (B). Either the same population size was assumed for all species on the species tree or different population sizes were assumed. The simulated data were analyzed under the same three models (I, O, B), resulting in nine combinations. Posterior means and 95% HPD CIs are summarized in supplementary figure S4, Supplementary Material online for the case of equal population sizes and in supplementary figure S5, Supplementary Material online for different population sizes. The results for the large datasets of L=4,000 are summarized in supplementary tables S2 and S3, Supplementary Material online. We also applied the Bayesian test of introgression (Ji et al. 2023) to the simulated data. The results are summarized in supplementary figures S6 and S7, Supplementary Material online.

Overall, the results parallel those for the cases of two and three species discussed above. See the Supplementary material online text “Simulation results in the case of four species” for detailed descriptions.

### Analysis of *Heliconius* Genomic Datasets to Infer the Direction of Introgression

#### Overview

**Fig. 6.**
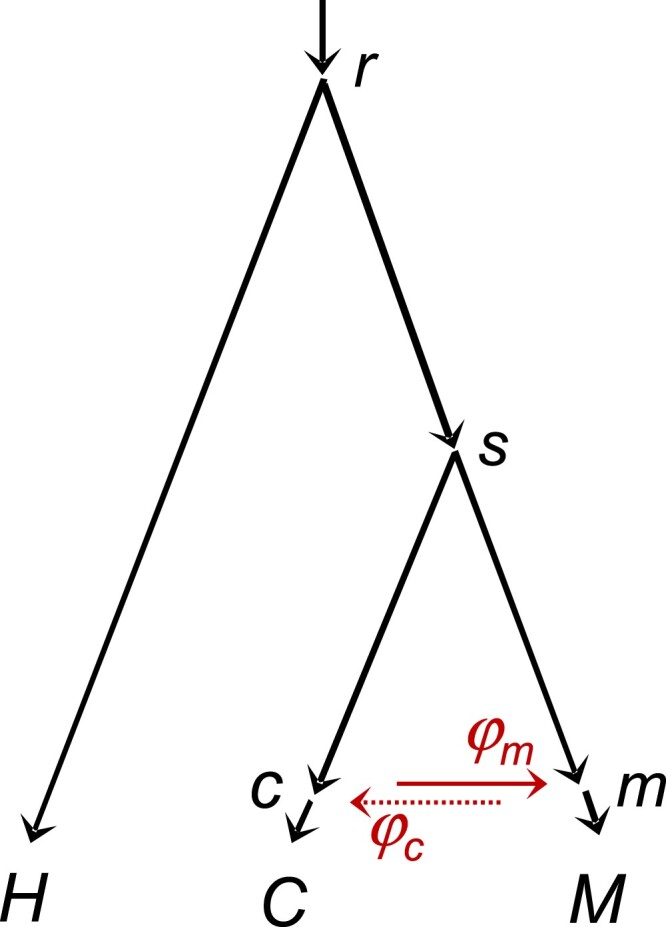
Species tree for *Heliconius hecale* (*H*), *H. cydno* (*C*), and *H. melpomene* (*M*), with introgression between *H. cydno* and *H. melpomene*, used to analyze genomic sequence data. Parameters in the MSC-I model include species divergence and introgression times ($\tau_{r},\tau_{s},\tau_{c}=\tau_{m}$), population sizes for branches on the species tree (e.g., $\theta_{C}$ for branch *C* and $\theta_{c}$ for branch *sc*), as well as introgression probabilities ($\varphi_{m}\equiv \varphi_{C\to M}$ and $\varphi_{c}\equiv \varphi_{M\to C}$). The data support the C→M introgression but not the M→C introgression, with $\varphi_{m}>0$ and $\varphi_{c}\approx 0$ (table 2; supplementary table S4 and fig. S8, Supplementary Material online).

To assess the applicability of our results from the asymptotic analysis and computer simulation to empirical datasets and the statistical and computational feasibility of inferring the direction of gene flow using genomic sequence data, we analyzed data from *Heliconius cydno* (*C*), *H. melpomene* (*M*), and *H. hecale* (*H*) (fig. 6). Gene flow is known to occur between *H. cydno* and *H. melpomene*, whereas *H. hecale* is more distantly related, and is here treated as an outgroup, and is assumed not to have had introgression with the other two (Martin et al. 2013). We analyzed coding and noncoding loci on each chromosome as separate datasets (see supplementary table S4, Supplementary Material online for the numbers of loci). We fitted four models: (Ø) MSC with no gene flow, (I) MSC-I with C→M introgression, (O) MSC-I with M→C introgression, and (B) MSC-I with C⇆M bidirectional introgression (see fig. 1). We ran the MCMC algorithm in Bpp to generate the posterior estimates of parameters in each model (Flouri et al. 2020) and conducted the Bayesian test of introgression (Ji et al. 2023). We describe the results for the coding and noncoding datasets from chromosome 1 (tables 2 and 3) in detail before discussing results for the other chromosomes.

**Table 2.** Posterior Means and 95% HPD CIs for Parameters in Bpp Analyses of Two Datasets of Noncoding and Coding Loci on Chromosome 1 from *Heliconius* Butterflies (fig. 6) Under Four Models with Different Introgression Directions.

	Model Ø (no gene flow)	Model I (C→M)	Model O (M→C)	Model B (C⇆M)
*Noncoding loci* (L=5,341 loci)				
$\theta_{H}$	0.0131 (0.0127, 0.0136)	0.0134 (0.0129, 0.0139)	0.0134 (0.0129, 0.0138)	0.0134 (0.0129, 0.0139)
$\theta_{C}$	0.0407 (0.0329, 0.0496)	0.0500 (0.0274, 0.0759)	0.0231 (0.0070, 0.0415)	0.0499 (0.0267, 0.0759)
$\theta_{M}$	0.0026 (0.0021, 0.0031)	0.0003 (0.0002, 0.0005)	0.0001 (0.0000, 0.0002)	0.0003 (0.0002, 0.0005)
$\theta_{r}$	0.0124 (0.0119, 0.0128)	0.0123 (0.0118, 0.0127)	0.0122 (0.0118, 0.0127)	0.0123 (0.0118, 0.0127)
$\theta_{s}$	0.0343 (0.0328, 0.0358)	0.0152 (0.0141, 0.0162)	0.0185 (0.0175, 0.0194)	0.0152 (0.0141, 0.0162)
$\theta_{c}$	n/a	0.0256 (0.0241, 0.0271)	0.0230 (0.0206, 0.0254)	0.0255 (0.0240, 0.0270)
$\theta_{m}$	n/a	0.0188 (0.0162, 0.0214)	0.0294 (0.0262, 0.0327)	0.0189 (0.0164, 0.0215)
$\tau_{r}$	0.0116 (0.0114, 0.0117)	0.0118 (0.0116, 0.0120)	0.0118 (0.0116, 0.0120)	0.0118 (0.0116, 0.0120)
$\tau_{s}$	0.0010 (0.0008, 0.0012)	0.0068 (0.0064, 0.0072)	0.0051 (0.0048, 0.0053)	0.0068 (0.0064, 0.0071)
$\tau_{c}=\tau_{m}$	n/a	0.0001 (0.0001, 0.0002)	0.0000 (0.0000, 0.0001)	0.0001 (0.0001, 0.0002)
$\varphi_{c}$	n/a	n/a	0.1744 (0.1458, 0.2038)	0.0019 (0.0000, 0.0057)
$\varphi_{m}$	n/a	0.2830 (0.2565, 0.3090)	n/a	0.2802 (0.2530, 0.3067)
*Coding loci* (L=4,942 loci)				
$\theta_{H}$	0.0055 (0.0053, 0.0058)	0.0055 (0.0053, 0.0058)	0.0055 (0.0052, 0.0057)	0.0055 (0.0053, 0.0058)
$\theta_{C}$	0.0054 (0.0048, 0.0060)	0.0361 (0.0203, 0.0545)	0.0307 (0.0133, 0.0513)	0.0363 (0.0204, 0.0553)
$\theta_{M}$	0.0016 (0.0015, 0.0018)	0.0010 (0.0008, 0.0011)	0.0005 (0.0003, 0.0008)	0.0010 (0.0008, 0.0011)
$\theta_{r}$	0.0092 (0.0088, 0.0096)	0.0092 (0.0088, 0.0096)	0.0094 (0.0090, 0.0098)	0.0092 (0.0088, 0.0096)
$\theta_{s}$	0.0117 (0.0111, 0.0124)	0.0027 (0.0004, 0.0054)	0.0092 (0.0084, 0.0100)	0.0027 (0.0004, 0.0053)
$\theta_{c}$	n/a	0.0059 (0.0055, 0.0063)	0.0044 (0.0032, 0.0055)	0.0058 (0.0053, 0.0062)
$\theta_{m}$	n/a	0.0119 (0.0076, 0.0168)	0.0105 (0.0072, 0.0144)	0.0129 (0.0077, 0.0189)
$\tau_{r}$	0.0049 (0.0047, 0.0050)	0.0049 (0.0047, 0.0050)	0.0048 (0.0047, 0.0050)	0.0049 (0.0047, 0.0050)
$\tau_{s}$	0.0009 (0.0008, 0.0010)	0.0047 (0.0045, 0.0049)	0.0017 (0.0015, 0.0019)	0.0047 (0.0045, 0.0049)
$\tau_{c}=\tau_{m}$	n/a	0.0005 (0.0004, 0.0006)	0.0002 (0.0001, 0.0003)	0.0005 (0.0004, 0.0006)
$\varphi_{c}$	n/a	n/a	0.1360 (0.0783, 0.1959)	0.0073 (0.0000, 0.0194)
$\varphi_{m}$	n/a	0.5119 (0.4780, 0.5451)	n/a	0.5064 (0.4722, 0.5412)

Note.—Results for the other chromosomes are summarized in supplementary figure S8, Supplementary Material online. “n/a” means the parameter does not exist in the model.

**Table 3.** Bayes Factors for Comparing Four Introgression Models for the *Heliconius* Datasets (fig. 6, table 2), Calculated Using Thermodynamic Integration with 32 or 64 Gaussian Quadrature Points and Savage–Dickey Density Ratio with Threshold ϵ=1%, 0.1%, or 0.01%.

	Thermodynamic Integration		Savage–Dickey Density Ratio		
$B_{ij}$ (Null Hypothesis Tested, $H_{0}$)	32 points	64 points	ϵ=1%	ϵ=0.1%	ϵ=0.01%
*Noncoding loci* (L=5,341 loci)					
$B_{\text{I}Ø}$ ($H_{0}\;:\;\varphi_{C\to M}=0$)	$e^{1087.1}$	$e^{1082.5}$	∞	∞	∞
$B_{\text{O}Ø}$ ($H_{0}\;:\;\varphi_{M\to C}=0$)	$e^{946.9}$	$e^{904.9}$	∞	∞	∞
$B_{\text{BI}}$ ($H_{0}\;:\;\varphi_{M\to C}=0$)	$e^{-5.6}$	$e^{-9.9}$	0.0101	0.0025	0.0020
$B_{\text{BO}}$ ($H_{0}\;:\;\varphi_{C\to M}=0$)	$e^{134.6}$	$e^{167.8}$	∞	∞	∞
$B_{\text{IO}}$ ($H_{0}\;:\;\varphi_{C\to M}=0$ vs. $\varphi_{M\to C}=0$)	$e^{140.2}$	$e^{177.6}$	n/a	n/a	n/a
$B_{\text{B}Ø}$ ($H_{0}\;:\;\varphi_{C\to M}=0$ and $\varphi_{M\to C}=0$)	$e^{1081.6}$	$e^{1072.6}$	∞	∞	∞
*Coding loci* (L=4,942 loci)					
$B_{\text{I}Ø}$ ($H_{0}\;:\;\varphi_{C\to M}=0$)	$e^{359.9}$	$e^{358.5}$	∞	∞	∞
$B_{\text{O}Ø}$ ($H_{0}\;:\;\varphi_{M\to C}=0$)	$e^{128.0}$	$e^{147.6}$	∞	∞	∞
$B_{\text{BI}}$ ($H_{0}\;:\;\varphi_{M\to C}=0$)	$e^{-13.0}$	$e^{-8.6}$	0.0136	0.0090	0.0073
$B_{\text{BO}}$ ($H_{0}\;:\;\varphi_{C\to M}=0$)	$e^{218.9}$	$e^{202.3}$	∞	∞	∞
$B_{\text{IO}}$ ($H_{0}\;:\;\varphi_{C\to M}=0$ vs. $\varphi_{M\to C}=0$)	$e^{231.9}$	$e^{210.9}$	n/a	n/a	n/a
$B_{\text{B}Ø}$ ($H_{0}\;:\;\varphi_{C\to M}=0$ and $\varphi_{M\to C}=0$)	$e^{346.8}$	$e^{349.9}$	∞	∞	∞

Note.—The four models are (Ø) MSC with no gene flow, (I) C→M introgression (I), (O) M→C introgression, and (B) C⇆M bidirectional introgression (table 2). Bayes factor $B_{ij}$ represents the evidence in favor of model *i* against model *j*. We use a cutoff of 1%, so that $B_{ij}>100$ means strong support for model *i* and rejection of model *j*, $B_{ij}<0.01$ means strong support for model *j* and rejection of model *i*, while $0.01<B_{ij}<100$ means no strong preference for either model. The approach based on Savage–Dickey density ratio is inapplicable for $B_{\text{IO}}$ as models I and O are not nested. Also it produces B=∞ if all values of *φ* in the MCMC sample are >ϵ. Results for the other chromosomes are shown in supplementary table S5, Supplementary Material online. “n/a” means the parameter does not exist in the model.

#### Bayesian Test of Introgression for Chromosome 1

Results of the Bayesian test are summarized in table 3. To compare the four different models, we calculated Bayes factors using two approaches: thermodynamic integration with Gaussian quadrature (Lartillot and Philippe 2006; Rannala and Yang 2017) and Savage–Dickey density ratio (Ji et al. 2023); see Materials and Methods. The calculated values of the Bayes factor for the same test varied depending on the number of quadrature points in the thermodynamic-integration approach and on the threshold parameter in the Savage–Dickey density ratio, reflecting the challenges of calculating the marginal likelihoods or Bayes factors reliably in large datasets (Rannala and Yang 2017). For example, $\log\;B_{I⊘}$ for comparison of model I (C→M introgression) against model Ø (no gene flow) was 1087.1 and 1082.5, respectively, when K=32 and 64 quadrature points were used in Gaussian quadrature. This difference is mainly due to the difficulty of calculating the power posterior rather than the use of too few quadrature points (Rannala and Yang 2017). Nevertheless, both values are far greater than the cutoff of 4.6 (=log100). Similarly the Savage–Dickey density ratio approach estimates $B_{I⊘}$ to be ∞ at all three threshold values (ϵ=1%,0.1%,0.01%). Both approaches thus strongly support model I with C→M introgression and reject model Ø with no gene flow.

For both datasets from chromosome 1, the two approaches to Bayes factor calculation lead to the same conclusion, as do the three threshold values for the Savage–Dickey density ratio (ϵ=1%,0.1%,0.01%). The null hypothesis $\varphi_{C\to M}=0$ is rejected in the I-Ø and B-O comparisons, with strong support for the C→M introgression, whether or not the M→C introgression is accommodated in the model.

The B–I comparison tests the null hypothesis $\varphi_{M\to C}=0$ when both the null and alternative models accommodate the C→M introgression. This test leads to strong support for the null model I, with $B_{\text{BI}}<0.01$. With C→M introgression accommodated, the data strongly support the absence of M→C introgression. Unlike Frequentist hypothesis testing, which can never support the null hypothesis strongly, here the Bayesian test strongly favors the null model I, rejecting the more general alternative model B.m

However, the test of $\varphi_{M\to C}=0$ is significant in the O–Ø comparison when the C→M introgression is not accommodated in the null and alternative models. This result mimics our computer simulation, in which the test of gene flow is often significant if the assumed gene flow is in the wrong direction (supplementary figs. S2, S6, and S7, Supplementary Material online).

Models I and O are not nested, but the Bayes factor can be used to compare them. $B_{\text{IO}}$ suggests strong preference for model I (C→M gene flow) over model O (M→C gene flow).

Thus, all tests have led to the same conclusions. Both the coding and noncoding datasets strongly support the presence of *H. cydno*→*H. melpomene* introgression, and both strongly support the absence of the *H. melpomene*→*H. cydno* introgression.

#### Parameter Estimation for Chromosome 1

Bayesian parameter estimates under the four models are summarized in table 2. Consistent with the results of the Bayesian test above, estimates of *φ* under model B suggest that gene flow is unidirectional. The estimates for the noncoding data are ${\hat{\varphi }}_{C\to M}=0.28$ (95% HPD CI: 0.25–0.31) and ${\hat{\varphi }}_{M\to C}<1\%$ in the opposite direction, while for the coding data, they are ${\hat{\varphi }}_{C\to M}=0.51$ (95% HPD CI: 0.47–0.54) and ${\hat{\varphi }}_{M\to C}<1\%$ (table 2). The reasons for the higher rate (${\hat{\varphi }}_{C\to M}$) for the coding than the noncoding data are unknown. One intriguing possibility is that introgression is mostly adaptive, driven by natural selection, and that coding loci are under stronger selection. The time of introgression is nearly zero, suggesting that gene flow may be ongoing. Estimates under model I are nearly identical to those under model B. In model O where only M→C gene flow is allowed, the introgression probability is estimated to be ${\hat{\varphi }}_{M\to C}=0.17$ (0.15,0.20) for the noncoding data, and 0.14 (0.08, 0.20) for the coding data. Those rates are substantial, consistent with the significant test results ($B_{O⊘}$). Even if gene flow is unidirectional from *C* to *M*, assuming introgression in the opposite (and presumably wrong) direction leads to high estimates of the rate and significant test results. Those results again parallel our simulations (supplementary figs. S2, S6, and S7, Supplementary Material online). The misspecified introgression direction in model O causes large estimates of $\theta_{s}$ and reduces $\tau_{s}$. Those results mimic the behaviors of the misspecified model in the large→small case in our theoretical analysis and simulations (fig. 3, supplementary table S1*d*, Supplementary Material online large→small).

We note that the divergence time between *H. cydno* and *H. melpomene* ($\tau_{s}$) is estimated to be much smaller, and $\theta_{S}$ is much larger under model Ø (no gene flow) than under model I or B. This is because ignoring gene flow when it occurs causes model Ø to misinterpret reduced between-species sequence divergence (due to introgression) as more recent species divergence (Leaché et al. 2014; Tiley et al. 2023).

#### Parameter Estimation for the Other Autosomes

We analyzed the coding and noncoding data from all chromosomes in the same way, with parameter estimates under the four models (Ø, I, O, B) summarized in supplementary figure S8, Supplementary Material online (see also supplementary table S5, Supplementary Material online), while Bayesian test results are in supplementary table S6, Supplementary Material online.

There is overall consistency among the autosomes (chromosomes 1–20), although estimates of some parameters from chromosomes 5, 10, 13, 15, and 19 appear as outliers. For example, estimates of $\theta_{C}$ and $\theta_{M}$ are unusually large for chromosomes 5, 15, 19, and 20. A likely explanation is that the *H. melpomene* sample was partially inbred, with large variations in heterozygosity across chromosomes. We discuss results for the autosomes first before dealing with chromosome 21 (the Z chromosome).

For the autosomes, there is overall consistency between the coding and noncoding data: divergence times $\tau_{r}$ and $\tau_{s}$ and population sizes $\theta_{H}$ and $\theta_{r}$ are larger for the noncoding than coding data, by a similar factor across chromosomes (supplementary fig. S8, Supplementary Material online). This can be explained by a reduced effective neutral mutation rate for the coding data, due to purifying selection removing nonsynonymous mutations.

Although model Ø (no gene flow) underestimated the divergence time between the two species involved in gene flow, $\tau_{s}$ (see above), all four models including model Ø produce nearly identical estimates of $\tau_{r}$, indicating that the impact of introgression is local on the species tree, only affecting estimates of parameters for nodes close to the introgression event. Estimates of $\tau_{s}$ under model O are consistently smaller than under models I and B, especially for the coding data, apparently related to the low estimates of $\varphi_{M\to C}$ for the coding data under model O. Introgression time $\tau_{c}=\tau_{m}$ is nearly zero for most chromosomes under models I, O, and B, indicating that gene flow may be ongoing (Huang et al. 2022).

Estimates of introgression probability $\varphi_{C\to M}$ are very similar between models I and B, and they are consistently larger for coding than noncoding data. Estimates of $\varphi_{M\to C}$ under model B are consistently ≈0, suggesting the absence of M → C gene flow. Estimates of $\varphi_{M\to C}$ under model O, assuming introgression in the wrong direction, are always larger than estimates under model B, but vary among chromosomes. These results are consistent with our simulations (e.g., fig. 3, cases **a**–**d**), where estimates of introgression probability $\varphi_{X}$ in model O vary, even though the true rate in the opposite direction is fixed ($\varphi_{Y}=0.2$), influenced by estimates of population sizes such as $\theta_{X}$ and $\theta_{Y}$.

#### Bayesian Test of Introgression for the Autosomes

Bayes factors calculated via the Savage–Dickey density ratio are presented in supplementary table S6, Supplementary Material online. The results are similar to those for chromosome 1, with overwhelming evidence for the C → M introgression and no evidence for M → C introgression. For some datasets, $B_{O⊘}<100$, so that the test of gene flow ($H_{0}\;:\;\varphi_{M\to C}=0$) is not significant when introgression was assumed to be in the wrong direction.

#### Unidentifiability Issues for the Haploid Sex Chromosome

Results for chromosome 21 (the Z chromosome) show very different patterns from the autosomes (supplementary fig. S8, Supplementary Material online), because we have only one haploid sequence per species in the data: both *H. cydno* and *H. melpomene* samples are hemizygous females, i.e., ZW. For such data, some parameters are unidentifiable in any of the four models, such as $\theta_{C},\theta_{M},\theta_{H}$ for the extant species. As discussed before, models I and O are unidentifiable, with the parameter mapping $\varphi_{M}^{\left(I\right)}=\varphi_{C}^{\left(O\right)}$ and $\theta_{c}^{\left(I\right)}=\theta_{m}^{\left(O\right)}$. Thus, those parameters should have exactly the same posterior. This is a case of cross-model unidentifiability.

**Fig. 7.**
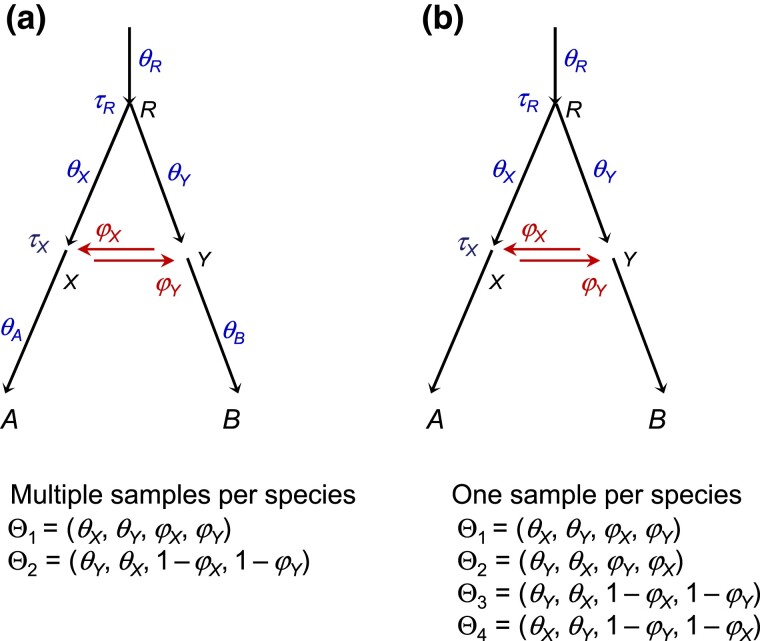
(*a*) When multiple sequences are sampled per species per locus, the MSC-I model with bidirectional introgression between sister lineages has two unidentifiable modes in the posterior ($\Theta_{1},\Theta_{2}$) (Yang and Flouri 2022). Population size parameters for extant species ($\theta_{A},\theta_{B}$) are identifiable. (*b*) When one sequence is sampled per species per locus, the same model shows four unidentifiable modes ($\Theta_{1},\Theta_{2},\Theta_{3},\Theta_{4}$). Also $\theta_{A}$ and $\theta_{B}$ are unidentifiable and are not parameters in the model.

Model B applied to data from the Z chromosome (with one sequence per species per locus) poses an even more complex unidentifiability issue. As discussed later in Discussion, there are four unidentifiable modes in the posterior surface (fig. 7*b*). Due to the symmetry of the posterior surface, the marginal posteriors for $\varphi_{M}$ and $\varphi_{C}$ are identical, as are the posteriors for $\varphi_{M}$ and $1-\varphi_{M}$; as a result, the posterior means of $\varphi_{M}$ and $\varphi_{C}$ are both $\frac{1}{2}$ (supplementary fig. S8, Supplementary Material online). Similarly the posteriors for $\theta_{c}$ and $\theta_{m}$ are identical. Nevertheless, parameters not involved in the unidentifiability (such as $\tau_{r},\tau_{c},\theta_{r}$) are well estimated. In theory, the four modes represent unidentifiability of the label-switching type, and a relabeling algorithm can be used to process the MCMC samples to map the parameter values onto one of the four modes, as in Yang and Flouri (2022). This is not pursued here. Instead, our objective here is to provide explanations for the results of supplementary figure S8, Supplementary Material online (chromosome 21, model B). We recommend that multiple samples per species per locus (in particular from the recipient species) should be used to estimate introgression probabilities. Note that one diploid individual is equivalent to two haploid sequences.

## Discussion

### Inferring the Direction of Gene Flow Using Genomic Data

In this study, we have identified features of genomic sequence data that are informative about the direction of gene flow, and quantified the power of the Bayesian test of gene flow and the precision and biases in estimates of parameters under the MSC-I model such as the time and strength of introgression. Our asymptotic analysis, computer simulation and real data analysis have produced highly consistent results. We have illustrated that one may gain much insight into the workings of likelihood-based inference under the MSC-I model by simply considering pairwise coalescent times ($t_{aa},t_{ab},t_{bb}$) even though these are very simple summaries of the original data of multilocus sequence alignments (table 1). Knowledge of important features in the data that drive the estimation of model parameters, such as the introgression time and introgression probability, is very useful when we interpret results from analysis of real datasets.

Our analyses of both simulated and real data have demonstrated that typical genomic datasets may be very informative about the direction, timing and strength of introgression, and that current Bayesian implementations of the MSC-I model can accommodate thousands of genomic loci and are able to detect gene flow with nearly 100% power and to estimate the introgression time and introgression probability with high precision and accuracy (fig. 3; supplementary figs. S2–S7, Supplementary Material online; see also Thawornwattana et al. 2022; Ji et al. 2023).

One major result from our analysis is that if introgression is assumed to occur in the wrong direction, the Bayesian test of gene flow will often be significant, and Bayesian estimates of introgression rate will typically be nonzero and may even be greater than the true rate in the correct direction. Thus, neither a significant test nor a high rate estimate is reliable evidence that introgression occurred in the specified direction. This result may seem surprising and disturbing given that introgression in the specified direction is nonexistent.

Our analyses of both simulated and real data suggest that the bidirectional model may be applied to infer the introgression direction. If gene flow is truly unidirectional, overparametrization of the bidirectional model appears to incur little cost in statistical performance even though it does add to computational cost: posterior CIs and power to detect gene flow under the bidirectional model are very similar to those under the true unidirectional model.

Of course a better approach to inferring the introgression direction is to implement efficient cross-model MCMC algorithms to search in the space of all MSC-I models for the given set of species. Indeed, MCMC algorithms that move between MSC-I models already exist (Wen and Nakhleh 2018; Zhang et al. 2018). These propose changes to the MSC-I model when the gene trees at all loci are fixed, and if the proposed new model is in conflict with some gene trees, the proposal is abandoned. Such algorithms have poor mixing properties if the dataset is not very small because the proposed new model is very likely to be in conflict with at least some gene trees. The algorithms do not appear to be feasible for analyzing even small datasets with 100 loci (Wen and Nakhleh 2018; Zhang et al. 2018). However, thousands of loci are often needed to provide precise and reliable inference of introgression between species. Smart MCMC moves that make coordinated changes to the gene trees when the chain moves from one model to another—similar to the algorithms developed under the MSC model with no gene flow for updating species divergence times (the rubber-band algorithm, Rannala and Yang 2003) or species phylogenies (the species-tree NNI or SPR moves, Yang and Rannala 2014; Rannala and Yang 2017)—may offer significant improvements even though they are challenging to develop.

Most heuristic methods for detecting gene flow are based on species triplets or quartets and use summaries of sequence data such as genome-wide site-pattern counts (as in the *D*-statistic, Green et al. 2010; Durand et al. 2011 and Hyde, Blischak et al. 2018) or frequencies of estimated gene tree topologies (as in Snaq, Solis-Lemus and Ane 2016). Those methods are agnostic about the direction of gene flow. The $D_{\text{FOIL}}$ method of Pease and Hahn (2015) extends the *D*-statistic to identify the introgression direction: it assumes a particular species phylogeny for five species (a balanced quartet tree plus an outgroup), with one sequence sampled per species per locus. None of those heuristic methods can identify gene flow between sister lineages or its direction. Overall current heuristic methods make use of a small portion of information about gene flow in the multilocus sequence alignments, and offer exciting opportunities for improvements.

### Unidentifiability of Introgression Models

In this study (in particular, during the analysis of the *Heliconius* data), we have encountered several different types of unidentifiability issues. Here, we include a summary, which is technical and can be skipped (see also Yang and Flouri 2022 for further discussions).

Yang and Flouri (2022) distinguished between *within-model* and *cross-model* unidentifiability. If the probability distributions of the data are identical under model *m* with parameters Θ and under model $m^{\prime }$ with parameters Θ′, with


(4) $$f(X\;|\;m,\Theta )=f(X\;|\;m^{\prime },\Theta^{\prime })$$

for all possible data *X*, then data *X* cannot identify (m,Θ) and $\left(m^{\prime },\Theta \prime \right)$. If $m=m^{\prime }$ and Θ≠Θ′, the parameters within the given model are unidentifiable. If $m\neq m^{\prime }$, the two models are unidentifiable (cross-model); in this case there is a parameter mapping from Θ in *m* to Θ′ in $m^{\prime }$.

In the case of two species (say *A* and *B*) with one sequence sampled per species per locus, the coalescent time ($t_{ab}$) between the two sequences (a,b) has the same distribution under model I with A→B introgression and under model O with B→A introgression (Appendix). As a result, the two models are unidentifiable, or in other words, the introgression direction is unidentifiable (Yang and Flouri 2022, fig. 10). This is a case of cross-model unidentifiability. The parameter mapping is $\theta_{Y}^{\left(O\right)}=\theta_{X}^{\left(I\right)}$, $\theta_{R}^{\left(O\right)}=\theta_{R}^{\left(I\right)}$ and $\varphi_{X}=\varphi_{Y}$, with $\tau_{R}$ and $\tau_{X}$ being identical between the two models (eq. A3, fig. 1). In the analysis of chromosome 21 from the *Heliconius*, model I and model O are unidentifiable, with $\theta_{m}^{\left(O\right)}=\theta_{c}^{\left(I\right)},\theta_{s}^{\left(O\right)}=\theta_{s}^{\left(I\right)}$ and $\varphi_{c}=\varphi_{m}$ (supplementary fig. S8 and table S5, Supplementary Material online).

If the model (model I, say) is given, parameters $\tau_{R},\tau_{X},\theta_{X}$, and $\varphi_{Y}$ are identifiable even with data of one sequence per species per locus. In the example of chromosome 21 for the *Heliconius* data, parameters $\tau_{s},\tau_{c},\theta_{c}$, and $\varphi_{m}$ are identifiable (supplementary fig. S8 and table S5, Supplementary Material online).

In the case of two species, introgression direction becomes identifiable if multiple sequences are sampled per species per locus (Yang and Flouri 2022). Furthermore, if data from other species are available and if gene flow occurs between nonsister species, introgression direction affects the distributions of the gene trees and coalescent times, and is identifiable whether one sequence or multiple sequences are sampled per species per locus (Jiao et al. 2021; Hibbins and Hahn 2022; Yang and Flouri 2022).

Furthermore, the bidirectional introgression model (B) poses an *unidentifiability of the label-switching type* (Yang and Flouri 2022). The situation is similar to label switching in clustering analysis. Let the parameter vector be $\Theta =(p_{1},\mu_{1},\mu_{2})$, with two groups in proportions $p_{1}$ and $p_{2}=1-p_{1}$ with means $\mu_{1}$ and $\mu_{2}$. Then Θ and $\Theta^{\prime }=(p_{1}^{\prime },\mu_{1}^{\prime },\mu_{2}^{\prime })=(p_{2},\mu_{2},\mu_{1})$ are unidentifiable as their only difference is in the labels “1” and “2” for the two groups. Such models can still be used in inference. If multiple samples are available per species per locus, model B with introgression between sister lineages shows two unidentifiable modes involving the two introgression probabilities and two population size parameters (Yang and Flouri 2022): in figure 7*a*, $\Theta_{1}=(\theta_{X},\theta_{Y},\varphi_{X},\varphi_{Y})$ and $\Theta_{2}=(\theta_{X},\theta_{Y},\varphi_{X},\varphi_{Y})$ are unidentifiable. This is a within-model unidentifiability of the label-switching type.

The case of the model B with only one sequence per species per locus was not discussed by Yang and Flouri (2022), although it arose in the analysis of data for chromosome 21 in the *Heliconius* genomic data (supplementary fig. S8, Supplementary Material online). With such data, model B with introgression between sister lineages shows four unidentifiable modes in the posterior: in figure 7*b*, $\Theta_{1}=(\theta_{X},\theta_{Y},\varphi_{X},\varphi_{Y})$, $\Theta_{2}=(\theta_{Y},\theta_{X},\varphi_{Y},\varphi_{X})$, $\Theta_{3}=(\theta_{Y},\theta_{X},1-\varphi_{X},1-\varphi_{Y})$, and $\Theta_{4}=(\theta_{X},\theta_{Y},1-\varphi_{Y},1-\varphi_{X})$ are unidentifiable (fig. 7). If introgression is between nonsister lineages, each bidirectional introgression pair will create two cross-model modes, whether one sequence or multiple sequences are sampled per species per locus (Yang and Flouri 2022).

### Asymmetry of Gene Flow in Nature

No systematic studies have examined the frequency of unidirectional versus bidirectional gene flow given that two species are involved in introgression. Both scenarios appear to be common. Sometimes gene flow occurs in one direction even though opportunities exist also in the opposite direction. A well-documented example is gene flow in the *Anopheles gambiae* group of mosquitoes in sub-Saharan Africa (della Torre et al. 1997; Slotman et al. 2005). Analysis of genomic data provides strong evidence for gene flow from *A. arabiensis* to *A. gambiae* or its sister species *A. coluzzii*, while the rate of gene flow in the opposite direction was estimated to be 0 (Thawornwattana et al. 2018; Flouri et al. 2020). This result from comparisons of genomic sequences is consistent with crossing experiments which supported introgression of autosomal regions from *A. arabiensis* into *A. gambiae* but not in the opposite direction (della Torre et al. 1997; Slotman et al. 2005). One possible explanation is that the X chromosome from one species may be incompatible with the autosomal background of the other species (Slotman et al. 2004; Slotman and Powell 2005). The introgression from *A. arabiensis* into the common ancestor of *A. gambiae* and *A. coluzzii* has been hypothesized to have facilitated the range expansion of *A. gambiae* and *A. coluzzii* into the more arid savanna habitats of *A. arabiensis* (Coluzzi et al. 1979; Ayala and Coluzzi 2005).

Note that the rate of gene flow in the MSC-I model estimated from the genomic sequence data is an “effective” rate, reflecting the combined effects of gene flow and natural selection. Most introgressed alleles are expected to be purged in the recipient species by selection because they are deleterious or incompatible with the host genomic background (Schumer et al. 2018; Matute et al. 2020). It seems likely that alleles at introgressed loci from species *A* on the genomic background of species *B* will have different fitnesses than introgressed alleles from *B* on the background of *A*. Another factor is geographic context. If a smaller population of species *A* hybridizes with a larger population of species *B*, *A* is more likely to be swamped by *B*, making introgression asymmetrical. With all those factors considered, one should expect gene flow to be asymmetrical in most systems, with different rates in the two directions.

### Gene Flow in *Heliconius* Butterflies

*Heliconius cydno* and *H. melpomene* are broadly sympatric across Central America and northwestern South America, and are known to hybridize in the wild (Mallet et al. 2007). Our analysis supports recent unidirectional gene flow from *H. cydno* into *H. melpomene* (fig. 6, tables 2 and 3; supplementary tables S5 and S6, Supplementary Material online), in Panama, where *H. cydno chioneus* and *H. melpomene rosina* are broadly sympatric. In captivity, male F${}_{1}$ hybrids are fertile while female F${}_{1}$ hybrids are sterile; male hybrids backcross to either parental species much more readily than the pure species mate with one another (Naisbit et al. 2001, 2002).

Previous studies used different approaches to estimate gene flow between these two species. Early phylogenetic analyses of multilocus data attributed recent gene flow between *H. cydno chioneus* and *H. melpomene rosina* as a cause for gene tree variation among loci (Beltrán et al. 2002). An IM analysis (Hey and Nielsen 2004) using a small number of loci yielded an estimated symmetric bidirectional migration rate *m* between the two species of $1.7\times 10^{-6}$ (95% CI $1.0-45\times 10^{-6}$) per generation, with *H. cydno chioneus* having a larger effective population size (Bull et al. 2006). An IM model allowing for different migration rates in each direction found evidence for unidirectional gene flow from *H. cydno* into *H. melpomene*, with $2N_{M}m_{C\to M}=0.294$ (90% HPD CI: 0.116–0.737), whereas $2N_{C}m_{M\to C}=0.000$ (0.000, 0.454) (Kronforst et al. 2006), consistent with our results. Similar patterns were obtained in a subsequent *IMa2* analysis (Hey 2010) of a larger dataset (Kronforst et al. 2013). In a more recent analysis of genome-scale data, Martin et al. (2015) estimated a symmetric bidirectional migration rate between *H. c. chioneus* and *H. m. rosina* to be $\hat{M}=0.20$ (90% HPD interval: 0.09–0.40) per generation. Lohse et al. (2016) compared three models: complete isolation after divergence, and two IM models with unidirectional gene flow, and preferred the model with gene flow from *H. cydno* into *H. m. rosina*, with estimated migration rate 4Nm=1.5. Martin et al. (2019) used gene tree frequencies to suggest extensive gene flow from *H. cydno* into *H. melpomene* in Panama.

Our estimates are in general consistent among chromosomes and between coding and noncoding data. However, only one diploid individual per species is included in the genomic data, with some from inbred lines (selected for sequencing because of easy assembly). These features of the data may have affected our estimates and account for the outlier estimates observed for a few chromosomes (supplementary fig. S8, Supplementary Material online). Overall, our analyses of genomic data are consistent with previous estimates.

We note that the null model ⊘ in the Bayesian test used in this study constrains the population sizes ($\theta_{C}=\theta_{c}$ and $\theta_{M}=\theta_{m}$) as well as the introgression probability (φ=0), compared with the alternative model (models I, O, or B) (Ji et al. 2023). Rejection of the null model may in theory be due to either introgression or inequality of population sizes, or both. A sharper test may use an alternative model with the same constraints on the population sizes as in the null model ($\theta_{C}=\theta_{c}$, $\theta_{M}=\theta_{m}$) so that the two models under comparison have the only difference concerning the introgression probability (φ=0 vs. φ>0); this is test 2 in Ji et al. (2023, fig. 3). For the *Heliconius* data, we note that the CIs for $\varphi_{c}$ exclude the null value $\varphi_{c}=0$ for every autosome (supplementary fig. S8, Supplementary Material online), providing strong evidence for some introgression in the minority direction. Furthermore, it may be interesting to examine the impact of priors on parameters on the Bayesian test (Ji et al. 2023). We leave it to future work to use more genomic data and more focused tests to infer gene flow in this group of *Heliconius* butterflies.

## Materials and Methods

### Asymptotic Analysis and Simulation in the Case of Two Species

We examined the distributions of coalescent times and conducted computer simulations under model I of figure 1*a*, with A→B introgression. We used four sets of parameter values.

- same *θ* tall tree: all populations have the same size with θ=0.01. The other parameters are $\tau_{R}=\theta ,\tau_{X}=0.5\theta$, and $\varphi_{Y}=0.2$.
- same *θ* short tree: θ=0.01 for all populations, $\tau_{R}=0.5\theta ,\tau_{X}=0.25\theta$, and $\varphi_{Y}=0.2$.
- small to large: different species on the species tree have different population sizes, with $\theta_{A}=\theta_{X}=\theta_{R}=\theta_{0}=0.002$ on the left of the tree and $\theta_{B}=\theta_{Y}=\theta_{1}=0.01$ on the right, with introgression from a small population to a large one (fig. 1*a*). Other parameters are $\tau_{R}=3\theta_{0},\tau_{X}=1.5\theta_{0}$, and $\varphi_{Y}=0.2$.
- large to small: This is the same as case (**c**) except that $\theta_{A}=\theta_{X}=\theta_{R}=\theta_{0}=0.01$ on the left of the tree and $\theta_{B}=\theta_{Y}=\theta_{1}=0.002$ on the right, so that introgression is from a large population to a small one.

We simulated multilocus sequence datasets under model I (fig. 1*a*) and analyzed them under models I, O, and B (fig. 1*a*–*c*). Each replicate dataset consisted of L=250, 1,000 or 4,000 loci, with n=4 sequences sampled per species per locus. The sequence length is N=500 sites. The simulate option of Bpp (Flouri et al. 2018) was used to simulate gene trees with coalescent times and to “evolve” sequences along the gene tree under the JC model (Jukes and Cantor 1969). Sequences at the tips of the gene tree constitute the data. The number of replicates was 100.

Each replicate dataset was then analyzed using Bpp (Flouri et al. 2018, 2020) under models I, O, and B of figure 1*a*–*c*. This setting in which the model is fixed corresponds to the A00 analysis of (Yang 2015). The JC model was assumed in the analysis. Gamma priors were assigned to the age of the root of the species tree ($\tau_{R}$) and to population size parameters (*θ*), with the shape parameter α=2 so that the prior was diffuse and with the rate parameter β chosen so that the prior mean was close to the true values. We used $\tau_{R}∼G(2,200)$ and θ∼G(2,200) for case **a** “same *θ* tall tree”; $\tau_{R}∼G(2,400)$ and θ∼G(2,200) for case **b** “same *θ* short tree”; $\tau_{R}∼G(2,400)$ and θ∼G(2,400) for case **c** “small to large” and **d** “large to small.” Introgression probability *φ* was assigned the beta prior beta(1,1), which is U(0,1).

MCMC settings were chosen by performing pilot runs, with MCMC convergence assessed by verifying consistency between replicate runs for the same analysis. The same setting was then used to analyze all replicate datasets. We used 16,000 MCMC iterations as burnin, and then took $10^{5}$ samples, sampling every 2 iterations. Running time for analyzing one replicate dataset was ∼45 min for L=250 loci or ∼3 h for L=1,000 using one thread, and ∼12 h for L=4,000 using two threads.

### Simulation to Evaluate the Gain in Information for Estimating *φ* by Adding a Third Species

Given the introgression model for two species (A,B) of figure 1*a*, with A→B introgression, we added a third species (*C*) and assessed the gain in information for estimating *φ*. There are five branches on the two-species tree, to which the third species could be attached (fig. 4*a*–*e*): (**a**) the root population, (**b**, **c**) the source and target populations before gene flow, and (**d**, **e**) the source and target populations after gene flow. In all cases φ=0.2. The original two-species tree had $\tau_{R}=\theta_{1}$ and $\tau_{X}=\theta_{1}/2$. In cases **b**–**e**, species *C* was attached to the midpoint of the target branch, while in **a**, the new root was 1.25× as old as the old root. For models **a**, **d**, and **e**, all populations on the species tree had the same size, with $\theta_{1}=0.01$. For cases **b** and **c**, three scenarios were considered: 1) equal population size, with $\theta_{1}=0.01$ for all populations; 2) from small to large, with $\theta_{A}=\theta_{X}=\theta_{S}=\theta_{0}=0.002$ for the thin branches in case **b** and $\theta_{A}=\theta_{X}=\theta_{0}=0.002$ in case **c** and with $\theta_{1}=0.01$ for all other branches; and 3) from large to small, with $\theta_{B}=\theta_{Y}=\theta_{0}=0.002$ in case **b** and $\theta_{B}=\theta_{Y}=\theta_{S}=\theta_{0}=0.002$ in case **c** and with $\theta_{1}=0.01$ for all other branches. For each parameter setting, we simulated 100 replicate datesets. Each dataset consisted of L=1,000 loci, with $n_{A}=n_{B}=4$ sequences per species per locus and N=500 sites in the sequence. Each dataset was analyzed using Bpp to estimate the parameters in the MSC-I model (fig. 4*a*–*e*). Gamma priors were assigned to $\tau_{R}$ and *θ*: $\tau_{R}∼G(2,200)$ and θ∼G(2,200), while $\varphi_{A\to B}∼U(0,1)$. We used 32,000 MCMC iterations as burnin, and then took $10^{6}$ samples, sampling every 10 iterations. Running time for analyzing one dataset using one thread was ∼30 h.

### Simulation in the Case of Four Species: Inflow Versus Outflow

We simulated data under the three MSC-I models (I, O, B) of figure 5*a*–*c*, with introgression between nonsister species *A* and *B* on a four-species tree ((A,(B,C)),D). The three models differ in the assumed direction of gene flow, with I for inflow from *A* to *B*, O for outflow from *B* to *A*, and B for bidirectional introgression between *A* and *B*. We used two sets of parameter values. In the first set (same-*θ*), all species on the tree had the same population size, with $\theta_{0}=\theta_{1}=0.01$. In the second set (different-*θ*), the thin branches had $\theta_{0}=0.002$ while the thick branches had $\theta_{1}=0.01$ (fig. 5*a*–*c*). Other parameters were the same in the two settings, with $\tau_{R}=4\theta_{0}$, $\tau_{S}=3\theta_{0}$, $\tau_{T}=2\theta_{0}$, and $\tau_{X}=\tau_{Y}=1.5\theta_{0}$, and the introgression probabilities were $\varphi_{X}=\varphi_{Y}=0.2$.

Each dataset consists of L=250, 1,000, or 4,000 loci, with n=4 sequences per species per locus and with N=500 sites in the sequence. The number of replicates was 100. With three MSC-I models (I, O, B), two population-size settings (same-*θ* vs. different-*θ*), and three data sizes (*L*), a total of 3×2×3×100=1800 datasets were generated. Each dataset was analyzed under the three models (I, O, B). Gamma priors were assigned to $\tau_{R}$ and *θ*: $\tau_{R}∼G(2,200)$ and θ∼G(2,400), while φ∼U(0,1). We used 32,000 MCMC iterations as burnin, and took $2\times 10^{5}$ samples, sampling every 5 iterations. Running time for analyzing one dataset was ∼12 h for small datasets of L=250 loci and 60 h for L=1,000 using one thread, and ∼120 h for L=4,000 using two threads.

### Analysis of the *Heliconius* Butterfly Dataset

We processed the raw genomic sequencing data of Edelman et al. (2019) from three species of *Heliconius* butterflies, *H. hecale* (*H*), *H. cydno* (*C*), and *H. melpomene* (*M*), to retrieve coding and noncoding loci for each chromosome, following the procedure of Thawornwattana et al. (2022). See supplementary table S4, Supplementary Material online for the number of loci in each of the 22 datasets. Each locus consisted of one unphased diploid sequence per species, except the Z chromosome (chromosome 21) for which only a haploid sequence is available per species (from ZW females). Heterozygote phase in the diploid sequence was resolved using an analytical integration algorithm in the likelihood calculation in Bpp (Gronau et al. 2011; Flouri et al. 2018; Huang et al. 2022). We fitted four MSC-I models with different introgression directions: (Ø) MSC with no gene flow, (I) C→M introgression, (O) M→C introgression, and (B) C⇆M bidirectional introgression.

We assigned priors $\tau_{r}∼G(4,200)$, θ∼G(2,200), and φ∼U(0,1). We used $10^{5}$ MCMC iterations for burnin, and recorded $10^{4}$ samples, sampling every 100 iterations. For each model, we performed ten independent runs to confirm consistency between runs. The resulting MCMC samples were combined to produce final posterior estimates. Each run took ∼100 h.

### Bayesian Test of Introgression

We applied the Bayesian test of introgression (Ji et al. 2023) to data for two species simulated under the models of figure 1*a*–*c*, the data for four species simulated under models I, O, and B of figure 5, and the *Heliconius* datasets (fig. 6).

Bayesian model selection was used to compare the null model of no gene flow $H_{0}\;:\;\varphi =0$ and the alternative model of introgression $H_{1}\;:\;\varphi >0$. The Bayes factor was calculated as $B_{10}=\frac{M_{1}}{M_{0}}$, where $M_{0}$ and $M_{1}$ are marginal likelihood values under $H_{0}$ and $H_{1}$, respectively. If the prior model probabilities are $\pi_{0}$ and $\pi_{1}$, $B_{10}$ can be converted into posterior model probabilities as $\frac{P(H_{1}\;|\;X)}{P(H_{0}\;|\;X)}=\frac{\pi_{1}}{\pi_{0}}\cdot B_{10}$. If $\pi_{0}=\pi_{1}$, $B_{10}=100$ will translate to the posterior probability $P(H_{0}|X)\approx 1\%$. Thus, $B_{10}>100$ may be considered strong evidence in support of $H_{1}$ over $H_{0}$, while $B_{10}<0.01$ is strong evidence in favor of $H_{0}$ over $H_{1}$.

As $H_{0}$ and $H_{1}$ are nested, $B_{10}$ can be calculated using the Savage–Dickey density ratio (Dickey 1971), by using an MCMC sample under $H_{1}$ (Ji et al. 2023). Define an interval of null effects, ⊘:φ<ϵ, inside which the introgression probability is so small that introgression may be considered nonexistent. The Bayes factor in favor of $H_{1}$ over $H_{0}$ is then


(5) $$B_{10,\epsilon }=\frac{P(\;⊘\;)}{P(\;⊘\;|\;X)},$$

where P(⊘) is the prior probability of the null interval, while P(⊘|X) is the posterior probability, both calculated under $H_{1}$ (Ji et al. 2023). Note that P(⊘)=P(φ<ϵ)=ϵ if the prior is φ∼U(0,1). When ϵ→0, $B_{10,\epsilon }\to B_{10}$ (Ji et al. 2023). We used a few values for ϵ in the range 0.01–1% to assess its effect. This approach has a computational advantage as it requires running the MCMC under $H_{1}$ only and avoids trans-model MCMC algorithms or calculation of marginal likelihood values.

For the *Heliconius* datasets, we in addition used thermodynamic integration combined with Gaussian quadrature to calculate the marginal likelihood under each model, using 32 or 64 quadrature points (Lartillot and Philippe 2006; Rannala and Yang 2017). This approach applies even if the compared models are nonnested, and was used to conduct pairwise comparisons among all four models fitted to the *Heliconius* data.

## Supplementary Material

msad178_Supplementary_Data

## Data Availability

The Heliconius multilocus alignment data are available in Zenodo at https://dx.doi.org/10.5281/zenodo.8243142.

## Appendix The Distribution of Coalescent Times Under the MSC-I Model for Two Species

Here, we gave the probability densities of coalescent times ($t_{aa},t_{ab},t_{bb}$) between two sequences sampled from species *A* and *B* under the MSC-I models I, O, and B of figure 1*a*–*c*. These are simple cases of the gene-tree densities given by, for example, Yu et al. (2014; see also Lohse and Frantz 2014). Example densities under models I and O are plotted in figure 2 for four sets of parameter values.

Under model I,

(*A*1)
$$f_{I}(t_{aa})=\left\{ \begin{array}{ll} \frac{2}{\theta_{A}}\;e^{-\frac{2}{\theta_{A}}t_{aa}}, & \text{if }0<t_{aa}<\tau_{X},\\ e^{-\frac{2}{\theta_{A}}\tau_{X}}\frac{2}{\theta_{X}}\;e^{-\frac{2}{\theta_{X}}(t_{aa}-\tau_{X})}, & \text{if }\tau_{X}<t_{aa}<\tau_{R},\\ e^{-\frac{2}{\theta_{A}}\tau_{X}}\;e^{-\frac{2}{\theta_{X}}(\tau_{R}-\tau_{X})}\\ \;\times \frac{2}{\theta_{R}}\;e^{-\frac{2}{\theta_{R}}(t_{aa}-\tau_{R})}, & \text{if }t_{aa}>\tau_{R}. \end{array}\right.$$

This is a function of $\tau_{R},\tau_{X},\theta_{A},\theta_{X},\theta_{R}$, independent of $\theta_{B},\theta_{Y},\varphi_{Y}$. From the viewpoint of the two *A* sequences, there are demographic changes in population size with $\theta_{A},\theta_{X}$, and $\theta_{R}$, respectively, for the three time segments ($0,\tau_{X}$), ($\tau_{X},\tau_{R}$), and ($\tau_{R},\infty$).

The coalescent time between two sequences sampled from species *B* has the distribution

(*A*2)
$$f_{I}(t_{bb})=\left\{ \begin{array}{ll} \frac{2}{\theta_{B}}\;e^{-\frac{2}{\theta_{B}}t_{bb}}, & \text{if }0<t_{bb}<\tau_{X},\\ e^{-\frac{2}{\theta_{B}}\tau_{X}}[(1-\varphi_{Y}{)}^{2}\frac{2}{\theta_{Y}}\;e^{-\frac{2}{\theta_{Y}}(t_{bb}-\tau_{X})} & \\ \;+\varphi_{Y}^{2}\frac{2}{\theta_{X}}\;e^{-\frac{2}{\theta_{X}}(t_{bb}-\tau_{X})}], & \text{if }\tau_{X}<t_{bb}<\tau_{R},\\ e^{-\frac{2}{\theta_{B}}\tau_{X}}[(1-\varphi_{Y}{)}^{2}\;e^{-\frac{2}{\theta_{Y}}(\tau_{R}-\tau_{X})} & \\ \;+\varphi_{Y}^{2}\;e^{-\frac{2}{\theta_{X}}(\tau_{R}-\tau_{X})}+2\varphi_{Y}(1-\varphi_{Y})] & \\ \;\times \frac{2}{\theta_{R}}\;e^{-\frac{2}{\theta_{R}}(t_{bb}-\tau_{R})}, & \text{if }t_{bb}>\tau_{R}. \end{array}\right.$$

This is a function of $\tau_{R},\tau_{X},\theta_{B},\theta_{X},\theta_{Y},\theta_{R},\varphi_{Y}$, and is independent of $\theta_{A}$. In the time interval ($0,\tau_{X}$), the two *B* sequences coalesce at the rate $2/\theta_{B}$, as in the case of no gene flow. Coalescence during the time interval $\tau_{X}<t_{bb}<\tau_{R}$ can occur in either *X* or *Y*. The former occurs if both *B* sequences migrate to *X* (which occurs with probability $\varphi_{Y}^{2}$) and then coalesce in *X* at the rate $2/\theta_{X}$, whereas the latter occurs when both *B* sequences fail to migrate and thus stay in *Y* (with probability $(1-\varphi_{Y}{)}^{2}$) and then coalesce in *Y* at the rate $2/\theta_{Y}$. If one of the *B* sequences migrates to *X* and the other stays in *Y*, coalescence will be impossible, resulting in a suppression of coalescent events in this time interval (see fig. 2 for $f(t_{bb})$). If the two *B* sequences do not coalesce in *B*, and they do not coalesce in either *X* or *Y*, they will coalesce in species *R* (with $t_{bb}>\tau_{R}$), at the rate $2/\theta_{R}$.

Finally,

(*A*3)
$$f_{I}(t_{ab})=\left\{ \begin{array}{ll} \varphi_{Y}\frac{2}{\theta_{X}}\;e^{-\frac{2}{\theta_{X}}(t_{ab}-\tau_{X})}, & \text{if }\tau_{X}<t_{ab}<\tau_{R},\\ \left[(1-\varphi_{Y})+\varphi_{Y}\;e^{-\frac{2}{\theta_{X}}(\tau_{R}-\tau_{X})}\right] & \\ \;\times \frac{2}{\theta_{R}}e^{-\frac{2}{\theta_{R}}(t_{ab}-\tau_{R})}, & \text{if }t_{ab}>\tau_{R}. \end{array}\right.$$

This is a function of $\tau_{R},\tau_{X},\theta_{X},\theta_{R}$, and $\varphi_{Y}$, and is independent of $\theta_{A},\theta_{B},\theta_{Y}$. Coalescence between *a* and *b* may occur during ($\tau_{X},\tau_{R}$) at the rate $2/\theta_{X}$ if the *B* sequence migrates into *X* (with probability $\varphi_{Y}$).

Under model O with B→A introgression (fig. 1*b*), $f_{O}(t_{aa})$ and $f_{O}(t_{bb})$ are given by $f_{I}(t_{bb})$ and $f_{I}(t_{aa})$ with a change of symbols. In particular, $f_{O}(t_{ab})=f_{I}(t_{ab})$ if $\theta_{Y}^{\left(O\right)}=\theta_{X}^{\left(I\right)}$ and $\varphi_{X}=\varphi_{Y}$, with $\tau_{R},\tau_{X},\theta_{R}$ being identical between the two models.

Under model B with both A→B and B→A introgressions (fig. 1*c*), $f_{B}(t_{aa})=f_{O}(t_{aa})$ and $f_{B}(t_{bb})=f_{I}(t_{bb})$, while

(*A*4)
$$f_{B}(t_{ab})=\left\{ \begin{array}{l} (1-\varphi_{X})\varphi_{Y}\frac{2}{\theta_{X}}\;e^{-\frac{2}{\theta_{X}}(t_{ab}-\tau_{X})}\\ \;+\varphi_{X}(1-\varphi_{Y})\frac{2}{\theta_{Y}}\;e^{-\frac{2}{\theta_{Y}}(t_{ab}-\tau_{X})},\;\text{if }\tau_{X}<t_{ab}<\tau_{R},\\ {}[(1-\varphi_{X})(1-\varphi_{Y})+\varphi_{X}\varphi_{Y}\\ \;+(1-\varphi_{X})\varphi_{Y}\;e^{-\frac{2}{\theta_{X}}(\tau_{R}-\tau_{X})}\\ \;+\varphi_{X}(1-\varphi_{Y})\;e^{-\frac{2}{\theta_{Y}}(\tau_{R}-\tau_{X})}]\\ \;\times \frac{2}{\theta_{R}}\;e^{-\frac{2}{\theta_{R}}(t_{ab}-\tau_{R})},\;\;\text{if }t_{ab}>\tau_{R}. \end{array}\right.$$

## References

[msad178-B1] Arnold ML , Kunte K. 2017. Adaptive genetic exchange: a tangled history of admixture and evolutionary innovation. Trends Ecol Evol. 32(8):601–611. 28645486 10.1016/j.tree.2017.05.007

[msad178-B2] Ayala FJ , Coluzzi M. 2005. Chromosome speciation: humans, *Drosophila*, and mosquitoes. Proc Natl Acad Sci U S A. 102(Suppl 1):6535–6542. 15851677 10.1073/pnas.0501847102 PMC1131864

[msad178-B3] Barton N , Bengtsson BO. 1986. The barrier to genetic exchange between hybridising populations. Heredity 57(3):357–376. 3804765 10.1038/hdy.1986.135

[msad178-B4] Beltrán M , Jiggins CD, Bull V, Linares M, Mallet J, McMillan WO, Bermingham E. 2002. Phylogenetic discordance at the species boundary: comparative gene genealogies among rapidly radiating *Heliconius* butterflies. Mol Biol Evol. 19(12):2176–2190. 12446809 10.1093/oxfordjournals.molbev.a004042

[msad178-B5] Blischak PD , Chifman J, Wolfe AD, Kubatko LS. 2018. HyDe: a Python package for genome-scale hybridization detection. Syst Biol. 67(5):821–829. 29562307 10.1093/sysbio/syy023 PMC6454532

[msad178-B6] Bull V , Beltrán M, Jiggins CD, McMillan WO, Bermingham E, Mallet J. 2006. Polyphyly and gene flow between non-sibling *Heliconius* species. BMC Biol. 4(1):11. 16630334 10.1186/1741-7007-4-11 PMC1481601

[msad178-B7] Campbell CR , Poelstra JW, Yoder AD. 2018. What is speciation genomics? The roles of ecology, gene flow, and genomic architecture in the formation of species. Biol J Linn Soc. 124(4):561–583.

[msad178-B8] Coluzzi M , Sabatini A, Petrarca V, Di Deco MA. 1979. Chromosomal differentiation and adaptation to human environments in the *Anopheles gambiae* complex. Trans R Soc Trop Med Hyg. 73(5):483–497. 394408 10.1016/0035-9203(79)90036-1

[msad178-B9] Coyne JA , Orr HA. 2004. Speciation. Sunderland (MA): Sinauer Associates.

[msad178-B10] della Torre A , Merzagora L, Powell J, Coluzzi M. 1997. Selective introgression of paracentric inversions between two sibling species of the *Anopheles gambiae* complex. Genetics 146(1):239–244. 9136013 10.1093/genetics/146.1.239 PMC1207938

[msad178-B11] Dickey JM . 1971. The weighted likelihood ratio, linear hypotheses on normal location parameters. Ann Math Stat. 42(1):204–223.

[msad178-B12] Durand EY , Patterson N, Reich D, Slatkin M. 2011. Testing for ancient admixture between closely related populations. Mol Biol Evol. 28:2239–2252. 21325092 10.1093/molbev/msr048 PMC3144383

[msad178-B13] Edelman NB , Frandsen PB, Miyagi M, Clavijo B, Davey J, Dikow RB, García-Accinelli G, Van Belleghem SM, Patterson N, Neafsey DE, et al. 2019. Genomic architecture and introgression shape a butterfly radiation. Science 366(6465):594–599. 31672890 10.1126/science.aaw2090 PMC7197882

[msad178-B14] Edelman N , Mallet J. 2021. Prevalence and adaptive impact of introgression. Annu Rev Genet. 55(1):265–283. 34579539 10.1146/annurev-genet-021821-020805

[msad178-B15] Feurtey A , Stukenbrock EH. 2018. Interspecific gene exchange as a driver of adaptive evolution in fungi. Annu Rev Microbiol. 72:377–398. 29927707 10.1146/annurev-micro-090817-062753

[msad178-B16] Flouri T , Jiao X, Rannala B, Yang Z. 2018. Species tree inference with BPP using genomic sequences and the multispecies coalescent. Mol Biol Evol. 35(10):2585–2593. 30053098 10.1093/molbev/msy147 PMC6188564

[msad178-B17] Flouri T , Jiao X, Rannala B, Yang Z. 2020. A Bayesian implementation of the multispecies coalescent model with introgression for phylogenomic analysis. Mol Biol Evol. 37(4):1211–1223. 31825513 10.1093/molbev/msz296 PMC7086182

[msad178-B18] Green RE , Krause J, Briggs AW, Maricic T, Stenzel U, Kircher M, Patterson N, Li H, Zhai W, Fritz MH-Y, et al. 2010. A draft sequence of the Neandertal genome. Science 328:710–722. 20448178 10.1126/science.1188021 PMC5100745

[msad178-B19] Gronau I , Hubisz MJ, Gulko B, Danko CG, Siepel A. 2011. Bayesian inference of ancient human demography from individual genome sequences. Nat Genet. 43:1031–1034. 21926973 10.1038/ng.937 PMC3245873

[msad178-B20] Hey J . 2010. Isolation with migration models for more than two populations. Mol Biol Evol. 27:905–920. 19955477 10.1093/molbev/msp296 PMC2877539

[msad178-B21] Hey J , Chung Y, Sethuraman A, Lachance J, Tishkoff S, Sousa VC, Wang Y. 2018. Phylogeny estimation by integration over isolation with migration models. Mol Biol Evol. 35(11):2805–2818. 30137463 10.1093/molbev/msy162 PMC6231491

[msad178-B22] Hey J , Nielsen R. 2004. Multilocus methods for estimating population sizes, migration rates and divergence time, with applications to the divergence of *Drosophila pseudoobscura* and *D. persimilis*. Genetics 167:747–760. 15238526 10.1534/genetics.103.024182 PMC1470901

[msad178-B23] Hibbins MS , Hahn MW. 2022. Phylogenomic approaches to detecting and characterizing introgression. Genetics. 220(2): iyab173. doi:10.1093/genetics/iyab173 34788444 PMC9208645

[msad178-B24] Huang J , Flouri T, Yang Z. 2020. A simulation study to examine the information content in phylogenomic datasets under the multispecies coalescent model. Mol Biol Evol. 37(11):3211–3224. 32642765 10.1093/molbev/msaa166

[msad178-B25] Huang J , Thawornwattana Y, Flour T, Mallet J, Yang Z. 2022. Inference of gene flow between species under misspecified models. Mol Biol Evol. 39(12):msac237. 36317198 10.1093/molbev/msac237 PMC9729068

[msad178-B26] Ji J , Jackson DJ, Leache AD, Yang Z. 2023. Power of Bayesian and heuristic tests to detect cross-species introgression with reference to gene flow in the *Tamias quadrivittatus* group of North American chipmunks. Syst Biol. 72(2):446–465. 36504374 10.1093/sysbio/syac077 PMC10275556

[msad178-B27] Jiao X , Flouri T, Yang Z. 2021. Multispecies coalescent and its applications to infer species phylogenies and cross-species gene flow. Nat Sci Rev. 8:nwab127. doi:10.1093/nsr/nwab127 PMC8692950 34987842

[msad178-B28] Jukes T , Cantor C. 1969. Evolution of protein molecules. In Munro H, editor. Mammalian protein metabolism. New York: Academic Press. p. 21–123.

[msad178-B29] Kronforst MR , Hansen ME, Crawford NG, Gallant JR, Zhang W, Kulathinal RJ, Kapan DD, Mullen SP. 2013. Hybridization reveals the evolving genomic architecture of speciation. Cell Rep. 5(3):666–677. 24183670 10.1016/j.celrep.2013.09.042 PMC4388300

[msad178-B30] Kronforst MR , Young LG, Blume LM, Gilbert LE. 2006. Multilocus analyses of admixture and introgression among hybridizing *Heliconius* butterflies. Evolution 60(6):1254–1268. 16892975

[msad178-B31] Lartillot N , Philippe H. 2006. Computing Bayes factors using thermodynamic integration. Syst Biol. 55:195–207. 16522570 10.1080/10635150500433722

[msad178-B32] Leaché AD , Harris RB, Rannala B, Yang Z. 2014. The influence of gene flow on Bayesian species tree estimation: a simulation study. Syst Biol. 63(1):17–30. 23945075 10.1093/sysbio/syt049

[msad178-B33] Lohse K , Chmelik M, Martin SH, Barton NH. 2016. Efficient strategies for calculating blockwise likelihoods under the coalescent. Genetics 202(2):775–786. 26715666 10.1534/genetics.115.183814 PMC4788249

[msad178-B34] Lohse K , Frantz LAF. 2014. Neandertal admixture in Eurasia confirmed by maximum likelihood analysis of three genomes. Genetics 196(4):1241–1251. 24532731 10.1534/genetics.114.162396 PMC3982695

[msad178-B35] Mallet J , Beltrán M, Neukirchen W, Linares M. 2007. Natural hybridization in heliconiine butterflies: the species boundary as a continuum. BMC Evol Biol. 7(1):28. 17319954 10.1186/1471-2148-7-28 PMC1821009

[msad178-B36] Marques DA , Meier JI, Seehausen O. 2019. A combinatorial view on speciation and adaptive radiation. Trends Ecol Evol. 34(6):531–544. 30885412 10.1016/j.tree.2019.02.008

[msad178-B37] Martin SH , Dasmahapatra KK, Nadeau NJ, Salazar C, Walters JR, Simpson F, Blaxter M, Manica A, Mallet J, Jiggins CD. 2013. Genome-wide evidence for speciation with gene flow in *Heliconius* butterflies. Genome Res. 23(11):1817–1828. 24045163 10.1101/gr.159426.113 PMC3814882

[msad178-B38] Martin SH , Davey JW, Salazar C, Jiggins CD. 2019. Recombination rate variation shapes barriers to introgression across butterfly genomes. PLoS Biol. 17(2):e2006288. 30730876 10.1371/journal.pbio.2006288 PMC6366726

[msad178-B39] Martin SH , Eriksson A, Kozak KM, Manica A, Jiggins CD. 2015. Speciation in *Heliconius* butterflies: minimal contact followed by millions of generations of hybridisation. bioRxiv.

[msad178-B40] Martin SH , Jiggins CD. 2017. Interpreting the genomic landscape of introgression. Curr Opin Genet Dev. 47:69–74. 28923541 10.1016/j.gde.2017.08.007

[msad178-B41] Matute DR , Comeault AA, Earley E, Serrato-Capuchina A, Peede D, Monroy-Eklund A, Huang W, Jones CD, Mackay TFC, Coyne JA. 2020. Rapid and predictable evolution of admixed populations between two *Drosophila* species pairs. Genetics 214(1):211–230. 31767631 10.1534/genetics.119.302685 PMC6944414

[msad178-B42] Moran BM , Payne C, Langdon Q, Powell DL, Brandvain Y, Schumer M. 2021. The genomic consequences of hybridization. eLife 10:e69016. 34346866 10.7554/eLife.69016 PMC8337078

[msad178-B43] Naisbit RE , Jiggins CD, Linares M, Salazar C, Mallet J. 2002. Hybrid sterility, Haldane’s rule and speciation in *Heliconius cydno* and *H. melpomene*. Genetics 161(4):1517–1526. 12196397 10.1093/genetics/161.4.1517 PMC1462209

[msad178-B44] Naisbit RE , Jiggins CD, Mallet J. 2001. Disruptive sexual selection against hybrids contributes to speciation between *Heliconius cydno* and *Heliconius melpomene*. Proc R Soc Lond B. 268(1478):1849–1854. 10.1098/rspb.2001.1753 PMC1088818 11522205

[msad178-B45] Nielsen R , Wakeley J. 2001. Distinguishing migration from isolation: a Markov chain Monte Carlo approach. Genetics 158:885–896. 11404349 10.1093/genetics/158.2.885 PMC1461674

[msad178-B46] Pease JB , Hahn MW. 2015. Detection and polarization of introgression in a five-taxon phylogeny. Syst Biol. 64(4):651–662. 25888025 10.1093/sysbio/syv023

[msad178-B47] Peters KJ , Myers SA, Dudaniec RY, O’Connor JA, Kleindorfer S. 2017. Females drive asymmetrical introgression from rare to common species in Darwin’s tree finches. J Evol Biol. 30(11):1940–1952. 28833876 10.1111/jeb.13167

[msad178-B48] Petry D . 1983. The effect on neutral gene flow of selection at a linked locus. Theor Popul Biol. 23:300–313. 6623407 10.1016/0040-5809(83)90020-5

[msad178-B49] Rannala B , Yang Z. 2003. Bayes estimation of species divergence times and ancestral population sizes using DNA sequences from multiple loci. Genetics 164(4):1645–1656. 12930768 10.1093/genetics/164.4.1645 PMC1462670

[msad178-B50] Rannala B , Yang Z. 2017. Efficient Bayesian species tree inference under the multispecies coalescent. Syst Biol. 66:823–842. 28053140 10.1093/sysbio/syw119 PMC8562347

[msad178-B51] Schumer M , Xu C, Powell DL, Durvasula A, Skov L, Holland C, Blazier JC, Sankararaman S, Andolfatto P, Rosenthal GG, et al. 2018. Natural selection interacts with recombination to shape the evolution of hybrid genomes. Science 360(6389):656–660. 29674434 10.1126/science.aar3684 PMC6069607

[msad178-B52] Slotman MA , Calzetta M, Powell JR. 2005. Differential introgression of chromosomal regions between *Anopheles gambiae* and *An. arabiensis*. Am J Trop Med Hyg. 73(2):326–335. 16103599

[msad178-B53] Slotman M , Powell JR. 2005. Female sterility in hybrids between *Anopheles gambiae* and *A. arabiensis*, and the causes of Haldane’s rule. Evolution 59(5):1016–1026. 16136801

[msad178-B54] Slotman M , Torre A d., Powell JR. 2004. The genetics of inviability and male sterility in hybrids between *Anopheles gambiae* and *An. arabiensis*. Genetics 167(1):275–287. 15166154 10.1534/genetics.167.1.275 PMC1470845

[msad178-B55] Solis-Lemus C , Ane C. 2016. Inferring phylogenetic networks with maximum pseudolikelihood under incomplete lineage sorting. PLoS Genet. 12(3):e1005896. 26950302 10.1371/journal.pgen.1005896 PMC4780787

[msad178-B56] Tavaré S . 1984. Lines of descent and genealogical processes, and their applications in population genetics models. Theor Popul Biol. 26:119–164. 6505980 10.1016/0040-5809(84)90027-3

[msad178-B57] Thawornwattana Y , Dalquen D, Yang Z. 2018. Coalescent analysis of phylogenomic data confidently resolves the species relationships in the *Anopheles gambiae* species complex. Mol Biol Evol. 35(10):2512–2527. 30102363 10.1093/molbev/msy158 PMC6188554

[msad178-B58] Thawornwattana Y , Seixas FA, Mallet J, Yang Z. 2022. Full-likelihood genomic analysis clarifies a complex history of species divergence and introgression: the example of the *erato-sara* group of *Heliconius* butterflies. Syst Biol. 71(5):1159–1177. 35169847 10.1093/sysbio/syac009 PMC9366460

[msad178-B59] Tiley GP , Flouri T, Jiao X, Poelstra JP, Xu B, Zhu T, Rannala B, Yoder AD, Yang Z. 2023. Estimation of species divergence times in presence of cross-species gene flow. Syst Biol. 72(4):820–836. 36961245 10.1093/sysbio/syad015 PMC10405360

[msad178-B60] Wakeley J . 2009. Coalescent theory: an introduction. Greenwood Village (CO): Roberts and Company.

[msad178-B61] Wen D , Nakhleh L. 2018. Coestimating reticulate phylogenies and gene trees from multilocus sequence data. Syst Biol. 67(3):439–457. 29088409 10.1093/sysbio/syx085

[msad178-B62] Yang Z . 2014. Molecular evolution: a statistical approach. Oxford (UK): Oxford University Press.

[msad178-B63] Yang Z . 2015. The BPP program for species tree estimation and species delimitation. Curr Zool. 61:854–865.

[msad178-B64] Yang Z , Flouri T. 2022. Estimation of cross-species introgression rates using genomic data despite model unidentifiability. Mol Biol Evol. 39(5):msac083. 35417543 10.1093/molbev/msac083 PMC9087891

[msad178-B65] Yang Z , Rannala B. 2014. Unguided species delimitation using DNA sequence data from multiple loci. Mol Biol Evol. 31(12):3125–3135. 25274273 10.1093/molbev/msu279 PMC4245825

[msad178-B66] Yang Z , Zhu T. 2018. Bayesian selection of misspecified models is overconfident and may cause spurious posterior probabilities for phylogenetic trees. Proc Natl Acad Sci U S A. 115(8):1854–1859. 29432193 10.1073/pnas.1712673115 PMC5828583

[msad178-B67] Yu Y , Degnan JH, Nakhleh L. 2012. The probability of a gene tree topology within a phylogenetic network with applications to hybridization detection. PLoS Genet. 8(4):e1002660. 22536161 10.1371/journal.pgen.1002660 PMC3330115

[msad178-B68] Yu Y , Dong J, Liu KJ, Nakhleh L. 2014. Maximum likelihood inference of reticulate evolutionary histories. Proc Natl Acad Sci U S A. 111(46):16448–16453. 25368173 10.1073/pnas.1407950111 PMC4246314

[msad178-B69] Zhang C , Ogilvie HA, Drummond AJ, Stadler T. 2018. Bayesian inference of species networks from multilocus sequence data. Mol Biol Evol. 35:504–517. 29220490 10.1093/molbev/msx307 PMC5850812

[msad178-B70] Zhu T , Flouri T, Yang Z. 2022. A simulation study to examine the impact of recombination on phylogenomic inferences under the multispecies coalescent model. Mol Ecol. 31:2814–2829. 35313033 10.1111/mec.16433 PMC9321900

