# Supplementary material for: Inferring the Direction of Introgression Using Genomic Sequence Data
Source: Mol Biol Evol. 2023 Aug 8;40(8):msad178. doi: 10.1093/molbev/msad178 (PMC10439365; doi:10.1093/molbev/msad178)
Supplement: msad178_Supplementary_Data [file msad178_supplementary_data.pdf]

Supplemental Information for
Inferring the direction of introgression using genomic sequence data

Yuttapong Thawornwattana, Jun Huang, Tomáš Flouri, James Mallet, and Ziheng Yang

SI text: Simulation results in the case of four species

We simulated datasets under the three MSC-I models of figure 5 for four species on the species tree $((A,(B,C)),D)$, with introgression between non-sister species A and B in different directions: inflow (I), outflow (O), and bidirectional introgression (B). The data were analyzed under the same three models (I, O, B), resulting in nine combinations.

Four species, equal population sizes

Figure S4 shows average posterior means and 95% HPD CIs in analysis of data simulated assuming the same population size ($\theta_0 = \theta_1 = 0.01$). The results for the large datasets of $L = 4000$ are summarized in table S2. Parameters shared among all three models (I, O, B) are well estimated, with no discernible impact of model misspecification. These include species divergence and introgression times (τ_R, τ_S, τ_T, and $\tau_X = \tau_Y$), population sizes for the extant species ($\theta_A, \theta_B, \theta_C, \theta_D$) and for ancestral species not involved in gene flow (θ_R, θ_S). As discussed in the case of two species, introgression time is well estimated even if the introgression direction is misspecified (e.g., τ_X in the I-O and O-I settings), because the estimate is dominated by the minimum sequence divergence between species involved in introgression (t_{ab}) (table 1). Ancestral population sizes (θ_R, θ_S) are slightly less well estimated but appear to converge to the correct values in all settings when the number of loci $L \to \infty$ (fig. S4). Below we focus on introgression probabilities (φ_X, φ_Y) and population sizes θ_X, θ_Y, and θ_T.

In the I-I, O-O, and B-B settings, the true model is assumed in the analysis, and the results provide a reference for comparison. The introgression probability φ_Y in the I-I setting is more precisely estimated than φ_X in the O-O setting, with narrower CIs. Inflow is easier to infer than outflow, as observed in our simulations for the three-species case (fig. 4**b**&**c**, same θ). Similarly in the B-B setting, the inflow probability φ_Y is better estimated than the outflow probability φ_X. The B-B setting had slightly wider CIs for φ_X and φ_Y than in the I-I and O-O settings, due to more parameters in model B (fig. S4, table S2). Overall the introgression probabilities are well estimated under all three settings, although thousands of loci appear necessary to obtain precise estimates. Population size θ_X is better estimated in the I-I setting than in the O-O and B-B settings, because estimation is affected by uncertainties in φ_X in model O and B. Similarly θ_Y is better estimated in O-O than in I-I and B-B settings.

In the I-B and O-B settings, gene flow is unidirectional but the model assumes bidirectional gene flow. The model B is over-parametrized but not misspecified. As Bayesian estimation under the correct model is consistent, the introgression probability for the nonexistent introgression (φ_X in I-B, φ_Y in O-B) should converge to 0 when the data size approaches ∞. Results in both the I-B and O-B settings are consistent with this expectation (fig. S4). Other parameters are well-estimated, with CI widths indistinguishable from those in the I-I and O-O settings. Over-parametrization in model B incurs little cost to the statistical performance of the method, as in the case of two species (fig. 3).

In the I-O and O-I settings, introgression is assumed to occur in the wrong (opposite) direction. According to our analysis of the two-species case (table 1), this misspecification should only affect estimation of the introgression probability and population sizes θ_X, θ_Y, and θ_T, while other parameters including introgression time should be correctly estimated. This is indeed the case (fig. S4). In the I-O setting, we expect θ_X to be underestimated, θ_Y and θ_T to be overestimated, and the introgression probability φ_X may be larger or smaller than φ_Y depending on how the estimates of θ_Y and θ_T compare with the true value of θ_X (table 1). Simulation results confirm these predictions (fig. S4). In the O-I setting, the effects are the opposite: θ_X was overestimated while θ_Y and θ_T were underestimated. As expected, φ_Y was estimated to be smaller than φ_X.

Finally, in the B-O and B-I settings, introgression occurs in both directions but is assumed to occur in only one direction. The estimates of θ_X, θ_Y, and θ_T follow the same pattern as in I-O and O-I, respectively. The introgression probability (φ_X in B-O, φ_Y in B-I) is larger and less well-estimated than when the true model has unidirectional gene flow (φ_X in O-I, φ_Y in I-O). This positive bias may be explained by the fact that gene flow in the two directions in the true model B have an accumulative effect on the distribution of the coalescent times between species (t_{ab}) (see eq. A4). For instance, in the B-I setting, $B \to A$ introgression in the true model B is expected to increase the chance of coalescence during $\tau_X < t_{ab} < \tau_S$, and such introgression events may be recognized and misinterpreted as extra $A \to B$ introgression in the fitting model I, leading to $\hat{\varphi}_Y^{(\mathrm{I})} > \varphi_Y^{(\mathrm{B})}$.

Four species, different population sizes

Results for cases with different population sizes are summarized in figure S5 and table S3. In our setting, model I assumes inflow from a small population to a large one, model O assumes outflow from a large population to a small one, while model B assumes both inflow from small population to large one as well as outflow from large to small (fig. 5).

As in the case of equal population sizes, species

divergence and introgression times (τ_R, τ_S, τ_T, and $\tau_X = \tau_Y$) and population sizes for extant species ($\theta_A, \theta_B, \theta_C, \theta_D$) and for common ancestors R and S (θ_R, θ_S) are all well estimated, in spite of model misspecification. Thus we focus on introgression probabilities (φ_X, φ_Y) and population sizes θ_X, θ_Y, and θ_T.

In the I-I, O-O, and B-B settings, the correct model is assumed in the analysis. Introgression probability φ_Y in model I is far more precisely estimated than φ_X in model O. At $L = 4000$ loci, the average 95% CI is 0.19-0.22 for I-I and 0.16-0.24 for O-O (table S3). The difference is far greater than in the case of equal population sizes where the inflow probability φ_Y in model I is slightly better estimated than the outflow probability φ_X in model O (table S2). It is easier to estimate the introgression probability from a small population to a large one (model I) than in the opposite direction, as discussed before. Similarly in the B-B setting, the inflow probability of small → large introgression (φ_Y) is much better estimated than the outflow probability of large → small introgression (φ_X): for $L = 4000$, the average 95% CIs are 0.18-0.23 for φ_Y and 0.15-0.24 for φ_X (table S3). In the I-B and O-B settings, model B is over-parameterized. Performance is very similar to that in the I-I and O-O settings, respectively, with φ for the nonexistent migration approaching 0 with the increase of data size (fig. S5, table S3).

In the I-O and O-I settings, the introgression direction is misspecified. In the I-O setting, $\hat{\varphi}_X$ is much greater than in the case of equal population sizes. The extremely large $\hat{\varphi}_X$ mimics the extreme estimate in the two-species case (fig. 3**c** small→large, model O). In the O-I setting, gene flow is from a large population into a small one, and the donor population size θ_Y is grossly underestimated while the recipient population size θ_X is overestimated when introgression direction is misspecified. These patterns are similar to those in the two-species analysis (fig. 3**d** large→small, model O). The estimate $\hat{\varphi}_Y$ is much lower than the true introgression probability $\varphi_X = 0.2$ in the opposite direction.

The B-I and B-O settings show a cumulative effect in the estimates of the migration rate: $\hat{\varphi}_Y$ is greater in the B-I setting than in the I-I setting, and $\hat{\varphi}_X$ is greater in the B-O setting than in the O-O setting. This is the same pattern as found in the case of equal population sizes (fig. S4).

Bayesian test of introgression

We apply the Bayesian test of introgression (Ji *et al.* (2023)) to the data analyzed in figures S4&S5, with results summarized in figures S6&S7. In the I-I, O-O, and B-O settings, where the correct model is assumed, the power of the test is high, reaching ~ 100% at $L \geq 1000$ loci (figs. S6&S7). In the I-B and O-B settings, the power of detecting introgression in the direction that exists in the true model (B) is high, while the false positive rate for detecting non-existent introgression in the incorrect direction is low, below the nominal 1%. In the I-O and O-I settings, where introgression direction is misspecified, the false positive rate is very high, comparable to the power in the analysis under the correct model. Overall, the results are similar to those for the two-species simulations (fig. S2).

References

Ji, J., Jackson, D. J., Leache, A. D., and Yang, Z. 2023. Power of Bayesian and heuristic tests to detect cross-species introgression with reference to gene flow in the *Tamias quadrivittatus* group of North American chipmunks. *Syst. Biol.*, 72(2): 446–465.

Tavaré, S. 1984. Lines of descent and genealogical processes, and their applications in population genetics models. *Theor. Popul. Biol.*, 26: 119–164.

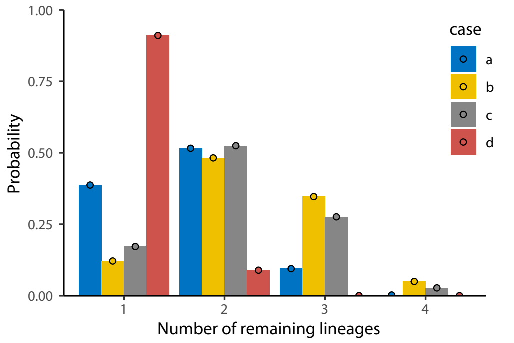

Figure S1: Probability distribution of the number of lineages remaining at time T (in $2N$ generations) when we trace, backwards in time, the genealogy for a sample of n sequences randomly sampled from a diploid population of size N. The four cases are for $n = 4$ and $T = 1, 0.5, 0.6,$ and 3, for cases **a**–**d** of figure 3. The bars represent estimates from 10^7 simulations while circles are from eqs. 6.1 & 6.2 in Tavaré (1984).

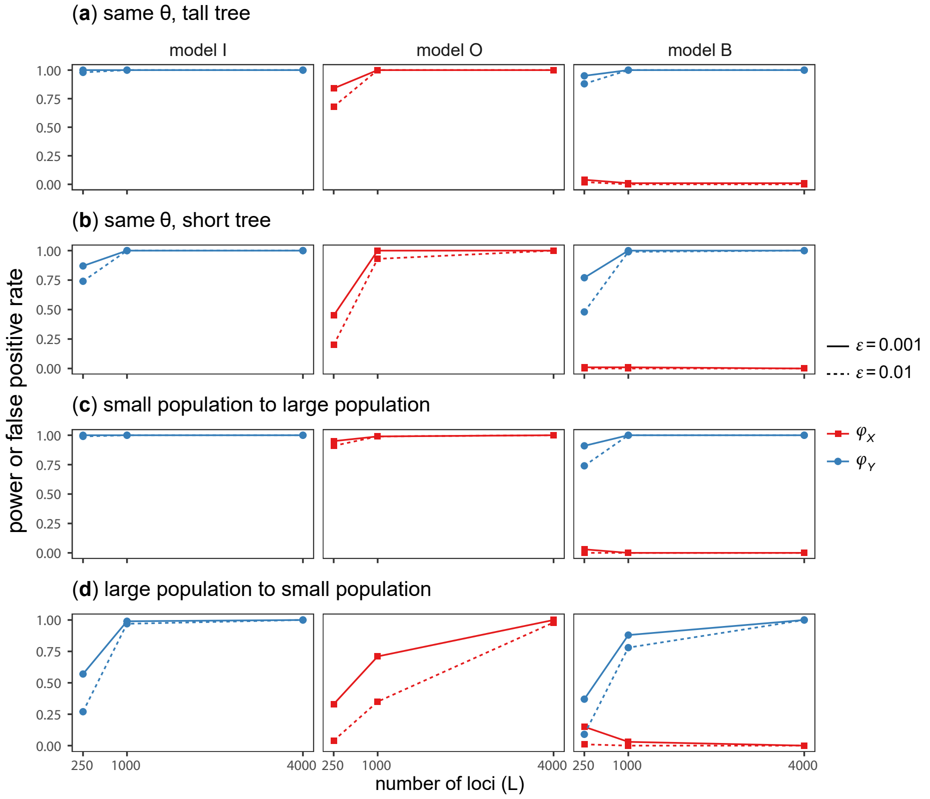

Figure S2: (**2s-power**) Power (blue) and false positive rate (red) of Bayesian test for introgression applied to the simulated data with two species under model I of figure 1**a** using four sets of parameter values (cases **a**–**d**). Bayesian test is conducted using a cut-off of 100 for the Bayes factor, calculated using the Savage-Dickey density ratio with the small value for null effect ($\epsilon = 0.001$ or 0.01). Note that model I is the true model with $A \to B$ introgression with probability φ_Y. A significant result for testing the null H_0: $\varphi_Y = 0$ under model I or B is considered a true positive, whereas a significant result for testing the null H_0: $\varphi_X = 0$ under model O or B is considered a false positive. Parameter estimates from those data are summarized in figure 3.

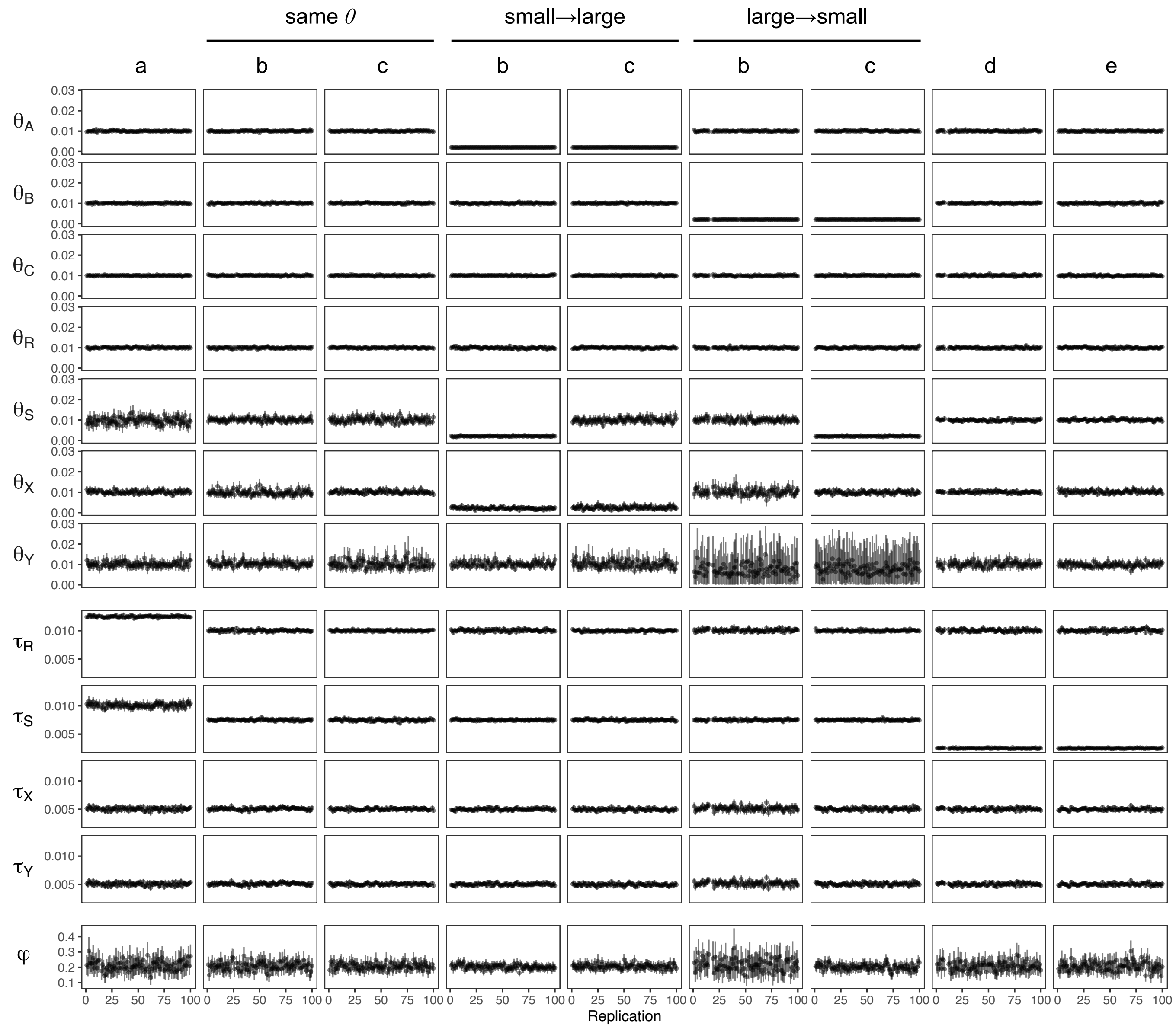

Figure S3: Posterior means and 95% HPD CIs for parameters in 100 replicate datasets simulated and analyzed under the models of figure 4**a**–**e**. Results for φ are shown also in figure 4**h**.

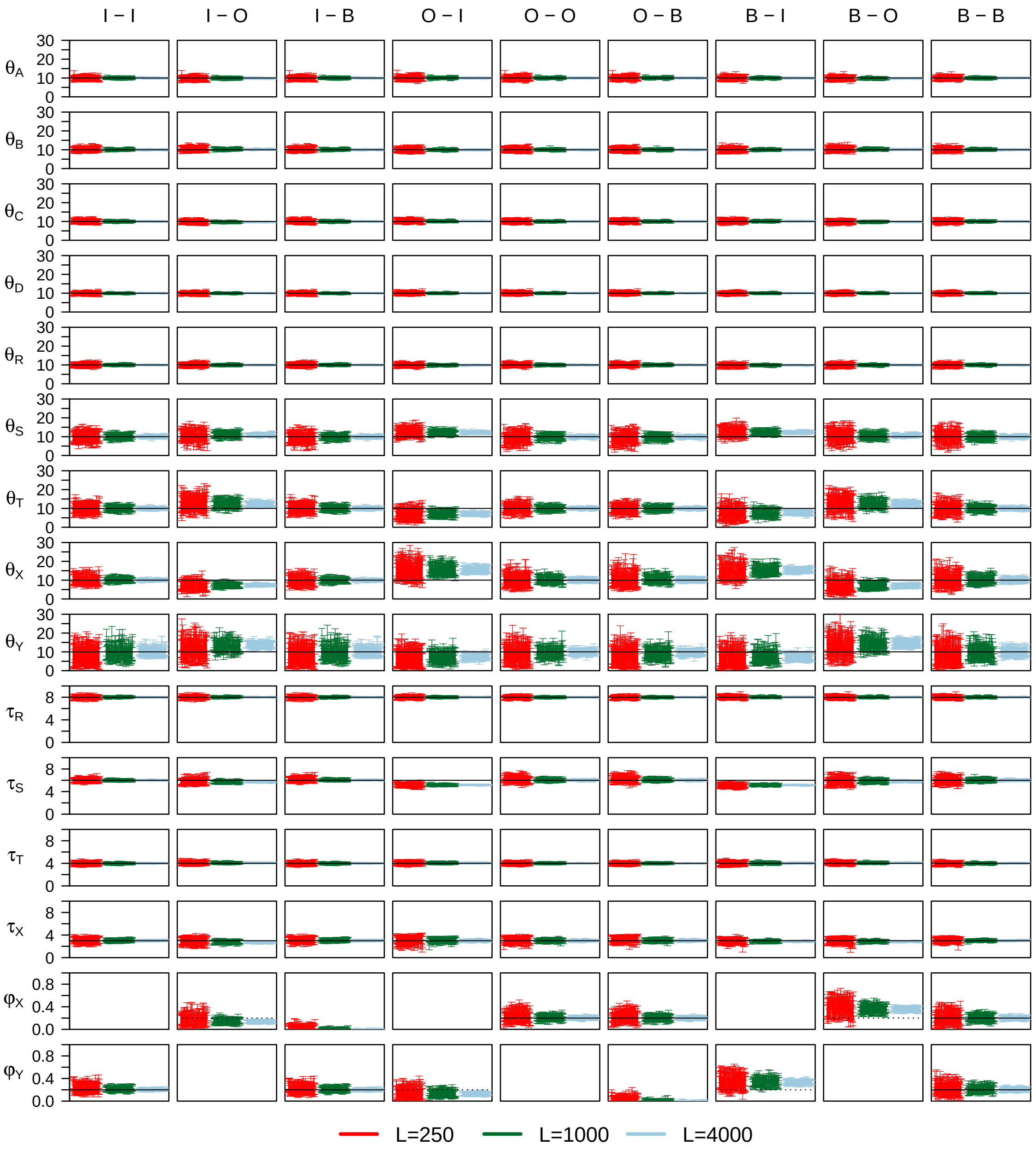

Figure S4: **(4s-same-θ)** The 95% HPD CIs for parameters in 100 replicate datasets simulated and analyzed under models I, O, and B of figure 5, with all species on the species tree having the same population size ($\theta_0 = \theta_1 = 0.01$) when the data are generated. The nine settings are specified in the simulation-analysis format; i.e., 'I-O' means that data were simulated under model I and analyzed under model O. Parameters θ and τ are multiplied by 10^3. Horizontal solid lines indicate the true values. In the I-O setting, the dotted line for φ_X in model O indicates the true value of φ_Y in model I assumed in the simulation, while in the O-I setting, the dotted line for φ_Y indicates the true value of φ_X in the assumed O model. In the B-I and B-O settings, two introgression probabilities exist in the simulation model (φ_X, φ_Y) but only one is assumed in the analysis model, and the dotted line indicates its true value.

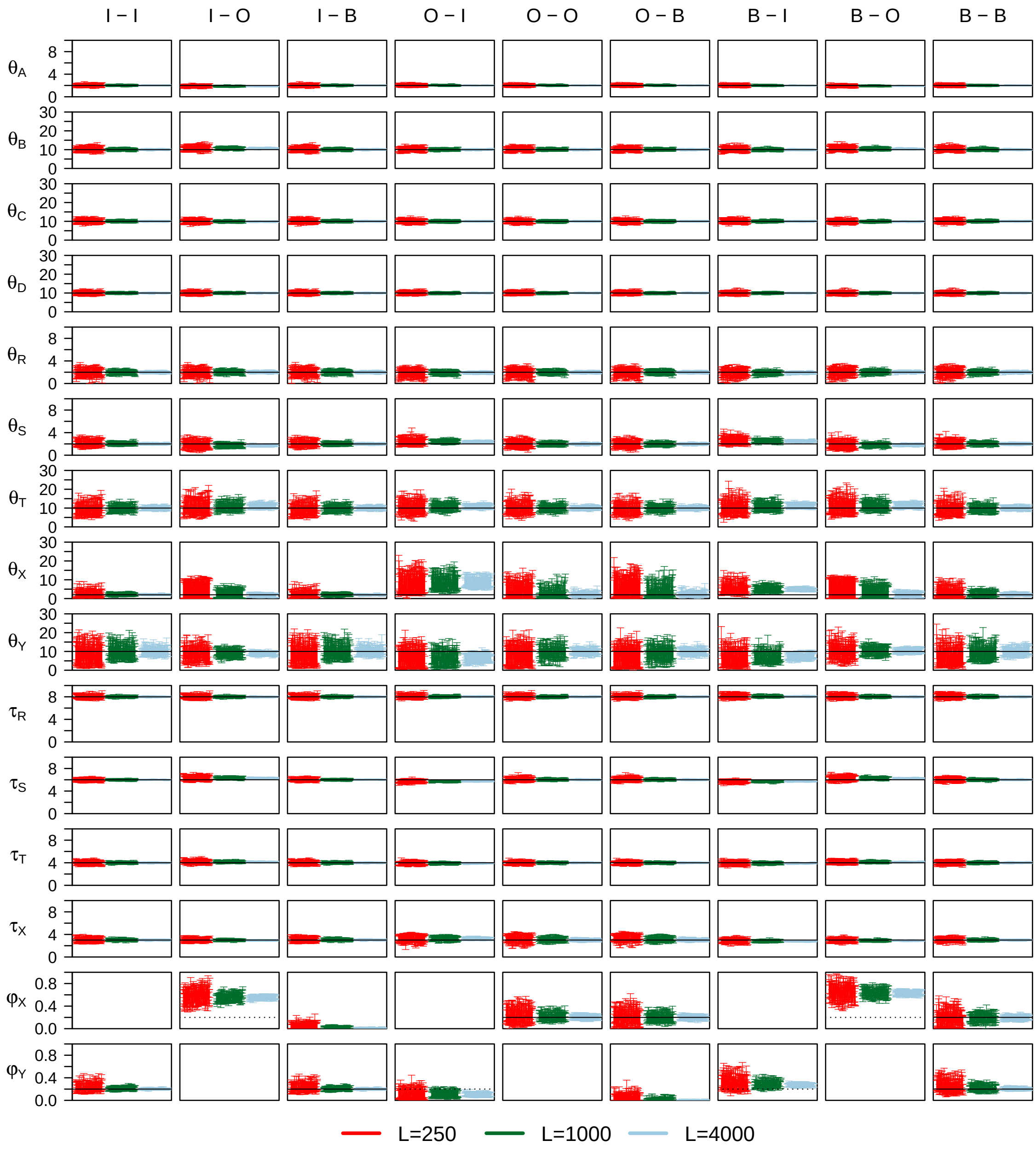

Figure S5: **(4s-diff-θ)** The 95% HPD CIs for parameters in 100 replicate datasets simulated and analyzed under models I, O, and B of figure 5, with θ_0 = 0.002 for the thin branches and θ_1 = 0.01 for the thick branches in the species tree. Other details as in figure S4.

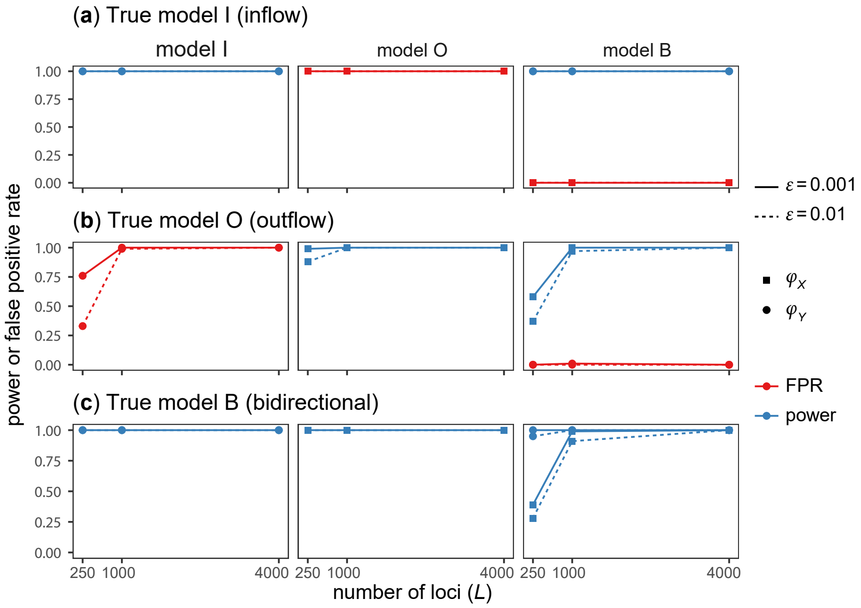

Figure S6: (**4s-same-θ-power**) Power (blue) and false positive rate (red) of Bayesian test for introgression applied to data of four species simulated under the (**a**) inflow (I), (**b**) outflow (O), and (**c**) bidirectional (B) models of figure 5**a**–**c**, assuming the same θ for all populations. The data were analyzed under the same I, O, and B models, resulting in nine combinations. Parameter estimates from those data are summarized in figure S4.

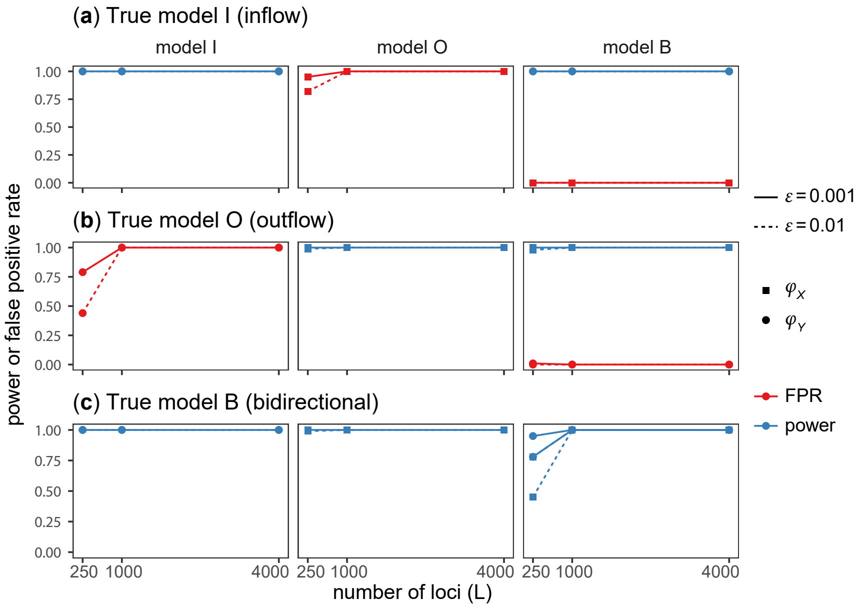

Figure S7: (**4s-diff-θ-power**) Power (blue) and false positive rate (red) of Bayesian test for introgression applied to data of four species simulated under the I, O, and B models of figure 5**a**–**c**, assuming different θ for populations on the species tree. Parameter estimates from those data are summarized in figure S5. Other details as in figure S6.

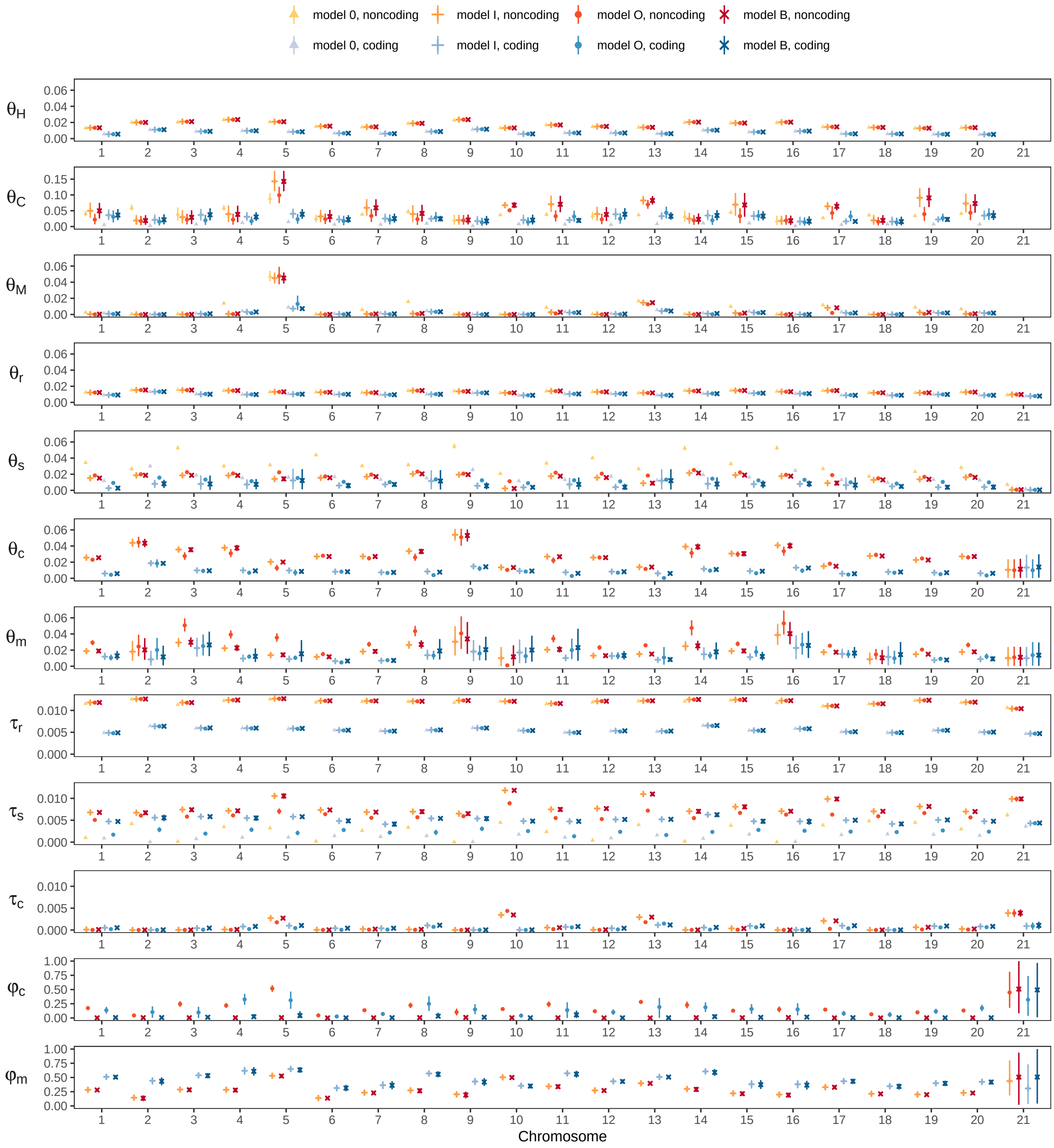

Figure S8: Posterior means and 95% HPD CIs for parameters in BPP analyses of coding and noncoding data from the different chromosomes of *H. cydno* (C), *H. melpomene* (M) and *H. hecale* (*H*) (fig. 5). The four models are (Ø) MSC with no gene flow, (I) MSC-I with $C \to M$ introgression, (O) MSC-I with $M \to C$ introgression, and (B) MSC-I with $C \leftrightarrows M$ bidirectional introgression (see table 2). Results for chromosome 1 are also shown in table 2. In model Ø with no gene flow, branches C and c are assigned the same θ_C, and branches M and m are assigned the same θ_M (fig. 6). For the sex chromosome (chr 21), there is only one sequence per species per locus, so that the population sizes for extant species ($\theta_C, \theta_M, \theta_H$) are unidentifiable/inestimable in all four models. Furthermore, θ_m in model I and θ_c in model O are unidentifiable, and models I and O are unidentifiable (with the parameter mapping $\varphi_m^{(\mathrm{I})} = \varphi_c^{(\mathrm{O})}$ and $\theta_c^{(\mathrm{I})} = \theta_m^{(\mathrm{O})}$). See Discussion for a discussion of unidentifiability issues for data of chromosome 21 (the X chromosome).

Table S1. (2s-4 cases) Average posterior means and 95% HPD CIs for parameters over 100 replicate datasets of L = 4000 loci simulated under model I (inflow) and analyzed under model I (inflow), model O (outflow) and model B (bidirectional) (fig. 1) using four sets of parameter values (cases a–d)

	(**a**) Same θ tall tree				(**b**) Same θ short tree				(**c**) Small to large				(**d**) Large to small			
	Θ_I	$\hat{\Theta}_I$	$\hat{\Theta}_O$	$\hat{\Theta}_B$	Θ_I	$\hat{\Theta}_I$	$\hat{\Theta}_O$	$\hat{\Theta}_B$	Θ_I	$\hat{\Theta}_I$	$\hat{\Theta}_O$	$\hat{\Theta}_B$	Θ_I	$\hat{\Theta}_I$	$\hat{\Theta}_O$	$\hat{\Theta}_B$
θ_A	1.0	1.00 (0.97, 1.03)	1.00 (0.97, 1.03)	1.00 (0.97, 1.03)	1.00	1.01 (0.97, 1.04)	1.01 (0.97, 1.04)	1.01 (0.97, 1.04)	0.2	0.20 (0.19, 0.21)	0.19 (0.19, 0.20)	0.20 (0.19, 0.21)	1.0	1.00 (0.97, 1.03)	1.00 (0.97, 1.03)	1.00 (0.97, 1.03)
θ_B	1.0	1.00 (0.97, 1.03)	1.00 (0.97, 1.03)	1.00 (0.97, 1.03)	1.00	1.00 (0.97, 1.04)	1.00 (0.97, 1.04)	1.00 (0.97, 1.04)	1.0	1.00 (0.97, 1.03)	1.02 (0.99, 1.05)	1.00 (0.97, 1.03)	0.2	0.20 (0.19, 0.21)	0.20 (0.19, 0.21)	0.20 (0.19, 0.21)
θ_R	1.0	1.00 (0.96, 1.04)	1.06 (1.02, 1.10)	1.00 (0.95, 1.04)	1.00	1.00 (0.97, 1.03)	1.02 (0.99, 1.05)	1.00 (0.97, 1.03)	0.2	0.20 (0.16, 0.23)	0.14 (0.09, 0.19)	0.19 (0.16, 0.23)	1.0	1.00 (0.96, 1.04)	1.02 (0.98, 1.05)	1.00 (0.96, 1.04)
θ_X	1.0	1.01 (0.94, 1.08)	0.45 (0.33, 0.58)	0.98 (0.90, 1.06)	1.00	1.00 (0.93, 1.08)	0.44 (0.24, 0.65)	0.97 (0.88, 1.05)	0.2	0.20 (0.16, 0.25)	0.50 (0.01, 1.19)	0.18 (0.11, 0.24)	1.0	1.00 (0.93, 1.08)	0.65 (0.40, 0.88)	0.99 (0.91, 1.07)
θ_Y	1.0	1.00 (0.88, 1.12)	1.91 (1.69, 2.14)	1.02 (0.90, 1.15)	1.00	1.00 (0.85, 1.14)	1.84 (1.56, 2.14)	1.02 (0.87, 1.17)	1.0	0.98 (0.81, 1.15)	1.00 (0.88, 1.12)	1.01 (0.83, 1.19)	0.2	0.21 (0.09, 0.34)	1.07 (0.56, 1.65)	0.24 (0.10, 0.39)
τ_R	1.0	1.00 (0.97, 1.03)	0.93 (0.90, 0.95)	1.00 (0.98, 1.03)	0.50	0.50 (0.48, 0.52)	0.47 (0.45, 0.49)	0.50 (0.48, 0.52)	0.6	0.60 (0.58, 0.63)	0.69 (0.65, 0.73)	0.60 (0.58, 0.63)	0.6	0.60 (0.57, 0.63)	0.57 (0.54, 0.59)	0.61 (0.57, 0.64)
τ_X	0.5	0.50 (0.47, 0.53)	0.55 (0.52, 0.57)	0.50 (0.47, 0.53)	0.25	0.25 (0.23, 0.28)	0.28 (0.25, 0.31)	0.25 (0.23, 0.28)	0.3	0.30 (0.28, 0.32)	0.36 (0.34, 0.37)	0.31 (0.29, 0.33)	0.3	0.30 (0.27, 0.34)	0.35 (0.31, 0.38)	0.31 (0.27, 0.34)
φ_X	n/a	n/a	0.27 (0.20, 0.33)	0.01 (0.00, 0.02)	n/a	n/a	0.30 (0.18, 0.44)	0.01 (0.00, 0.04)	n/a	n/a	0.98 (0.96, 1.00)	0.02 (0.00, 0.05)	n/a	n/a	0.17 (0.06, 0.31)	0.01 (0.00, 0.02)
φ_Y	0.2	0.20 (0.17, 0.24)	n/a	0.19 (0.16, 0.23)	0.20	0.21 (0.15, 0.27)	n/a	0.20 (0.14, 0.26)	0.2	0.21 (0.17, 0.25)	n/a	0.20 (0.16, 0.25)	0.2	0.21 (0.13, 0.29)	n/a	0.20 (0.12, 0.29)

Note.— Θ_I denotes the true parameter values, while $\hat{\Theta}_I$, $\hat{\Theta}_O$, and $\hat{\Theta}_B$ are estimates under models I, O and B, respectively (fig. 1). There are n = 4 sequences per species per locus and N = 500 sites in the sequence. Values of τ and θ are multiplied by 100. Estimates for individual datasets and for all data sizes (L = 250, 1000, 4000) are plotted in figure 3.

Table S2. (4s-same-θ) Average posterior means and 95% HPD CIs for parameters over 100 replicate datasets of L = 4000 loci simulated and analyzed under the inflow (I), outflow (O) and bidirectional (B) models of figure 5 with the same population size (θ) for all species

	True model I				True model O				True model B			
	Θ_I	$\hat{\Theta}_I$	$\hat{\Theta}_O$	$\hat{\Theta}_B$	Θ_O	$\hat{\Theta}_I$	$\hat{\Theta}_O$	$\hat{\Theta}_B$	Θ_B	$\hat{\Theta}_I$	$\hat{\Theta}_O$	$\hat{\Theta}_B$
θ_A	1.0	1.00 (0.97, 1.03)	0.98 (0.95, 1.02)	1.00 (0.97, 1.03)	1.0	1.00 (0.97, 1.03)	1.00 (0.97, 1.03)	1.00 (0.97, 1.03)	1.0	1.00 (0.96, 1.03)	0.98 (0.95, 1.01)	1.00 (0.97, 1.03)
θ_B	1.0	1.00 (0.97, 1.03)	1.02 (0.99, 1.06)	1.00 (0.97, 1.03)	1.0	0.99 (0.96, 1.03)	1.00 (0.97, 1.03)	1.00 (0.97, 1.03)	1.0	0.99 (0.96, 1.03)	1.02 (0.98, 1.05)	1.00 (0.97, 1.03)
θ_C	1.0	1.00 (0.97, 1.03)	0.97 (0.95, 1.00)	1.00 (0.97, 1.03)	1.0	1.02 (0.99, 1.05)	1.00 (0.97, 1.03)	1.00 (0.97, 1.03)	1.0	1.01 (0.98, 1.04)	0.98 (0.95, 1.00)	1.00 (0.97, 1.03)
θ_D	1.0	1.00 (0.98, 1.02)	1.00 (0.98, 1.02)	1.00 (0.98, 1.02)	1.0	1.00 (0.98, 1.02)	1.00 (0.98, 1.02)	1.00 (0.98, 1.02)	1.0	1.00 (0.98, 1.02)	1.00 (0.98, 1.02)	1.00 (0.98, 1.02)
θ_R	1.0	1.00 (0.97, 1.03)	0.99 (0.96, 1.02)	1.00 (0.97, 1.03)	1.0	0.98 (0.96, 1.01)	1.00 (0.97, 1.03)	1.00 (0.97, 1.03)	1.0	0.98 (0.95, 1.01)	0.99 (0.96, 1.02)	1.00 (0.97, 1.02)
θ_S	1.0	1.00 (0.93, 1.08)	1.11 (1.03, 1.19)	0.99 (0.92, 1.07)	1.0	1.22 (1.16, 1.29)	0.99 (0.91, 1.07)	0.99 (0.91, 1.07)	1.0	1.23 (1.17, 1.30)	1.07 (0.99, 1.16)	1.00 (0.91, 1.08)
θ_T	1.0	1.00 (0.92, 1.08)	1.24 (1.12, 1.36)	1.00 (0.92, 1.08)	1.0	0.71 (0.62, 0.81)	1.00 (0.93, 1.08)	1.00 (0.92, 1.07)	1.0	0.77 (0.66, 0.88)	1.26 (1.15, 1.38)	1.00 (0.91, 1.08)
θ_X	1.0	1.00 (0.94, 1.07)	0.74 (0.68, 0.80)	0.99 (0.92, 1.06)	1.0	1.55 (1.39, 1.72)	1.00 (0.90, 1.11)	1.01 (0.91, 1.12)	1.0	1.52 (1.39, 1.66)	0.69 (0.59, 0.79)	1.01 (0.88, 1.13)
θ_Y	1.0	1.00 (0.77, 1.26)	1.36 (1.19, 1.54)	1.01 (0.77, 1.28)	1.0	0.74 (0.58, 0.91)	0.98 (0.82, 1.14)	0.96 (0.80, 1.12)	1.0	0.70 (0.52, 0.90)	1.44 (1.26, 1.63)	0.98 (0.76, 1.21)
τ_R	8.0	0.80 (0.79, 0.81)	0.80 (0.79, 0.81)	0.80 (0.79, 0.81)	8.0	0.80 (0.79, 0.81)	0.80 (0.79, 0.81)	0.80 (0.79, 0.81)	8.0	0.80 (0.80, 0.81)	0.80 (0.79, 0.81)	0.80 (0.79, 0.81)
τ_S	6.0	0.60 (0.59, 0.61)	0.57 (0.56, 0.58)	0.60 (0.59, 0.61)	6.0	0.52 (0.51, 0.53)	0.60 (0.59, 0.62)	0.60 (0.59, 0.62)	6.0	0.52 (0.51, 0.52)	0.58 (0.56, 0.60)	0.60 (0.58, 0.62)
τ_T	4.0	0.40 (0.39, 0.41)	0.41 (0.40, 0.42)	0.40 (0.39, 0.41)	4.0	0.41 (0.40, 0.42)	0.40 (0.39, 0.41)	0.40 (0.39, 0.41)	4.0	0.40 (0.39, 0.41)	0.41 (0.40, 0.42)	0.40 (0.39, 0.41)
τ_X	3.0	0.30 (0.29, 0.32)	0.27 (0.26, 0.29)	0.30 (0.29, 0.32)	3.0	0.30 (0.28, 0.32)	0.30 (0.28, 0.32)	0.30 (0.29, 0.32)	3.0	0.29 (0.28, 0.30)	0.28 (0.27, 0.30)	0.30 (0.29, 0.31)
φ_X	n/a	n/a	0.13 (0.11, 0.16)	0.01 (0.00, 0.01)	0.2	n/a	0.20 (0.17, 0.23)	0.20 (0.17, 0.23)	0.2	n/a	0.36 (0.32, 0.40)	0.20 (0.17, 0.23)
φ_Y	0.2	0.20 (0.18, 0.23)	n/a	0.20 (0.18, 0.22)	n/a	0.13 (0.10, 0.16)	n/a	0.01 (0.00, 0.01)	0.2	0.33 (0.29, 0.37)	n/a	0.21 (0.17, 0.24)

Note.— Θ_I, Θ_O, and Θ_B denote the true parameter values in the true model, while $\hat{\Theta}_I$, $\hat{\Theta}_O$ and $\hat{\Theta}_B$ are estimates (fig. 5). Each dataset consists of L = 4000 loci, with n = 4 sequences per species per locus and N = 500 sites in the sequence. Values of τ and θ are multiplied by 100. Results for all data sizes with L = 250, 1000 or 4000 loci are shown in figure S4.

Table S3. (4s-diff-θ) Average posterior means and 95% HPD CIs for parameters over 100 replicate datasets of L = 4000 loci simulated and analyzed under the I, O, and B models of figure 5 with different population sizes

	Model I				Model O				Model B			
	Θ_I	$\hat{\Theta}_I$	$\hat{\Theta}_O$	$\hat{\Theta}_B$	Θ_O	$\hat{\Theta}_I$	$\hat{\Theta}_O$	$\hat{\Theta}_B$	Θ_B	$\hat{\Theta}_I$	$\hat{\Theta}_O$	$\hat{\Theta}_B$
θ_A	0.2	0.20 (0.19, 0.21)	0.19 (0.18, 0.19)	0.20 (0.19, 0.21)	0.2	0.20 (0.19, 0.21)	0.20 (0.19, 0.21)	0.20 (0.19, 0.21)	0.2	0.20 (0.19, 0.20)	0.19 (0.18, 0.20)	0.20 (0.19, 0.21)
θ_B	1.0	1.00 (0.97, 1.03)	1.06 (1.03, 1.10)	1.00 (0.97, 1.03)	1.0	1.00 (0.97, 1.03)	1.00 (0.97, 1.03)	1.00 (0.97, 1.03)	1.0	1.00 (0.96, 1.03)	1.04 (1.01, 1.07)	1.00 (0.97, 1.03)
θ_C	1.0	1.00 (0.97, 1.03)	0.99 (0.96, 1.01)	1.00 (0.97, 1.03)	1.0	1.00 (0.97, 1.03)	1.00 (0.97, 1.03)	1.00 (0.97, 1.03)	1.0	1.00 (0.97, 1.03)	0.99 (0.96, 1.01)	1.00 (0.97, 1.03)
θ_D	1.0	1.00 (0.97, 1.02)	1.00 (0.97, 1.02)	1.00 (0.97, 1.02)	1.0	1.00 (0.97, 1.02)	1.00 (0.98, 1.02)	1.00 (0.98, 1.02)	1.0	1.00 (0.97, 1.02)	1.00 (0.97, 1.02)	1.00 (0.97, 1.02)
θ_R	0.2	0.20 (0.18, 0.22)	0.20 (0.19, 0.22)	0.20 (0.18, 0.22)	0.2	0.19 (0.18, 0.21)	0.20 (0.18, 0.22)	0.20 (0.18, 0.22)	0.2	0.19 (0.17, 0.21)	0.20 (0.18, 0.22)	0.20 (0.18, 0.22)
θ_S	0.2	0.20 (0.19, 0.21)	0.17 (0.16, 0.19)	0.20 (0.18, 0.21)	0.2	0.24 (0.22, 0.25)	0.20 (0.18, 0.22)	0.20 (0.18, 0.22)	0.2	0.25 (0.23, 0.26)	0.18 (0.16, 0.20)	0.20 (0.18, 0.22)
θ_T	1.0	1.00 (0.91, 1.09)	1.11 (0.99, 1.23)	0.99 (0.90, 1.08)	1.0	1.08 (0.97, 1.19)	1.00 (0.91, 1.10)	1.00 (0.90, 1.09)	1.0	1.12 (1.00, 1.25)	1.14 (1.01, 1.27)	1.00 (0.90, 1.11)
θ_X	0.2	0.20 (0.17, 0.24)	0.17 (0.07, 0.28)	0.19 (0.16, 0.23)	0.2	0.85 (0.59, 1.14)	0.22 (0.11, 0.33)	0.24 (0.12, 0.37)	0.2	0.48 (0.41, 0.56)	0.20 (0.05, 0.36)	0.22 (0.15, 0.29)
θ_Y	1.0	0.99 (0.78, 1.23)	0.89 (0.78, 1.00)	1.01 (0.78, 1.25)	1.0	0.64 (0.41, 0.86)	0.99 (0.82, 1.17)	0.98 (0.80, 1.16)	1.0	0.71 (0.56, 0.86)	1.03 (0.91, 1.15)	0.96 (0.75, 1.19)
τ_R	8.0	0.80 (0.79, 0.81)	0.80 (0.79, 0.81)	0.80 (0.79, 0.81)	8.0	0.80 (0.79, 0.81)	0.80 (0.79, 0.81)	0.80 (0.79, 0.81)	8.0	0.81 (0.80, 0.82)	0.80 (0.79, 0.81)	0.80 (0.79, 0.81)
τ_S	6.0	0.60 (0.59, 0.61)	0.63 (0.62, 0.64)	0.60 (0.59, 0.61)	6.0	0.57 (0.56, 0.58)	0.60 (0.59, 0.61)	0.60 (0.59, 0.61)	6.0	0.57 (0.56, 0.58)	0.62 (0.61, 0.63)	0.60 (0.59, 0.61)
τ_T	4.0	0.40 (0.39, 0.41)	0.41 (0.40, 0.42)	0.40 (0.39, 0.41)	4.0	0.39 (0.38, 0.40)	0.40 (0.39, 0.41)	0.40 (0.39, 0.41)	4.0	0.39 (0.38, 0.40)	0.41 (0.40, 0.42)	0.40 (0.39, 0.41)
τ_X	3.0	0.30 (0.29, 0.31)	0.30 (0.29, 0.30)	0.30 (0.29, 0.31)	3.0	0.33 (0.31, 0.35)	0.30 (0.28, 0.32)	0.30 (0.28, 0.33)	3.0	0.28 (0.27, 0.29)	0.29 (0.28, 0.30)	0.30 (0.29, 0.31)
φ_X	n/a	n/a	0.55 (0.51, 0.59)	0.01 (0.00, 0.02)	0.2	n/a	0.20 (0.16, 0.24)	0.20 (0.15, 0.24)	0.2	n/a	0.63 (0.59, 0.67)	0.19 (0.15, 0.24)
φ_Y	0.2	0.20 (0.19, 0.22)	n/a	0.20 (0.18, 0.22)	n/a	0.10 (0.07, 0.14)	n/a	0.00 (0.00, 0.01)	0.2	0.27 (0.24, 0.30)	n/a	0.20 (0.18, 0.23)

Note.— Results for all data sizes with L = 250, 1000 or 4000 loci are in figure S5. See legend to table S2.

Table S4. Numbers of coding and noncoding loci on each chromosome from the genomic data of *Heliconius* butterflies (fig. 6)

	Number of loci			Number of loci	
Chr	Coding	Noncoding	Chr	Coding	Noncoding
1	4,942	5,341	12	5,242	4,962
2	2,902	2,534	13	4,201	5,361
3	2,349	3,113	14	2,480	2,629
4	1,998	2,839	15	2,719	2,920
5	2,316	2,901	16	2,126	3,002
6	3,922	4,167	17	4,380	4,390
7	3,717	4,301	18	3,601	5,068
8	2,877	2,706	19	4,519	4,929
9	1,957	2,554	20	4,659	4,505
10	5,172	5,540	21	3,078	3,275
11	4,074	3,541			

Table S5. Posterior means and 95% HPD CIs for parameters in analyses of the coding and noncoding data on each on each chromosome from Heliconius under four models (I, O, B, 0)

region	variable	chr	model 0	model I	model O	model B
noncoding	theta_H	1	0.01313 (0.01268, 0.01357)	0.01339 (0.01295, 0.01386)	0.01337 (0.01292, 0.01383)	0.01339 (0.01294, 0.01385)
noncoding	theta_H	2	0.01994 (0.01886, 0.02106)	0.02003 (0.01892, 0.02112)	0.02003 (0.01893, 0.02114)	0.02003 (0.01894, 0.02115)
noncoding	theta_H	3	0.02039 (0.01940, 0.02141)	0.02106 (0.02004, 0.02212)	0.02102 (0.01998, 0.02205)	0.02107 (0.02001, 0.02208)
noncoding	theta_H	4	0.02330 (0.02206, 0.02452)	0.02351 (0.02227, 0.02476)	0.02349 (0.02226, 0.02474)	0.02351 (0.02229, 0.02476)
noncoding	theta_H	5	0.02068 (0.01964, 0.02172)	0.02091 (0.01985, 0.02195)	0.02088 (0.01985, 0.02196)	0.02091 (0.01985, 0.02196)
noncoding	theta_H	6	0.01500 (0.01439, 0.01559)	0.01541 (0.01480, 0.01603)	0.01541 (0.01480, 0.01603)	0.01541 (0.01479, 0.01603)
noncoding	theta_H	7	0.01432 (0.01377, 0.01488)	0.01449 (0.01394, 0.01506)	0.01447 (0.01392, 0.01503)	0.01449 (0.01392, 0.01504)
noncoding	theta_H	8	0.01878 (0.01779, 0.01974)	0.01893 (0.01794, 0.01992)	0.01891 (0.01793, 0.01989)	0.01893 (0.01796, 0.01993)
noncoding	theta_H	9	0.02261 (0.02134, 0.02389)	0.02352 (0.02222, 0.02487)	0.02351 (0.02220, 0.02485)	0.02352 (0.02222, 0.02488)
noncoding	theta_H	10	0.01319 (0.01275, 0.01363)	0.01320 (0.01276, 0.01364)	0.01321 (0.01277, 0.01366)	0.01320 (0.01275, 0.01364)
noncoding	theta_H	11	0.01671 (0.01594, 0.01745)	0.01691 (0.01614, 0.01767)	0.01688 (0.01613, 0.01766)	0.01691 (0.01615, 0.01767)
noncoding	theta_H	12	0.01478 (0.01424, 0.01531)	0.01509 (0.01455, 0.01565)	0.01507 (0.01453, 0.01563)	0.01509 (0.01455, 0.01565)
noncoding	theta_H	13	0.01390 (0.01341, 0.01436)	0.01396 (0.01349, 0.01444)	0.01399 (0.01350, 0.01446)	0.01396 (0.01348, 0.01444)
noncoding	theta_H	14	0.01986 (0.01878, 0.02089)	0.02047 (0.01939, 0.02157)	0.02042 (0.01934, 0.02152)	0.02047 (0.01940, 0.02157)
noncoding	theta_H	15	0.01912 (0.01817, 0.02008)	0.01930 (0.01833, 0.02027)	0.01928 (0.01831, 0.02025)	0.01930 (0.01832, 0.02026)
noncoding	theta_H	16	0.01965 (0.01869, 0.02064)	0.02040 (0.01939, 0.02142)	0.02038 (0.01938, 0.02142)	0.02040 (0.01936, 0.02140)
noncoding	theta_H	17	0.01446 (0.01389, 0.01502)	0.01452 (0.01395, 0.01508)	0.01454 (0.01396, 0.01510)	0.01452 (0.01394, 0.01508)
noncoding	theta_H	18	0.01384 (0.01336, 0.01433)	0.01388 (0.01338, 0.01437)	0.01387 (0.01339, 0.01436)	0.01388 (0.01339, 0.01436)
noncoding	theta_H	19	0.01283 (0.01237, 0.01327)	0.01291 (0.01245, 0.01336)	0.01290 (0.01243, 0.01334)	0.01291 (0.01245, 0.01337)
noncoding	theta_H	20	0.01349 (0.01299, 0.01400)	0.01363 (0.01311, 0.01414)	0.01361 (0.01309, 0.01411)	0.01363 (0.01312, 0.01415)
noncoding	theta_H	21	n/a	n/a	n/a	n/a
noncoding	theta_C	1	0.04072 (0.03288, 0.04964)	0.04990 (0.02739, 0.07593)	0.02212 (0.00676, 0.04017)	0.04970 (0.02577, 0.07497)
noncoding	theta_C	2	0.05921 (0.04970, 0.06946)	0.01919 (0.00517, 0.03656)	0.01726 (0.00406, 0.03363)	0.01894 (0.00526, 0.03586)
noncoding	theta_C	3	0.03868 (0.01875, 0.06060)	0.02972 (0.00976, 0.05223)	0.02195 (0.00648, 0.04031)	0.02907 (0.00787, 0.05161)
noncoding	theta_C	4	0.05852 (0.04968, 0.06785)	0.03962 (0.01404, 0.06652)	0.02189 (0.00586, 0.04082)	0.03841 (0.01352, 0.06606)
noncoding	theta_C	5	0.08858 (0.07180, 0.10625)	0.14309 (0.11183, 0.17617)	0.09934 (0.07394, 0.12586)	0.14284 (0.11160, 0.17613)
noncoding	theta_C	6	0.02472 (0.01393, 0.03756)	0.03194 (0.01343, 0.05303)	0.02333 (0.00774, 0.04092)	0.03154 (0.01308, 0.05263)
noncoding	theta_C	7	0.03862 (0.03394, 0.04330)	0.05934 (0.03429, 0.08696)	0.03368 (0.01433, 0.05538)	0.05917 (0.03332, 0.08634)
noncoding	theta_C	8	0.04724 (0.04051, 0.05454)	0.03956 (0.01398, 0.06763)	0.02310 (0.00501, 0.04300)	0.04148 (0.01587, 0.06882)
noncoding	theta_C	9	0.02027 (0.00546, 0.03780)	0.02014 (0.00589, 0.03724)	0.01961 (0.00578, 0.03648)	0.02062 (0.00668, 0.03780)
noncoding	theta_C	10	0.03674 (0.03386, 0.03966)	0.06767 (0.05861, 0.07738)	0.05131 (0.04586, 0.05687)	0.06767 (0.05832, 0.07728)
noncoding	theta_C	11	0.03843 (0.03302, 0.04414)	0.07060 (0.04612, 0.09807)	0.03271 (0.01600, 0.05133)	0.07039 (0.04528, 0.09742)
noncoding	theta_C	12	0.03379 (0.02286, 0.04622)	0.03948 (0.01885, 0.06258)	0.02267 (0.00737, 0.04078)	0.03805 (0.01769, 0.06180)
noncoding	theta_C	13	0.03666 (0.03364, 0.03974)	0.08261 (0.06932, 0.09652)	0.06986 (0.05609, 0.08414)	0.08250 (0.06965, 0.09671)
noncoding	theta_C	14	0.03206 (0.01521, 0.05068)	0.02477 (0.00849, 0.04387)	0.01923 (0.00510, 0.03626)	0.02269 (0.00702, 0.04132)
noncoding	theta_C	15	0.04507 (0.03923, 0.05116)	0.06957 (0.03427, 0.10619)	0.03299 (0.01071, 0.05760)	0.06823 (0.03085, 0.10622)
noncoding	theta_C	16	0.01781 (0.00436, 0.03419)	0.01805 (0.00482, 0.03437)	0.01923 (0.00557, 0.03662)	0.01830 (0.00511, 0.03454)
noncoding	theta_C	17	0.02722 (0.02508, 0.02942)	0.06394 (0.05126, 0.07772)	0.04281 (0.02229, 0.06455)	0.06394 (0.05117, 0.07785)
noncoding	theta_C	18	0.03580 (0.03304, 0.03867)	0.01954 (0.00537, 0.03673)	0.01528 (0.00336, 0.03018)	0.01936 (0.00570, 0.03613)
noncoding	theta_C	19	0.03424 (0.03154, 0.03699)	0.09091 (0.06148, 0.12124)	0.03922 (0.01747, 0.06245)	0.09059 (0.06150, 0.12248)
noncoding	theta_C	20	0.04083 (0.03619, 0.04567)	0.07281 (0.04470, 0.10424)	0.04361 (0.01992, 0.06980)	0.07294 (0.04368, 0.10231)
noncoding	theta_C	21	n/a	n/a	n/a	n/a
noncoding	theta_M	1	0.00257 (0.00208, 0.00307)	0.00034 (0.00019, 0.00048)	9e-05 (3e-05, 0.00017)	0.00033 (0.00017, 0.00049)
noncoding	theta_M	2	0.00048 (0.00043, 0.00053)	1e-05 (0.00000, 1e-05)	0.00000 (0.00000, 1e-05)	1e-05 (0.00000, 1e-05)
noncoding	theta_M	3	0.00043 (2e-04, 0.00066)	0.00013 (1e-05, 0.00023)	8e-05 (2e-05, 0.00016)	0.00012 (1e-05, 0.00023)
noncoding	theta_M	4	0.01388 (0.01275, 0.01505)	0.00094 (0.00017, 0.00186)	0.00044 (4e-05, 0.00092)	0.00088 (0.00012, 0.00180)
noncoding	theta_M	5	0.04738 (0.04113, 0.05406)	0.04521 (0.03834, 0.05215)	0.04774 (0.03742, 0.05905)	0.04518 (0.03839, 0.05212)
noncoding	theta_M	6	0.00017 (0.00012, 0.00021)	4e-05 (2e-05, 7e-05)	3e-05 (1e-05, 5e-05)	4e-05 (2e-05, 7e-05)
noncoding	theta_M	7	0.00573 (0.00529, 0.00617)	0.00073 (0.00043, 0.00105)	0.00032 (0.00012, 0.00052)	0.00072 (0.00042, 0.00102)
noncoding	theta_M	8	0.01577 (0.01432, 0.01726)	0.00116 (0.00014, 0.00240)	0.00048 (3e-05, 0.00110)	0.00127 (0.00029, 0.00250)
noncoding	theta_M	9	1e-05 (0.00000, 2e-05)	1e-05 (0.00000, 1e-05)	1e-05 (0.00000, 1e-05)	1e-05 (0.00000, 1e-05)
noncoding	theta_M	10	4e-05 (3e-05, 5e-05)	4e-05 (4e-05, 5e-05)	4e-05 (4e-05, 5e-05)	4e-05 (4e-05, 5e-05)
noncoding	theta_M	11	0.00855 (0.00774, 0.00935)	0.00280 (0.00197, 0.00363)	0.00123 (6e-04, 0.00186)	0.00279 (0.00196, 0.00363)
noncoding	theta_M	12	0.00064 (0.00049, 0.00078)	0.00012 (6e-05, 0.00018)	5e-05 (2e-05, 9e-05)	0.00011 (4e-05, 0.00017)
noncoding	theta_M	13	0.01665 (0.01574, 0.01759)	0.01462 (0.01366, 0.01556)	0.01261 (0.01127, 0.01395)	0.01462 (0.01367, 0.01557)
noncoding	theta_M	14	5e-04 (0.00024, 0.00076)	0.00011 (4e-05, 0.00021)	8e-05 (2e-05, 0.00017)	9e-05 (2e-05, 0.00016)
noncoding	theta_M	15	0.00983 (0.00910, 0.01059)	0.00177 (5e-04, 0.00298)	0.00044 (4e-05, 0.00089)	0.00172 (0.00042, 0.00309)
noncoding	theta_M	16	1e-05 (0.00000, 3e-05)	1e-05 (0.00000, 1e-05)	1e-05 (0.00000, 2e-05)	1e-05 (0.00000, 1e-05)
noncoding	theta_M	17	0.01166 (0.01098, 0.01233)	0.00825 (0.00733, 0.00918)	0.00192 (0.00071, 0.00305)	0.00826 (0.00732, 0.00918)
noncoding	theta_M	18	8e-05 (6e-05, 9e-05)	0.00000 (0.00000, 1e-05)	0.00000 (0.00000, 0.00000)	0.00000 (0.00000, 1e-05)
noncoding	theta_M	19	0.00894 (0.00852, 0.00937)	0.00256 (0.00168, 0.00344)	0.00065 (0.00021, 0.00108)	0.00254 (0.00167, 0.00348)
noncoding	theta_M	20	0.00672 (0.00628, 0.00717)	0.00093 (0.00056, 0.00130)	0.00042 (9e-05, 0.00066)	0.00093 (0.00058, 0.00129)
noncoding	theta_M	21	n/a	n/a	n/a	n/a
noncoding	theta_r	1	0.01237 (0.01192, 0.01282)	0.01228 (0.01181, 0.01274)	0.01222 (0.01176, 0.01268)	0.01228 (0.01181, 0.01274)
noncoding	theta_r	2	0.01530 (0.01449, 0.01614)	0.01539 (0.01455, 0.01623)	0.01535 (0.01450, 0.01618)	0.01538 (0.01456, 0.01623)
noncoding	theta_r	3	0.01562 (0.01498, 0.01624)	0.01526 (0.01461, 0.01590)	0.01522 (0.01456, 0.01585)	0.01526 (0.01462, 0.01591)
noncoding	theta_r	4	0.01464 (0.01397, 0.01530)	0.01470 (0.01401, 0.01536)	0.01465 (0.01398, 0.01532)	0.01469 (0.01402, 0.01537)
noncoding	theta_r	5	0.01304 (0.01242, 0.01366)	0.01309 (0.01245, 0.01372)	0.01302 (0.01240, 0.01365)	0.01309 (0.01246, 0.01374)
noncoding	theta_r	6	0.01276 (0.01223, 0.01329)	0.01256 (0.01201, 0.01310)	0.01253 (0.01199, 0.01308)	0.01256 (0.01200, 0.01310)
noncoding	theta_r	7	0.01215 (0.01164, 0.01265)	0.01209 (0.01158, 0.01262)	0.01206 (0.01154, 0.01257)	0.01208 (0.01156, 0.01260)

noncoding	theta_r	8	0.01458 (0.01389, 0.01528)	0.01466 (0.01394, 0.01536)	0.01462 (0.01391, 0.01533)	0.01466 (0.01395, 0.01538)
noncoding	theta_r	9	0.01416 (0.01344, 0.01486)	0.01375 (0.01304, 0.01448)	0.01373 (0.01300, 0.01444)	0.01375 (0.01302, 0.01447)
noncoding	theta_r	10	0.01172 (0.01125, 0.01216)	0.01189 (0.01143, 0.01235)	0.01185 (0.01139, 0.01232)	0.01189 (0.01144, 0.01236)
noncoding	theta_r	11	0.01403 (0.01342, 0.01463)	0.01411 (0.01347, 0.01472)	0.01402 (0.01340, 0.01463)	0.01410 (0.01347, 0.01472)
noncoding	theta_r	12	0.01310 (0.01260, 0.01360)	0.01306 (0.01253, 0.01357)	0.01297 (0.01246, 0.01348)	0.01305 (0.01252, 0.01356)
noncoding	theta_r	13	0.01173 (0.01130, 0.01215)	0.01183 (0.01139, 0.01227)	0.01173 (0.01131, 0.01218)	0.01183 (0.01138, 0.01227)
noncoding	theta_r	14	0.01463 (0.01391, 0.01535)	0.01425 (0.01350, 0.01498)	0.01423 (0.01350, 0.01495)	0.01425 (0.01352, 0.01499)
noncoding	theta_r	15	0.01473 (0.01406, 0.01540)	0.01476 (0.01406, 0.01544)	0.01473 (0.01407, 0.01544)	0.01476 (0.01407, 0.01544)
noncoding	theta_r	16	0.01370 (0.01309, 0.01430)	0.01327 (0.01266, 0.01390)	0.01324 (0.01263, 0.01386)	0.01327 (0.01263, 0.01387)
noncoding	theta_r	17	0.01449 (0.01396, 0.01503)	0.01468 (0.01412, 0.01521)	0.01459 (0.01404, 0.01512)	0.01468 (0.01413, 0.01523)
noncoding	theta_r	18	0.01185 (0.01138, 0.01231)	0.01192 (0.01145, 0.01239)	0.01190 (0.01143, 0.01236)	0.01192 (0.01145, 0.01239)
noncoding	theta_r	19	0.01184 (0.01136, 0.01231)	0.01192 (0.01142, 0.01240)	0.01187 (0.01140, 0.01236)	0.01192 (0.01143, 0.01241)
noncoding	theta_r	20	0.01271 (0.01221, 0.01321)	0.01278 (0.01225, 0.01329)	0.01274 (0.01223, 0.01326)	0.01278 (0.01226, 0.01330)
noncoding	theta_r	21	0.00924 (0.00856, 0.00989)	0.00981 (0.00913, 0.01048)	0.00981 (0.00914, 0.01048)	0.00979 (0.00912, 0.01046)
noncoding	theta_s	1	0.03431 (0.03283, 0.03578)	0.01517 (0.01412, 0.01624)	0.01847 (0.01751, 0.01943)	0.01518 (0.01413, 0.01627)
noncoding	theta_s	2	0.02672 (0.02433, 0.02913)	0.01865 (0.01680, 0.02046)	0.01996 (0.01817, 0.02177)	0.01866 (0.01683, 0.02051)
noncoding	theta_s	3	0.05265 (0.05046, 0.05499)	0.01863 (0.01678, 0.02046)	0.02258 (0.02075, 0.02429)	0.01867 (0.01683, 0.02051)
noncoding	theta_s	4	0.03005 (0.02822, 0.03188)	0.01844 (0.01678, 0.02014)	0.02098 (0.01938, 0.02266)	0.01847 (0.01677, 0.02014)
noncoding	theta_s	5	0.03150 (0.02981, 0.03315)	0.01420 (0.01131, 0.01708)	0.02236 (0.02027, 0.02445)	0.01423 (0.01136, 0.01713)
noncoding	theta_s	6	0.04387 (0.04213, 0.04559)	0.01560 (0.01436, 0.01686)	0.01727 (0.01606, 0.01848)	0.01560 (0.01437, 0.01688)
noncoding	theta_s	7	0.03024 (0.02876, 0.03175)	0.01702 (0.01580, 0.01825)	0.01958 (0.01844, 0.02076)	0.01703 (0.01579, 0.01824)
noncoding	theta_s	8	0.03154 (0.02957, 0.03362)	0.02040 (0.01839, 0.02238)	0.02325 (0.02143, 0.02506)	0.02040 (0.01837, 0.02241)
noncoding	theta_s	9	0.05469 (0.05175, 0.05773)	0.01949 (0.01760, 0.02139)	0.02074 (0.01888, 0.02262)	0.01948 (0.01758, 0.02133)
noncoding	theta_s	10	0.02071 (0.01968, 0.02173)	0.00239 (0.00036, 0.00455)	0.01126 (0.00961, 0.01286)	0.00231 (0.00033, 0.00446)
noncoding	theta_s	11	0.03362 (0.03177, 0.03547)	0.01754 (0.01563, 0.01937)	0.02189 (0.02023, 0.02358)	0.01757 (0.01570, 0.01944)
noncoding	theta_s	12	0.04022 (0.03886, 0.04162)	0.01579 (0.01443, 0.01715)	0.02070 (0.01946, 0.02192)	0.01582 (0.01445, 0.01718)
noncoding	theta_s	13	0.02653 (0.02546, 0.02764)	0.00887 (0.00641, 0.01127)	0.01836 (0.01720, 0.01957)	0.00890 (0.00643, 0.01125)
noncoding	theta_s	14	0.05227 (0.04978, 0.05477)	0.02144 (0.01926, 0.02356)	0.02514 (0.02303, 0.02720)	0.02150 (0.01936, 0.02368)
noncoding	theta_s	15	0.03304 (0.03093, 0.03522)	0.01889 (0.01683, 0.02103)	0.02222 (0.02043, 0.02407)	0.01892 (0.01684, 0.02100)
noncoding	theta_s	16	0.05265 (0.05016, 0.05515)	0.01758 (0.01593, 0.01920)	0.01924 (0.01763, 0.02090)	0.01759 (0.01592, 0.01921)
noncoding	theta_s	17	0.02682 (0.02550, 0.02821)	0.00901 (0.00595, 0.01200)	0.01890 (0.01755, 0.02027)	0.00903 (0.00594, 0.01200)
noncoding	theta_s	18	0.01740 (0.01648, 0.01833)	0.01294 (0.01195, 0.01391)	0.01488 (0.01390, 0.01585)	0.01294 (0.01196, 0.01391)
noncoding	theta_s	19	0.02314 (0.02206, 0.02424)	0.01375 (0.01263, 0.01491)	0.01667 (0.01570, 0.01768)	0.01372 (0.01259, 0.01487)
noncoding	theta_s	20	0.02828 (0.02687, 0.02966)	0.01610 (0.01488, 0.01730)	0.01879 (0.01765, 0.01988)	0.01609 (0.01489, 0.01731)
noncoding	theta_s	21	0.00710 (0.00582, 0.00840)	0.00079 (0.00016, 0.00172)	0.00079 (8e-05, 0.00167)	8e-04 (0.00011, 0.00161)
noncoding	theta_c	1	n/a	0.00592 (0.00553, 0.00632)	0.00438 (0.00324, 0.00548)	0.00577 (0.00528, 0.00624)
noncoding	theta_c	2	n/a	0.01875 (0.01714, 0.02040)	0.01842 (0.01329, 0.02355)	0.01848 (0.01675, 0.02026)
noncoding	theta_c	3	n/a	0.00980 (0.00892, 0.01069)	0.00918 (0.00644, 0.01181)	0.00938 (0.00817, 0.01052)
noncoding	theta_c	4	n/a	0.00984 (0.00871, 0.01100)	0.00687 (0.00452, 0.00919)	0.00925 (0.00750, 0.01087)
noncoding	theta_c	5	n/a	0.00966 (0.00875, 0.01057)	0.00702 (0.00331, 0.01108)	0.00850 (0.00620, 0.01025)
noncoding	theta_c	6	n/a	0.00822 (0.00758, 0.00886)	0.00821 (0.00748, 0.00892)	0.00814 (0.00749, 0.00881)
noncoding	theta_c	7	n/a	0.00739 (0.00673, 0.00805)	0.00660 (0.00569, 0.00752)	0.00728 (0.00658, 0.00794)
noncoding	theta_c	8	n/a	0.00843 (0.00765, 0.00925)	0.00393 (0.00156, 0.00624)	0.00761 (0.00584, 0.00901)
noncoding	theta_c	9	n/a	0.01472 (0.01334, 0.01611)	0.01211 (0.00888, 0.01527)	0.01428 (0.01272, 0.01594)
noncoding	theta_c	10	n/a	0.00908 (0.00859, 0.00956)	0.00837 (0.00739, 0.00929)	0.00899 (0.00847, 0.00952)
noncoding	theta_c	11	n/a	0.00741 (0.00680, 0.00803)	0.00289 (0.00118, 0.00462)	0.00614 (0.00433, 0.00777)
noncoding	theta_c	12	n/a	0.00822 (0.00769, 0.00875)	0.00696 (0.00577, 0.00807)	0.00814 (0.00760, 0.00870)
noncoding	theta_c	13	n/a	0.00604 (0.00559, 0.00649)	0.00034 (4e-05, 0.00081)	0.00596 (0.00549, 0.00644)
noncoding	theta_c	14	n/a	0.01163 (0.01063, 0.01269)	0.00956 (0.00705, 0.01195)	0.01086 (0.00917, 0.01239)
noncoding	theta_c	15	n/a	0.00904 (0.00799, 0.01013)	0.00625 (0.00403, 0.00845)	0.00873 (0.00748, 0.00995)
noncoding	theta_c	16	n/a	0.01325 (0.01201, 0.01442)	0.00953 (0.00656, 0.01240)	0.01271 (0.01113, 0.01423)
noncoding	theta_c	17	n/a	0.00577 (0.00531, 0.00624)	0.00464 (0.00360, 0.00564)	0.00564 (0.00512, 0.00617)
noncoding	theta_c	18	n/a	0.00793 (0.00740, 0.00845)	0.00688 (0.00586, 0.00785)	0.00783 (0.00730, 0.00843)
noncoding	theta_c	19	n/a	0.00701 (0.00646, 0.00755)	0.00505 (0.00390, 0.00622)	0.00692 (0.00637, 0.00751)
noncoding	theta_c	20	n/a	0.00622 (0.00575, 0.00671)	0.00346 (0.00244, 0.00448)	0.00613 (0.00559, 0.00664)
noncoding	theta_c	21	n/a	0.01326 (0.00049, 0.02916)	0.00994 (2e-04, 0.02363)	0.01392 (0.00074, 0.02982)
noncoding	theta_m	1	n/a	0.01186 (0.00764, 0.01684)	0.01052 (0.00716, 0.01443)	0.01285 (0.00774, 0.01890)
noncoding	theta_m	2	n/a	0.00837 (0.00067, 0.01928)	0.02025 (0.00812, 0.03512)	0.01150 (0.00109, 0.02548)
noncoding	theta_m	3	n/a	0.02248 (0.01182, 0.03529)	0.02500 (0.01333, 0.03935)	0.02642 (0.01258, 0.04288)
noncoding	theta_m	4	n/a	0.00955 (0.00512, 0.01472)	0.01189 (0.00931, 0.01474)	0.01224 (0.00532, 0.02211)
noncoding	theta_m	5	n/a	0.00878 (0.00483, 0.01332)	0.01040 (0.00769, 0.01322)	0.01539 (0.00522, 0.03242)
noncoding	theta_m	6	n/a	0.00633 (0.00424, 0.00863)	0.00466 (0.00378, 0.00561)	0.00652 (0.00433, 0.00897)
noncoding	theta_m	7	n/a	0.00688 (0.00477, 0.00916)	0.00748 (0.00573, 0.00938)	0.00711 (0.00486, 0.00957)
noncoding	theta_m	8	n/a	0.01368 (0.00779, 0.02062)	0.01323 (0.00823, 0.01931)	0.01897 (0.00836, 0.03370)
noncoding	theta_m	9	n/a	0.01822 (0.00617, 0.03263)	0.01581 (0.00742, 0.02590)	0.02056 (0.00726, 0.03662)
noncoding	theta_m	10	n/a	0.01701 (0.00390, 0.03312)	0.01254 (0.00381, 0.02342)	0.02008 (0.00570, 0.03780)
noncoding	theta_m	11	n/a	0.01017 (0.00639, 0.01451)	0.01999 (0.00881, 0.03413)	0.02309 (0.00670, 0.04651)
noncoding	theta_m	12	n/a	0.01271 (0.00826, 0.01784)	0.01266 (0.00861, 0.01736)	0.01329 (0.00845, 0.01879)
noncoding	theta_m	13	n/a	0.00798 (0.00574, 0.01038)	0.01082 (0.00196, 0.02381)	0.00826 (0.00590, 0.01084)
noncoding	theta_m	14	n/a	0.01480 (0.00756, 0.02350)	0.01327 (0.00924, 0.01775)	0.01777 (0.00836, 0.02951)
noncoding	theta_m	15	n/a	0.01125 (0.00709, 0.01621)	0.01775 (0.01172, 0.02492)	0.01212 (0.00728, 0.01782)
noncoding	theta_m	16	n/a	0.02264 (0.00868, 0.03850)	0.02669 (0.01434, 0.04141)	0.02575 (0.01028, 0.04349)

noncoding	theta_m	17	n/a	0.01523 (0.00964, 0.02182)	0.01474 (0.01010, 0.02028)	0.01623 (0.01000, 0.02383)
noncoding	theta_m	18	n/a	0.01152 (0.00097, 0.02512)	0.00981 (0.00285, 0.01921)	0.01441 (0.00185, 0.03000)
noncoding	theta_m	19	n/a	0.00760 (0.00563, 0.00969)	0.00927 (0.00716, 0.01153)	0.00776 (0.00577, 0.00998)
noncoding	theta_m	20	n/a	0.00881 (0.00620, 0.01186)	0.01208 (0.00888, 0.01576)	0.00914 (0.00624, 0.01230)
noncoding	theta_m	21	n/a	0.01000 (0.00026, 0.02389)	0.01372 (0.00069, 0.02998)	0.01367 (0.00049, 0.02945)
noncoding	tau_r	1	0.00487 (0.00472, 0.00501)	0.00489 (0.00474, 0.00504)	0.00480 (0.00465, 0.00495)	0.00489 (0.00474, 0.00503)
noncoding	tau_r	2	0.00644 (0.00620, 0.00667)	0.00638 (0.00614, 0.00661)	0.00636 (0.00613, 0.00660)	0.00638 (0.00613, 0.00660)
noncoding	tau_r	3	0.00593 (0.00570, 0.00616)	0.00600 (0.00576, 0.00623)	0.00584 (0.00561, 0.00607)	0.00600 (0.00576, 0.00623)
noncoding	tau_r	4	0.00595 (0.00569, 0.00619)	0.00596 (0.00571, 0.00620)	0.00590 (0.00564, 0.00614)	0.00596 (0.00570, 0.00620)
noncoding	tau_r	5	0.00579 (0.00557, 0.00602)	0.00589 (0.00567, 0.00611)	0.00579 (0.00556, 0.00601)	0.00589 (0.00567, 0.00611)
noncoding	tau_r	6	0.00551 (0.00533, 0.00569)	0.00548 (0.00529, 0.00565)	0.00543 (0.00525, 0.00561)	0.00548 (0.00530, 0.00565)
noncoding	tau_r	7	0.00532 (0.00514, 0.00549)	0.00528 (0.00510, 0.00545)	0.00526 (0.00508, 0.00543)	0.00528 (0.00510, 0.00545)
noncoding	tau_r	8	0.00549 (0.00528, 0.00569)	0.00554 (0.00534, 0.00574)	0.00546 (0.00525, 0.00566)	0.00554 (0.00533, 0.00573)
noncoding	tau_r	9	0.00601 (0.00575, 0.00627)	0.00597 (0.00572, 0.00623)	0.00595 (0.00568, 0.00621)	0.00597 (0.00570, 0.00622)
noncoding	tau_r	10	0.00542 (0.00525, 0.00557)	0.00540 (0.00524, 0.00555)	0.00535 (0.00519, 0.00551)	0.00540 (0.00524, 0.00556)
noncoding	tau_r	11	0.00494 (0.00478, 0.00511)	0.00496 (0.00479, 0.00512)	0.00492 (0.00475, 0.00509)	0.00496 (0.00478, 0.00512)
noncoding	tau_r	12	0.00529 (0.00513, 0.00545)	0.00533 (0.00517, 0.00549)	0.00518 (0.00501, 0.00533)	0.00533 (0.00517, 0.00549)
noncoding	tau_r	13	0.00516 (0.00499, 0.00532)	0.00528 (0.00511, 0.00543)	0.00516 (0.00499, 0.00532)	0.00528 (0.00510, 0.00544)
noncoding	tau_r	14	0.00654 (0.00630, 0.00677)	0.00656 (0.00632, 0.00679)	0.00647 (0.00624, 0.00671)	0.00655 (0.00631, 0.00678)
noncoding	tau_r	15	0.00542 (0.00522, 0.00563)	0.00542 (0.00521, 0.00562)	0.00538 (0.00517, 0.00558)	0.00542 (0.00521, 0.00562)
noncoding	tau_r	16	0.00586 (0.00562, 0.00610)	0.00582 (0.00557, 0.00605)	0.00581 (0.00557, 0.00605)	0.00582 (0.00558, 0.00605)
noncoding	tau_r	17	0.00507 (0.00492, 0.00523)	0.00514 (0.00497, 0.00529)	0.00502 (0.00486, 0.00518)	0.00514 (0.00497, 0.00529)
noncoding	tau_r	18	0.00492 (0.00474, 0.00509)	0.00493 (0.00475, 0.00510)	0.00489 (0.00472, 0.00506)	0.00493 (0.00476, 0.00510)
noncoding	tau_r	19	0.00546 (0.00530, 0.00562)	0.00545 (0.00529, 0.00561)	0.00543 (0.00526, 0.00559)	0.00545 (0.00529, 0.00560)
noncoding	tau_r	20	0.00503 (0.00488, 0.00519)	0.00504 (0.00489, 0.00519)	0.00499 (0.00484, 0.00514)	0.00504 (0.00489, 0.00519)
noncoding	tau_r	21	0.00467 (0.00442, 0.00493)	0.00470 (0.00447, 0.00493)	0.00470 (0.00447, 0.00492)	0.00471 (0.00448, 0.00493)
noncoding	tau_s	1	0.00089 (8e-04, 0.00098)	0.00472 (0.00448, 0.00494)	0.00170 (0.00149, 0.00191)	0.00471 (0.00448, 0.00493)
noncoding	tau_s	2	6e-05 (2e-05, 1e-04)	0.00559 (0.00486, 0.00629)	0.00284 (0.00227, 0.00339)	0.00556 (0.00479, 0.00629)
noncoding	tau_s	3	0.00075 (0.00057, 0.00094)	0.00583 (0.00546, 0.00618)	0.00194 (0.00161, 0.00228)	0.00583 (0.00545, 0.00617)
noncoding	tau_s	4	0.00107 (0.00089, 0.00125)	0.00551 (0.00486, 0.00608)	0.00282 (0.00232, 0.00334)	0.00552 (0.00483, 0.00607)
noncoding	tau_s	5	0.00112 (0.00095, 0.00128)	0.00580 (0.00550, 0.00607)	0.00209 (0.00156, 0.00263)	0.00580 (0.00551, 0.00607)
noncoding	tau_s	6	0.00142 (0.00126, 0.00158)	0.00483 (0.00431, 0.00532)	0.00277 (0.00252, 0.00302)	0.00485 (0.00435, 0.00534)
noncoding	tau_s	7	0.00124 (0.00111, 0.00137)	0.00409 (0.00351, 0.00472)	0.00218 (0.00196, 0.00240)	0.00411 (0.00350, 0.00472)
noncoding	tau_s	8	0.00144 (0.00127, 0.00160)	0.00541 (0.00508, 0.00570)	0.00223 (0.00160, 0.00281)	0.00541 (0.00508, 0.00571)
noncoding	tau_s	9	7e-05 (2e-05, 0.00012)	0.00538 (0.00472, 0.00596)	0.00305 (0.00251, 0.00355)	0.00539 (0.00473, 0.00596)
noncoding	tau_s	10	0.00178 (0.00161, 0.00195)	0.00482 (0.00439, 0.00521)	0.00252 (0.00226, 0.00277)	0.00480 (0.00438, 0.00522)
noncoding	tau_s	11	0.00109 (0.00097, 0.00121)	0.00472 (0.00433, 0.00506)	0.00133 (0.00109, 0.00160)	0.00474 (0.00436, 0.00507)
noncoding	tau_s	12	0.00093 (0.00082, 0.00105)	0.00518 (0.00492, 0.00540)	0.00235 (0.00209, 0.00260)	0.00517 (0.00492, 0.00540)
noncoding	tau_s	13	0.00160 (0.00148, 0.00172)	0.00521 (0.00499, 0.00541)	0.00161 (0.00147, 0.00177)	0.00521 (0.00499, 0.00541)
noncoding	tau_s	14	0.00081 (0.00065, 0.00098)	0.00628 (0.00575, 0.00671)	0.00232 (0.00193, 0.00270)	0.00627 (0.00571, 0.00671)
noncoding	tau_s	15	0.00179 (0.00161, 0.00197)	0.00476 (0.00418, 0.00531)	0.00278 (0.00241, 0.00314)	0.00476 (0.00419, 0.00532)
noncoding	tau_s	16	1e-04 (3e-05, 0.00016)	0.00473 (0.00398, 0.00545)	0.00260 (0.00216, 0.00303)	0.00470 (0.00394, 0.00542)
noncoding	tau_s	17	0.00168 (0.00155, 0.00181)	0.00500 (0.00473, 0.00525)	0.00239 (0.00217, 0.00260)	0.00500 (0.00473, 0.00525)
noncoding	tau_s	18	0.00184 (0.00164, 0.00204)	0.00416 (0.00369, 0.00462)	0.00229 (0.00202, 0.00256)	0.00416 (0.00367, 0.00463)
noncoding	tau_s	19	0.00172 (0.00160, 0.00184)	0.00507 (0.00471, 0.00541)	0.00266 (0.00240, 0.00292)	0.00507 (0.00470, 0.00541)
noncoding	tau_s	20	0.00150 (0.00139, 0.00162)	0.00476 (0.00445, 0.00504)	0.00239 (0.00209, 0.00267)	0.00476 (0.00445, 0.00504)
noncoding	tau_s	21	0.00362 (0.00324, 0.00398)	0.00430 (0.00392, 0.00464)	0.00432 (0.00392, 0.00468)	0.00436 (0.00399, 0.00472)
noncoding	tau_c	1	n/a	0.00052 (0.00042, 0.00061)	0.00021 (8e-05, 0.00033)	0.00053 (0.00043, 0.00062)
noncoding	tau_c	2	n/a	4e-05 (3e-05, 6e-05)	2e-05 (0.00000, 4e-05)	4e-05 (2e-05, 7e-05)
noncoding	tau_c	3	n/a	0.00043 (0.00028, 0.00058)	8e-05 (0.00000, 0.00016)	0.00045 (0.00029, 6e-04)
noncoding	tau_c	4	n/a	8e-04 (0.00058, 0.00101)	0.00022 (4e-05, 0.00042)	0.00083 (6e-04, 0.00106)
noncoding	tau_c	5	n/a	0.00099 (0.00082, 0.00114)	0.00044 (0.00000, 0.00077)	0.00104 (0.00086, 0.00123)
noncoding	tau_c	6	n/a	0.00042 (0.00029, 0.00055)	6e-05 (1e-05, 0.00012)	0.00043 (3e-04, 0.00056)
noncoding	tau_c	7	n/a	4e-04 (0.00025, 0.00056)	1e-04 (2e-05, 0.00019)	0.00041 (0.00025, 0.00058)
noncoding	tau_c	8	n/a	0.00106 (0.00088, 0.00123)	0.00073 (0.00042, 0.00107)	0.00110 (0.00091, 0.00128)
noncoding	tau_c	9	n/a	3e-05 (2e-05, 5e-05)	2e-05 (0.00000, 4e-05)	4e-05 (2e-05, 7e-05)
noncoding	tau_c	10	n/a	5e-05 (2e-05, 7e-05)	1e-05 (0.00000, 3e-05)	4e-05 (3e-05, 6e-05)
noncoding	tau_c	11	n/a	0.00072 (0.00058, 0.00086)	6e-04 (0.00036, 0.00082)	0.00081 (0.00062, 0.00099)
noncoding	tau_c	12	n/a	0.00041 (3e-04, 5e-04)	0.00013 (4e-05, 0.00022)	0.00041 (0.00031, 0.00051)
noncoding	tau_c	13	n/a	0.00115 (0.00103, 0.00128)	0.00147 (0.00131, 0.00160)	0.00116 (0.00103, 0.00128)
noncoding	tau_c	14	n/a	0.00058 (0.00041, 0.00074)	8e-05 (1e-05, 0.00016)	6e-04 (0.00042, 0.00077)
noncoding	tau_c	15	n/a	0.00094 (0.00069, 0.00118)	0.00064 (0.00039, 0.00088)	0.00096 (0.00071, 0.00120)
noncoding	tau_c	16	n/a	3e-05 (1e-05, 6e-05)	2e-05 (0.00000, 4e-05)	4e-05 (2e-05, 7e-05)
noncoding	tau_c	17	n/a	0.00098 (8e-04, 0.00115)	4e-04 (0.00022, 0.00057)	0.00100 (0.00082, 0.00117)
noncoding	tau_c	18	n/a	3e-05 (1e-05, 5e-05)	1e-05 (0.00000, 3e-05)	3e-05 (1e-05, 5e-05)
noncoding	tau_c	19	n/a	0.00091 (0.00075, 0.00106)	6e-04 (4e-04, 8e-04)	0.00091 (0.00075, 0.00106)
noncoding	tau_c	20	n/a	0.00082 (0.00069, 0.00094)	0.00065 (0.00049, 0.00082)	0.00082 (0.00069, 0.00095)
noncoding	tau_c	21	n/a	9e-04 (6e-05, 0.00172)	0.00092 (6e-05, 0.00174)	0.00106 (0.00019, 0.00191)
noncoding	phi_c<-m	1	n/a	n/a	0.13595 (0.07830, 0.19593)	0.00728 (0.00000, 0.01937)
noncoding	phi_c<-m	2	n/a	n/a	0.10401 (0.00518, 0.20372)	0.00698 (0.00000, 0.02301)
noncoding	phi_c<-m	3	n/a	n/a	0.09711 (0.00000, 0.19803)	0.01299 (0.00000, 0.03940)
noncoding	phi_c<-m	4	n/a	n/a	0.32695 (0.23205, 0.42542)	0.02137 (0.00000, 0.06803)

noncoding	phi_c<-m	5	n/a	n/a	0.31102 (0.15996, 0.46466)	0.04204 (0.00000, 0.11832)
noncoding	phi_c<-m	6	n/a	n/a	0.02639 (0.01151, 0.04218)	0.00282 (0.00000, 0.00841)
noncoding	phi_c<-m	7	n/a	n/a	0.07026 (0.03709, 0.10615)	0.00466 (0.00000, 0.01384)
noncoding	phi_c<-m	8	n/a	n/a	0.24908 (0.12407, 0.38234)	0.03137 (0.00000, 0.09080)
noncoding	phi_c<-m	9	n/a	n/a	0.15048 (0.06718, 0.24245)	0.01214 (0.00000, 0.03687)
noncoding	phi_c<-m	10	n/a	n/a	0.04169 (0.00785, 0.07886)	0.00386 (0.00000, 0.01209)
noncoding	phi_c<-m	11	n/a	n/a	0.13794 (0.00000, 0.27567)	0.05520 (0.00000, 0.12933)
noncoding	phi_c<-m	12	n/a	n/a	0.10137 (0.05642, 0.15020)	0.00281 (0.00000, 0.00859)
noncoding	phi_c<-m	13	n/a	n/a	0.19210 (0.00176, 0.35197)	0.00367 (0.00000, 0.01101)
noncoding	phi_c<-m	14	n/a	n/a	0.18839 (0.10709, 0.27442)	0.02317 (0.00000, 0.06168)
noncoding	phi_c<-m	15	n/a	n/a	0.15730 (0.07160, 0.24443)	0.01095 (0.00000, 0.03343)
noncoding	phi_c<-m	16	n/a	n/a	0.15124 (0.04559, 0.25918)	0.01604 (0.00000, 0.04798)
noncoding	phi_c<-m	17	n/a	n/a	0.07873 (0.03629, 0.12391)	0.00528 (0.00000, 0.01605)
noncoding	phi_c<-m	18	n/a	n/a	0.05697 (0.01679, 0.10592)	0.00448 (0.00000, 0.01414)
noncoding	phi_c<-m	19	n/a	n/a	0.11574 (0.07158, 0.16181)	0.00370 (0.00000, 0.01106)
noncoding	phi_c<-m	20	n/a	n/a	0.17410 (0.11990, 0.23178)	0.00442 (0.00000, 0.01314)
noncoding	phi_c<-m	21	n/a	n/a	0.32184 (0.03771, 0.73888)	0.49378 (0.01240, 0.96903)
noncoding	phi_m<-c	1	n/a	0.51186 (0.47796, 0.54512)	n/a	0.50635 (0.47215, 0.54122)
noncoding	phi_m<-c	2	n/a	0.44214 (0.37537, 0.50479)	n/a	0.43340 (0.36168, 0.50215)
noncoding	phi_m<-c	3	n/a	0.53862 (0.49402, 0.58446)	n/a	0.53205 (0.48471, 0.57871)
noncoding	phi_m<-c	4	n/a	0.61804 (0.54779, 0.68624)	n/a	0.60871 (0.53344, 0.68180)
noncoding	phi_m<-c	5	n/a	0.64934 (0.60737, 0.69024)	n/a	0.63273 (0.58153, 0.68286)
noncoding	phi_m<-c	6	n/a	0.31478 (0.25730, 0.37125)	n/a	0.31434 (0.25751, 0.37057)
noncoding	phi_m<-c	7	n/a	0.36471 (0.29479, 0.43596)	n/a	0.36202 (0.29172, 0.43635)
noncoding	phi_m<-c	8	n/a	0.57075 (0.52290, 0.61786)	n/a	0.55627 (0.50236, 0.61042)
noncoding	phi_m<-c	9	n/a	0.42973 (0.36364, 0.49511)	n/a	0.42014 (0.35121, 0.48919)
noncoding	phi_m<-c	10	n/a	0.35381 (0.31389, 0.39544)	n/a	0.34922 (0.30748, 0.38952)
noncoding	phi_m<-c	11	n/a	0.57335 (0.52111, 0.62507)	n/a	0.55867 (0.49750, 0.61703)
noncoding	phi_m<-c	12	n/a	0.43229 (0.39754, 0.46689)	n/a	0.43008 (0.39587, 0.46511)
noncoding	phi_m<-c	13	n/a	0.50966 (0.47918, 0.54042)	n/a	0.50719 (0.47672, 0.53863)
noncoding	phi_m<-c	14	n/a	0.60629 (0.55528, 0.65595)	n/a	0.58939 (0.53058, 0.64735)
noncoding	phi_m<-c	15	n/a	0.38112 (0.30490, 0.45615)	n/a	0.37351 (0.29823, 0.45334)
noncoding	phi_m<-c	16	n/a	0.37992 (0.30787, 0.45231)	n/a	0.36546 (0.28624, 0.44105)
noncoding	phi_m<-c	17	n/a	0.43462 (0.38634, 0.47966)	n/a	0.43319 (0.38545, 0.47877)
noncoding	phi_m<-c	18	n/a	0.34645 (0.29424, 0.39884)	n/a	0.34238 (0.28776, 0.39390)
noncoding	phi_m<-c	19	n/a	0.39945 (0.35239, 0.44644)	n/a	0.39598 (0.34791, 0.44359)
noncoding	phi_m<-c	20	n/a	0.42318 (0.38219, 0.46390)	n/a	0.41928 (0.37683, 0.46020)
noncoding	phi_m<-c	21	n/a	0.30713 (0.03269, 0.73111)	n/a	0.50759 (0.04163, 0.99984)
coding	theta_H	1	0.00550 (0.00525, 0.00575)	0.00552 (0.00527, 0.00577)	0.00548 (0.00523, 0.00572)	0.00552 (0.00527, 0.00577)
coding	theta_H	2	0.01101 (0.01035, 0.01169)	0.01106 (0.01041, 0.01174)	0.01107 (0.01039, 0.01175)	0.01106 (0.01039, 0.01173)
coding	theta_H	3	0.00890 (0.00830, 0.00949)	0.00897 (0.00838, 0.00958)	0.00888 (0.00828, 0.00947)	0.00897 (0.00837, 0.00956)
coding	theta_H	4	0.00967 (0.00895, 0.01037)	0.00970 (0.00898, 0.01042)	0.00967 (0.00895, 0.01038)	0.00969 (0.00898, 0.01040)
coding	theta_H	5	0.00839 (0.00784, 0.00895)	0.00848 (0.00793, 0.00904)	0.00838 (0.00782, 0.00893)	0.00848 (0.00792, 0.00904)
coding	theta_H	6	0.00668 (0.00635, 0.00702)	0.00667 (0.00633, 0.00700)	0.00666 (0.00632, 0.00699)	0.00667 (0.00634, 0.00701)
coding	theta_H	7	0.00615 (0.00583, 0.00646)	0.00614 (0.00582, 0.00646)	0.00614 (0.00582, 0.00645)	0.00614 (0.00582, 0.00645)
coding	theta_H	8	0.00872 (0.00819, 0.00927)	0.00876 (0.00823, 0.00931)	0.00870 (0.00817, 0.00924)	0.00877 (0.00823, 0.00931)
coding	theta_H	9	0.01156 (0.01070, 0.01245)	0.01164 (0.01077, 0.01254)	0.01163 (0.01074, 0.01252)	0.01164 (0.01077, 0.01255)
coding	theta_H	10	0.00582 (0.00556, 0.00607)	0.00581 (0.00556, 0.00607)	0.00580 (0.00555, 0.00606)	0.00581 (0.00556, 0.00607)
coding	theta_H	11	0.00704 (0.00668, 0.00741)	0.00706 (0.00669, 0.00742)	0.00703 (0.00666, 0.00739)	0.00706 (0.00670, 0.00743)
coding	theta_H	12	0.00691 (0.00660, 0.00722)	0.00695 (0.00664, 0.00726)	0.00686 (0.00655, 0.00717)	0.00695 (0.00664, 0.00726)
coding	theta_H	13	0.00606 (0.00576, 0.00636)	0.00613 (0.00582, 0.00642)	0.00606 (0.00575, 0.00636)	0.00613 (0.00583, 0.00643)
coding	theta_H	14	0.01038 (0.00971, 0.01104)	0.01041 (0.00974, 0.01107)	0.01038 (0.00972, 0.01105)	0.01041 (0.00976, 0.01108)
coding	theta_H	15	0.00818 (0.00766, 0.00868)	0.00818 (0.00768, 0.00870)	0.00816 (0.00765, 0.00867)	0.00818 (0.00767, 0.00869)
coding	theta_H	16	0.00920 (0.00856, 0.00984)	0.00927 (0.00864, 0.00994)	0.00927 (0.00864, 0.00994)	0.00927 (0.00863, 0.00992)
coding	theta_H	17	0.00587 (0.00558, 0.00615)	0.00590 (0.00561, 0.00618)	0.00585 (0.00557, 0.00614)	0.00590 (0.00562, 0.00619)
coding	theta_H	18	0.00582 (0.00550, 0.00613)	0.00581 (0.00550, 0.00612)	0.00581 (0.00549, 0.00611)	0.00581 (0.00550, 0.00613)
coding	theta_H	19	0.00535 (0.00511, 0.00559)	0.00535 (0.00510, 0.00559)	0.00534 (0.00510, 0.00558)	0.00535 (0.00510, 0.00559)
coding	theta_H	20	0.00516 (0.00492, 0.00540)	0.00517 (0.00493, 0.00541)	0.00515 (0.00491, 0.00538)	0.00517 (0.00493, 0.00541)
coding	theta_H	21	n/a	n/a	n/a	n/a
coding	theta_C	1	0.00541 (0.00482, 0.00600)	0.03614 (0.02031, 0.05447)	0.03068 (0.01327, 0.05128)	0.03630 (0.02036, 0.05526)
coding	theta_C	2	0.00305 (0.00079, 0.00581)	0.02116 (0.00691, 0.03807)	0.01516 (0.00309, 0.03058)	0.02142 (0.00686, 0.03878)
coding	theta_C	3	0.00756 (0.00586, 0.00930)	0.03671 (0.01890, 0.05630)	0.01949 (0.00485, 0.03696)	0.03719 (0.01957, 0.05754)
coding	theta_C	4	0.01213 (0.00958, 0.01494)	0.03092 (0.01828, 0.04606)	0.01691 (0.00489, 0.03145)	0.03045 (0.01805, 0.04530)
coding	theta_C	5	0.01497 (0.01182, 0.01826)	0.04031 (0.02528, 0.05708)	0.02309 (0.00876, 0.03969)	0.03875 (0.02465, 0.05517)
coding	theta_C	6	0.00634 (0.00569, 0.00700)	0.02216 (0.01135, 0.03584)	0.01748 (0.00383, 0.03402)	0.02197 (0.01149, 0.03605)
coding	theta_C	7	0.00635 (0.00565, 0.00706)	0.02525 (0.01256, 0.04130)	0.02075 (0.00643, 0.03798)	0.02515 (0.01253, 0.04087)
coding	theta_C	8	0.00986 (0.00851, 0.01122)	0.02481 (0.01638, 0.03463)	0.02886 (0.01426, 0.04621)	0.02429 (0.01634, 0.03390)
coding	theta_C	9	0.00205 (0.00062, 0.00370)	0.01716 (0.00455, 0.03319)	0.01330 (0.00180, 0.02795)	0.01766 (0.00434, 0.03352)
coding	theta_C	10	0.00809 (0.00738, 0.00883)	0.02062 (0.00697, 0.03770)	0.01436 (0.00242, 0.02924)	0.02036 (0.00651, 0.03744)
coding	theta_C	11	0.00701 (0.00616, 0.00788)	0.02062 (0.01286, 0.03017)	0.03166 (0.01524, 0.05040)	0.01905 (0.01218, 0.02759)
coding	theta_C	12	0.00604 (0.00534, 0.00674)	0.03882 (0.02167, 0.05822)	0.02481 (0.00907, 0.04254)	0.03882 (0.02216, 0.05821)
coding	theta_C	13	0.00865 (0.00780, 0.00954)	0.03276 (0.02183, 0.04579)	0.04362 (0.02573, 0.06305)	0.03267 (0.02159, 0.04549)

**coding**	**theta_C**	**14**	0.01072 (0.00840, 0.01311)	0.03499 (0.01983, 0.05264)	0.01957 (0.00511, 0.03659)	0.03493 (0.01950, 0.05202)
**coding**	**theta_C**	**15**	0.01060 (0.00932, 0.01192)	0.03324 (0.01989, 0.04887)	0.03394 (0.01825, 0.05208)	0.03311 (0.01997, 0.04880)
**coding**	**theta_C**	**16**	0.00188 (0.00072, 0.00308)	0.01599 (0.00380, 0.03141)	0.01388 (0.00233, 0.02905)	0.01663 (0.00412, 0.03220)
**coding**	**theta_C**	**17**	0.00613 (0.00562, 0.00664)	0.01626 (0.01173, 0.02148)	0.03195 (0.01645, 0.05032)	0.01616 (0.01166, 0.02125)
**coding**	**theta_C**	**18**	0.00739 (0.00666, 0.00814)	0.01579 (0.00347, 0.03163)	0.01300 (0.00173, 0.02773)	0.01565 (0.00372, 0.03128)
**coding**	**theta_C**	**19**	0.00763 (0.00699, 0.00828)	0.02235 (0.01546, 0.03059)	0.02683 (0.01549, 0.04090)	0.02234 (0.01536, 0.03049)
**coding**	**theta_C**	**20**	0.00713 (0.00648, 0.00778)	0.03460 (0.02040, 0.05102)	0.03804 (0.02098, 0.05742)	0.03471 (0.02069, 0.05138)
**coding**	**theta_C**	**21**	n/a	n/a	n/a	n/a
**coding**	**theta_M**	**1**	0.00160 (0.00145, 0.00176)	0.00096 (8e-04, 0.00110)	0.00052 (0.00026, 0.00077)	0.00096 (0.00081, 0.00111)
**coding**	**theta_M**	**2**	3e-05 (1e-05, 5e-05)	2e-05 (1e-05, 3e-05)	1e-05 (0.00000, 2e-05)	2e-05 (1e-05, 3e-05)
**coding**	**theta_M**	**3**	0.00155 (0.00120, 0.00190)	0.00088 (0.00059, 0.00117)	0.00021 (2e-05, 4e-04)	0.00091 (0.00061, 0.00120)
**coding**	**theta_M**	**4**	0.00417 (0.00348, 0.00489)	0.00307 (0.00236, 0.00380)	0.00163 (0.00045, 0.00284)	0.00312 (0.00240, 0.00383)
**coding**	**theta_M**	**5**	0.00895 (0.00746, 0.01050)	0.00725 (0.00601, 0.00856)	0.01316 (0.00566, 0.02343)	0.00723 (0.00605, 0.00847)
**coding**	**theta_M**	**6**	0.00108 (0.00098, 0.00118)	0.00043 (0.00032, 0.00054)	9e-05 (2e-05, 0.00018)	0.00044 (0.00033, 0.00055)
**coding**	**theta_M**	**7**	0.00182 (0.00164, 0.00199)	0.00075 (0.00052, 0.00098)	0.00027 (7e-05, 0.00047)	0.00076 (0.00052, 0.00100)
**coding**	**theta_M**	**8**	0.00461 (0.00408, 0.00516)	0.00335 (0.00285, 0.00383)	0.00327 (0.00236, 0.00418)	0.00340 (0.00291, 0.00391)
**coding**	**theta_M**	**9**	4e-05 (1e-05, 6e-05)	2e-05 (1e-05, 3e-05)	1e-05 (0.00000, 2e-05)	2e-05 (1e-05, 4e-05)
**coding**	**theta_M**	**10**	0.00016 (0.00014, 0.00018)	2e-05 (1e-05, 2e-05)	1e-05 (0.00000, 1e-05)	2e-05 (1e-05, 2e-05)
**coding**	**theta_M**	**11**	0.00344 (0.00306, 0.00381)	0.00227 (0.00190, 0.00265)	0.00223 (0.00158, 0.00290)	0.00241 (0.00199, 0.00284)
**coding**	**theta_M**	**12**	0.00123 (0.00109, 0.00136)	0.00058 (0.00046, 0.00071)	0.00024 (9e-05, 0.00038)	0.00059 (0.00046, 0.00071)
**coding**	**theta_M**	**13**	0.00561 (0.00512, 0.00610)	0.00423 (0.00379, 0.00468)	0.00540 (0.00488, 0.00596)	0.00423 (0.00378, 0.00467)
**coding**	**theta_M**	**14**	0.00163 (0.00132, 0.00192)	0.00115 (0.00085, 0.00145)	0.00023 (5e-05, 0.00044)	0.00118 (0.00087, 0.00149)
**coding**	**theta_M**	**15**	0.00387 (0.00347, 0.00425)	0.00240 (0.00194, 0.00286)	0.00192 (0.00137, 0.00248)	0.00242 (0.00196, 0.00287)
**coding**	**theta_M**	**16**	9e-05 (3e-05, 0.00014)	3e-05 (1e-05, 5e-05)	2e-05 (0.00000, 4e-05)	4e-05 (1e-05, 6e-05)
**coding**	**theta_M**	**17**	0.00306 (0.00282, 0.00330)	0.00191 (0.00163, 0.00217)	0.00107 (7e-04, 0.00145)	0.00192 (0.00165, 0.00219)
**coding**	**theta_M**	**18**	9e-05 (5e-05, 0.00012)	1e-05 (0.00000, 2e-05)	0.00000 (0.00000, 1e-05)	1e-05 (1e-05, 2e-05)
**coding**	**theta_M**	**19**	0.00304 (0.00282, 0.00326)	0.00193 (0.00168, 0.00217)	0.00171 (0.00134, 0.00209)	0.00194 (0.00170, 0.00218)
**coding**	**theta_M**	**20**	0.00282 (0.00260, 0.00304)	0.00175 (0.00153, 0.00197)	0.00168 (0.00137, 0.00198)	0.00176 (0.00153, 0.00197)
**coding**	**theta_M**	**21**	n/a	n/a	n/a	n/a
**coding**	**theta_r**	**1**	0.00922 (0.00884, 0.00962)	0.00922 (0.00881, 0.00963)	0.00941 (0.00900, 0.00981)	0.00922 (0.00882, 0.00963)
**coding**	**theta_r**	**2**	0.01300 (0.01235, 0.01363)	0.01335 (0.01269, 0.01404)	0.01338 (0.01270, 0.01404)	0.01335 (0.01267, 0.01402)
**coding**	**theta_r**	**3**	0.01022 (0.00962, 0.01082)	0.01015 (0.00954, 0.01077)	0.01048 (0.00985, 0.01109)	0.01016 (0.00954, 0.01077)
**coding**	**theta_r**	**4**	0.00972 (0.00909, 0.01038)	0.00977 (0.00911, 0.01043)	0.00989 (0.00923, 0.01056)	0.00977 (0.00911, 0.01043)
**coding**	**theta_r**	**5**	0.01038 (0.00976, 0.01097)	0.01021 (0.00961, 0.01081)	0.01040 (0.00979, 0.01100)	0.01022 (0.00962, 0.01083)
**coding**	**theta_r**	**6**	0.00989 (0.00942, 0.01035)	0.01002 (0.00953, 0.01048)	0.01013 (0.00964, 0.01060)	0.01001 (0.00954, 0.01050)
**coding**	**theta_r**	**7**	0.00938 (0.00892, 0.00984)	0.00953 (0.00906, 0.01001)	0.00955 (0.00908, 0.01003)	0.00953 (0.00904, 0.01000)
**coding**	**theta_r**	**8**	0.01014 (0.00961, 0.01069)	0.01008 (0.00954, 0.01062)	0.01022 (0.00966, 0.01077)	0.01008 (0.00954, 0.01062)
**coding**	**theta_r**	**9**	0.01156 (0.01087, 0.01226)	0.01184 (0.01113, 0.01257)	0.01189 (0.01117, 0.01264)	0.01185 (0.01114, 0.01258)
**coding**	**theta_r**	**10**	0.00873 (0.00833, 0.00911)	0.00881 (0.00842, 0.00921)	0.00890 (0.00850, 0.00931)	0.00881 (0.00841, 0.00921)
**coding**	**theta_r**	**11**	0.01052 (0.01006, 0.01099)	0.01052 (0.01004, 0.01099)	0.01057 (0.01010, 0.01104)	0.01052 (0.01005, 0.01101)
**coding**	**theta_r**	**12**	0.01062 (0.01020, 0.01104)	0.01058 (0.01012, 0.01100)	0.01092 (0.01048, 0.01136)	0.01058 (0.01014, 0.01102)
**coding**	**theta_r**	**13**	0.00903 (0.00861, 0.00946)	0.00880 (0.00839, 0.00922)	0.00902 (0.00860, 0.00945)	0.00879 (0.00838, 0.00921)
**coding**	**theta_r**	**14**	0.01081 (0.01020, 0.01144)	0.01084 (0.01019, 0.01146)	0.01102 (0.01040, 0.01167)	0.01084 (0.01020, 0.01147)
**coding**	**theta_r**	**15**	0.01138 (0.01082, 0.01196)	0.01145 (0.01086, 0.01203)	0.01151 (0.01094, 0.01209)	0.01145 (0.01088, 0.01205)
**coding**	**theta_r**	**16**	0.01068 (0.01004, 0.01132)	0.01098 (0.01032, 0.01164)	0.01097 (0.01031, 0.01164)	0.01098 (0.01030, 0.01164)
**coding**	**theta_r**	**17**	0.00895 (0.00853, 0.00935)	0.00883 (0.00839, 0.00924)	0.00909 (0.00868, 0.00951)	0.00882 (0.00840, 0.00923)
**coding**	**theta_r**	**18**	0.00890 (0.00842, 0.00934)	0.00888 (0.00840, 0.00933)	0.00896 (0.00850, 0.00943)	0.00888 (0.00842, 0.00935)
**coding**	**theta_r**	**19**	0.00978 (0.00934, 0.01021)	0.00986 (0.00942, 0.01030)	0.00989 (0.00944, 0.01033)	0.00986 (0.00941, 0.01029)
**coding**	**theta_r**	**20**	0.00882 (0.00843, 0.00921)	0.00885 (0.00844, 0.00924)	0.00896 (0.00856, 0.00936)	0.00885 (0.00845, 0.00925)
**coding**	**theta_r**	**21**	0.00789 (0.00726, 0.00851)	0.00787 (0.00730, 0.00845)	0.00787 (0.00728, 0.00845)	0.00785 (0.00727, 0.00842)
**coding**	**theta_s**	**1**	0.01174 (0.01108, 0.01241)	0.00274 (0.00038, 0.00538)	0.00921 (0.00840, 0.01002)	0.00272 (0.00035, 0.00533)
**coding**	**theta_s**	**2**	0.03018 (0.02809, 0.03230)	0.00808 (0.00286, 0.01307)	0.01574 (0.01326, 0.01816)	0.00819 (0.00286, 0.01307)
**coding**	**theta_s**	**3**	0.01872 (0.01717, 0.02031)	0.00795 (0.00066, 0.01853)	0.01336 (0.01181, 0.01486)	0.00792 (0.00056, 0.01837)
**coding**	**theta_s**	**4**	0.01544 (0.01406, 0.01680)	0.00732 (0.00135, 0.01368)	0.01140 (0.00954, 0.01324)	0.00738 (0.00135, 0.01400)
**coding**	**theta_s**	**5**	0.01616 (0.01490, 0.01742)	0.01232 (0.00128, 0.02663)	0.01534 (0.01350, 0.01719)	0.01222 (0.00126, 0.02633)
**coding**	**theta_s**	**6**	0.01681 (0.01543, 0.01819)	0.00601 (0.00234, 0.00950)	0.01055 (0.00925, 0.01188)	0.00590 (0.00219, 0.00938)
**coding**	**theta_s**	**7**	0.01349 (0.01252, 0.01446)	0.00737 (0.00467, 0.00991)	0.01015 (0.00916, 0.01114)	0.00731 (0.00456, 0.00980)
**coding**	**theta_s**	**8**	0.01527 (0.01394, 0.01656)	0.01154 (0.00123, 0.02495)	0.01361 (0.01140, 0.01588)	0.01157 (0.00097, 0.02508)
**coding**	**theta_s**	**9**	0.02563 (0.02360, 0.02775)	0.00564 (0.00126, 0.00999)	0.01242 (0.01008, 0.01476)	0.00555 (0.00135, 0.00993)
**coding**	**theta_s**	**10**	0.01170 (0.01070, 0.01273)	0.00371 (0.00153, 0.00598)	0.00892 (0.00787, 0.00996)	0.00380 (0.00155, 0.00604)
**coding**	**theta_s**	**11**	0.01317 (0.01229, 0.01407)	0.00738 (0.00083, 0.01548)	0.01251 (0.01132, 0.01367)	0.00738 (0.00097, 0.01594)
**coding**	**theta_s**	**12**	0.01729 (0.01620, 0.01834)	0.00400 (0.00049, 0.00811)	0.01111 (0.00994, 0.01227)	0.00396 (0.00057, 0.00808)
**coding**	**theta_s**	**13**	0.01247 (0.01158, 0.01336)	0.01200 (0.00123, 0.02626)	0.01329 (0.01231, 0.01428)	0.01201 (0.00112, 0.02619)
**coding**	**theta_s**	**14**	0.01920 (0.01784, 0.02059)	0.00808 (0.00094, 0.01660)	0.01443 (0.01295, 0.01595)	0.00785 (0.00095, 0.01605)
**coding**	**theta_s**	**15**	0.01632 (0.01473, 0.01791)	0.00766 (0.00270, 0.01221)	0.01234 (0.01042, 0.01423)	0.00766 (0.00260, 0.01221)
**coding**	**theta_s**	**16**	0.02462 (0.02276, 0.02652)	0.00800 (0.00378, 0.01211)	0.01301 (0.01097, 0.01499)	0.00813 (0.00398, 0.01209)
**coding**	**theta_s**	**17**	0.01290 (0.01190, 0.01390)	0.00647 (0.00035, 0.01602)	0.00993 (0.00881, 0.01106)	0.00652 (0.00048, 0.01614)
**coding**	**theta_s**	**18**	0.00966 (0.00860, 0.01075)	0.00487 (0.00237, 0.00716)	0.00837 (0.00729, 0.00946)	0.00485 (0.00237, 0.00728)
**coding**	**theta_s**	**19**	0.01337 (0.01245, 0.01432)	0.00386 (0.00094, 0.00674)	0.01046 (0.00927, 0.01167)	0.00386 (0.00102, 0.00680)
**coding**	**theta_s**	**20**	0.01267 (0.01177, 0.01356)	0.00410 (0.00093, 0.00743)	0.00993 (0.00865, 0.01120)	0.00403 (0.00078, 0.00742)
**coding**	**theta_s**	**21**	0.00154 (7e-04, 0.00241)	6e-04 (0.00015, 0.00117)	0.00057 (1e-04, 0.00115)	0.00052 (7e-05, 0.00110)
**coding**	**theta_c**	**1**	n/a	0.00592 (0.00553, 0.00632)	0.00438 (0.00324, 0.00548)	0.00577 (0.00528, 0.00624)

**coding**	**theta_c**	**2**	n/a	0.01875 (0.01714, 0.02040)	0.01842 (0.01329, 0.02355)	0.01848 (0.01675, 0.02026)
**coding**	**theta_c**	**3**	n/a	0.00980 (0.00892, 0.01069)	0.00918 (0.00644, 0.01181)	0.00938 (0.00817, 0.01052)
**coding**	**theta_c**	**4**	n/a	0.00984 (0.00871, 0.01100)	0.00687 (0.00452, 0.00919)	0.00925 (0.00750, 0.01087)
**coding**	**theta_c**	**5**	n/a	0.00966 (0.00875, 0.01057)	0.00702 (0.00331, 0.01108)	0.00850 (0.00620, 0.01025)
**coding**	**theta_c**	**6**	n/a	0.00822 (0.00758, 0.00886)	0.00821 (0.00748, 0.00892)	0.00814 (0.00749, 0.00881)
**coding**	**theta_c**	**7**	n/a	0.00739 (0.00673, 0.00805)	0.00660 (0.00569, 0.00752)	0.00728 (0.00658, 0.00794)
**coding**	**theta_c**	**8**	n/a	0.00843 (0.00765, 0.00925)	0.00393 (0.00156, 0.00624)	0.00761 (0.00584, 0.00901)
**coding**	**theta_c**	**9**	n/a	0.01472 (0.01334, 0.01611)	0.01211 (0.00888, 0.01527)	0.01428 (0.01272, 0.01594)
**coding**	**theta_c**	**10**	n/a	0.00908 (0.00859, 0.00956)	0.00837 (0.00739, 0.00929)	0.00899 (0.00847, 0.00952)
**coding**	**theta_c**	**11**	n/a	0.00741 (0.00680, 0.00803)	0.00289 (0.00118, 0.00462)	0.00614 (0.00433, 0.00777)
**coding**	**theta_c**	**12**	n/a	0.00822 (0.00769, 0.00875)	0.00696 (0.00577, 0.00807)	0.00814 (0.00760, 0.00870)
**coding**	**theta_c**	**13**	n/a	0.00604 (0.00559, 0.00649)	0.00034 (4e-05, 0.00081)	0.00596 (0.00549, 0.00644)
**coding**	**theta_c**	**14**	n/a	0.01163 (0.01063, 0.01269)	0.00956 (0.00705, 0.01195)	0.01086 (0.00917, 0.01239)
**coding**	**theta_c**	**15**	n/a	0.00904 (0.00799, 0.01013)	0.00625 (0.00403, 0.00845)	0.00873 (0.00748, 0.00995)
**coding**	**theta_c**	**16**	n/a	0.01325 (0.01201, 0.01442)	0.00953 (0.00656, 0.01240)	0.01271 (0.01113, 0.01423)
**coding**	**theta_c**	**17**	n/a	0.00577 (0.00531, 0.00624)	0.00464 (0.00360, 0.00564)	0.00564 (0.00512, 0.00617)
**coding**	**theta_c**	**18**	n/a	0.00793 (0.00740, 0.00845)	0.00688 (0.00586, 0.00785)	0.00783 (0.00730, 0.00843)
**coding**	**theta_c**	**19**	n/a	0.00701 (0.00646, 0.00755)	0.00505 (0.00390, 0.00622)	0.00692 (0.00637, 0.00751)
**coding**	**theta_c**	**20**	n/a	0.00622 (0.00575, 0.00671)	0.00346 (0.00244, 0.00448)	0.00613 (0.00559, 0.00664)
**coding**	**theta_c**	**21**	n/a	0.01326 (0.00049, 0.02916)	0.00994 (2e-04, 0.02363)	0.01392 (0.00074, 0.02982)
**coding**	**theta_m**	**1**	n/a	0.01186 (0.00764, 0.01684)	0.01052 (0.00716, 0.01443)	0.01285 (0.00774, 0.01890)
**coding**	**theta_m**	**2**	n/a	0.00837 (0.00067, 0.01928)	0.02025 (0.00812, 0.03512)	0.01150 (0.00109, 0.02548)
**coding**	**theta_m**	**3**	n/a	0.02248 (0.01182, 0.03529)	0.02500 (0.01333, 0.03935)	0.02642 (0.01258, 0.04288)
**coding**	**theta_m**	**4**	n/a	0.00955 (0.00512, 0.01472)	0.01189 (0.00931, 0.01474)	0.01224 (0.00532, 0.02211)
**coding**	**theta_m**	**5**	n/a	0.00878 (0.00483, 0.01332)	0.01040 (0.00769, 0.01322)	0.01539 (0.00522, 0.03242)
**coding**	**theta_m**	**6**	n/a	0.00633 (0.00424, 0.00863)	0.00466 (0.00378, 0.00561)	0.00652 (0.00433, 0.00897)
**coding**	**theta_m**	**7**	n/a	0.00688 (0.00477, 0.00916)	0.00748 (0.00573, 0.00938)	0.00711 (0.00486, 0.00957)
**coding**	**theta_m**	**8**	n/a	0.01368 (0.00779, 0.02062)	0.01323 (0.00823, 0.01931)	0.01897 (0.00836, 0.03370)
**coding**	**theta_m**	**9**	n/a	0.01822 (0.00617, 0.03263)	0.01581 (0.00742, 0.02590)	0.02056 (0.00726, 0.03662)
**coding**	**theta_m**	**10**	n/a	0.01701 (0.00390, 0.03312)	0.01254 (0.00381, 0.02342)	0.02008 (0.00570, 0.03780)
**coding**	**theta_m**	**11**	n/a	0.01017 (0.00639, 0.01451)	0.01999 (0.00881, 0.03413)	0.02309 (0.00670, 0.04651)
**coding**	**theta_m**	**12**	n/a	0.01271 (0.00826, 0.01784)	0.01266 (0.00861, 0.01736)	0.01329 (0.00845, 0.01879)
**coding**	**theta_m**	**13**	n/a	0.00798 (0.00574, 0.01038)	0.01082 (0.00196, 0.02381)	0.00826 (0.00590, 0.01084)
**coding**	**theta_m**	**14**	n/a	0.01480 (0.00756, 0.02350)	0.01327 (0.00924, 0.01775)	0.01777 (0.00836, 0.02951)
**coding**	**theta_m**	**15**	n/a	0.01125 (0.00709, 0.01621)	0.01775 (0.01172, 0.02492)	0.01212 (0.00728, 0.01782)
**coding**	**theta_m**	**16**	n/a	0.02264 (0.00868, 0.03850)	0.02669 (0.01434, 0.04141)	0.02575 (0.01028, 0.04349)
**coding**	**theta_m**	**17**	n/a	0.01523 (0.00964, 0.02182)	0.01474 (0.01010, 0.02028)	0.01623 (0.01000, 0.02383)
**coding**	**theta_m**	**18**	n/a	0.01152 (0.00097, 0.02512)	0.00981 (0.00285, 0.01921)	0.01441 (0.00185, 0.03000)
**coding**	**theta_m**	**19**	n/a	0.00760 (0.00563, 0.00969)	0.00927 (0.00716, 0.01153)	0.00776 (0.00577, 0.00998)
**coding**	**theta_m**	**20**	n/a	0.00881 (0.00620, 0.01186)	0.01208 (0.00888, 0.01576)	0.00914 (0.00624, 0.01230)
**coding**	**theta_m**	**21**	n/a	0.01000 (0.00026, 0.02389)	0.01372 (0.00069, 0.02998)	0.01367 (0.00049, 0.02945)
**coding**	**tau_r**	**1**	0.00487 (0.00472, 0.00501)	0.00489 (0.00474, 0.00504)	0.00480 (0.00465, 0.00495)	0.00489 (0.00474, 0.00503)
**coding**	**tau_r**	**2**	0.00644 (0.00620, 0.00667)	0.00638 (0.00614, 0.00661)	0.00636 (0.00613, 0.00660)	0.00638 (0.00613, 0.00660)
**coding**	**tau_r**	**3**	0.00593 (0.00570, 0.00616)	0.00600 (0.00576, 0.00623)	0.00584 (0.00561, 0.00607)	0.00600 (0.00576, 0.00623)
**coding**	**tau_r**	**4**	0.00595 (0.00569, 0.00619)	0.00596 (0.00571, 0.00620)	0.00590 (0.00564, 0.00614)	0.00596 (0.00570, 0.00620)
**coding**	**tau_r**	**5**	0.00579 (0.00557, 0.00602)	0.00589 (0.00567, 0.00611)	0.00579 (0.00556, 0.00601)	0.00589 (0.00567, 0.00611)
**coding**	**tau_r**	**6**	0.00551 (0.00533, 0.00569)	0.00548 (0.00529, 0.00565)	0.00543 (0.00525, 0.00561)	0.00548 (0.00530, 0.00565)
**coding**	**tau_r**	**7**	0.00532 (0.00514, 0.00549)	0.00528 (0.00510, 0.00545)	0.00526 (0.00508, 0.00543)	0.00528 (0.00510, 0.00545)
**coding**	**tau_r**	**8**	0.00549 (0.00528, 0.00569)	0.00554 (0.00534, 0.00574)	0.00546 (0.00525, 0.00566)	0.00554 (0.00533, 0.00573)
**coding**	**tau_r**	**9**	0.00601 (0.00575, 0.00627)	0.00597 (0.00572, 0.00623)	0.00595 (0.00568, 0.00621)	0.00597 (0.00570, 0.00622)
**coding**	**tau_r**	**10**	0.00542 (0.00525, 0.00557)	0.00540 (0.00524, 0.00555)	0.00535 (0.00519, 0.00551)	0.00540 (0.00524, 0.00556)
**coding**	**tau_r**	**11**	0.00494 (0.00478, 0.00511)	0.00496 (0.00479, 0.00512)	0.00492 (0.00475, 0.00509)	0.00496 (0.00478, 0.00512)
**coding**	**tau_r**	**12**	0.00529 (0.00513, 0.00545)	0.00533 (0.00517, 0.00549)	0.00518 (0.00501, 0.00533)	0.00533 (0.00517, 0.00549)
**coding**	**tau_r**	**13**	0.00516 (0.00499, 0.00532)	0.00528 (0.00511, 0.00543)	0.00516 (0.00499, 0.00532)	0.00528 (0.00510, 0.00544)
**coding**	**tau_r**	**14**	0.00654 (0.00630, 0.00677)	0.00656 (0.00632, 0.00679)	0.00647 (0.00624, 0.00671)	0.00655 (0.00631, 0.00678)
**coding**	**tau_r**	**15**	0.00542 (0.00522, 0.00563)	0.00542 (0.00521, 0.00562)	0.00538 (0.00517, 0.00558)	0.00542 (0.00521, 0.00562)
**coding**	**tau_r**	**16**	0.00586 (0.00562, 0.00610)	0.00582 (0.00557, 0.00605)	0.00581 (0.00557, 0.00605)	0.00582 (0.00558, 0.00605)
**coding**	**tau_r**	**17**	0.00507 (0.00492, 0.00523)	0.00514 (0.00497, 0.00529)	0.00502 (0.00486, 0.00518)	0.00514 (0.00497, 0.00529)
**coding**	**tau_r**	**18**	0.00492 (0.00474, 0.00509)	0.00493 (0.00475, 0.00510)	0.00489 (0.00472, 0.00506)	0.00493 (0.00476, 0.00510)
**coding**	**tau_r**	**19**	0.00546 (0.00530, 0.00562)	0.00545 (0.00529, 0.00561)	0.00543 (0.00526, 0.00559)	0.00545 (0.00529, 0.00560)
**coding**	**tau_r**	**20**	0.00503 (0.00488, 0.00519)	0.00504 (0.00489, 0.00519)	0.00499 (0.00484, 0.00514)	0.00504 (0.00489, 0.00519)
**coding**	**tau_r**	**21**	0.00467 (0.00442, 0.00493)	0.00470 (0.00447, 0.00493)	0.00470 (0.00447, 0.00492)	0.00471 (0.00448, 0.00493)
**coding**	**tau_s**	**1**	0.00089 (8e-04, 0.00098)	0.00472 (0.00448, 0.00494)	0.00170 (0.00149, 0.00191)	0.00471 (0.00448, 0.00493)
**coding**	**tau_s**	**2**	6e-05 (2e-05, 1e-04)	0.00559 (0.00486, 0.00629)	0.00284 (0.00227, 0.00339)	0.00556 (0.00479, 0.00629)
**coding**	**tau_s**	**3**	0.00075 (0.00057, 0.00094)	0.00583 (0.00546, 0.00618)	0.00194 (0.00161, 0.00228)	0.00583 (0.00545, 0.00617)
**coding**	**tau_s**	**4**	0.00107 (0.00089, 0.00125)	0.00551 (0.00486, 0.00608)	0.00282 (0.00232, 0.00334)	0.00552 (0.00483, 0.00607)
**coding**	**tau_s**	**5**	0.00112 (0.00095, 0.00128)	0.00580 (0.00550, 0.00607)	0.00209 (0.00156, 0.00263)	0.00580 (0.00551, 0.00607)
**coding**	**tau_s**	**6**	0.00142 (0.00126, 0.00158)	0.00483 (0.00431, 0.00532)	0.00277 (0.00252, 0.00302)	0.00485 (0.00435, 0.00534)
**coding**	**tau_s**	**7**	0.00124 (0.00111, 0.00137)	0.00409 (0.00351, 0.00472)	0.00218 (0.00196, 0.00240)	0.00411 (0.00350, 0.00472)
**coding**	**tau_s**	**8**	0.00144 (0.00127, 0.00160)	0.00541 (0.00508, 0.00570)	0.00223 (0.00160, 0.00281)	0.00541 (0.00508, 0.00571)
**coding**	**tau_s**	**9**	7e-05 (2e-05, 0.00012)	0.00538 (0.00472, 0.00596)	0.00305 (0.00251, 0.00355)	0.00539 (0.00473, 0.00596)
**coding**	**tau_s**	**10**	0.00178 (0.00161, 0.00195)	0.00482 (0.00439, 0.00521)	0.00252 (0.00226, 0.00277)	0.00480 (0.00438, 0.00522)

coding	tau_s	11	0.00109 (0.00097, 0.00121)	0.00472 (0.00433, 0.00506)	0.00133 (0.00109, 0.00160)	0.00474 (0.00436, 0.00507)
coding	tau_s	12	0.00093 (0.00082, 0.00105)	0.00518 (0.00492, 0.00540)	0.00235 (0.00209, 0.00260)	0.00517 (0.00492, 0.00540)
coding	tau_s	13	0.00160 (0.00148, 0.00172)	0.00521 (0.00499, 0.00541)	0.00161 (0.00147, 0.00177)	0.00521 (0.00499, 0.00541)
coding	tau_s	14	0.00081 (0.00065, 0.00098)	0.00628 (0.00575, 0.00671)	0.00232 (0.00193, 0.00270)	0.00627 (0.00571, 0.00671)
coding	tau_s	15	0.00179 (0.00161, 0.00197)	0.00476 (0.00418, 0.00531)	0.00278 (0.00241, 0.00314)	0.00476 (0.00419, 0.00532)
coding	tau_s	16	1e-04 (3e-05, 0.00016)	0.00473 (0.00398, 0.00545)	0.00260 (0.00216, 0.00303)	0.00470 (0.00394, 0.00542)
coding	tau_s	17	0.00168 (0.00155, 0.00181)	0.00500 (0.00473, 0.00525)	0.00239 (0.00217, 0.00260)	0.00500 (0.00473, 0.00525)
coding	tau_s	18	0.00184 (0.00164, 0.00204)	0.00416 (0.00369, 0.00462)	0.00229 (0.00202, 0.00256)	0.00416 (0.00367, 0.00463)
coding	tau_s	19	0.00172 (0.00160, 0.00184)	0.00507 (0.00471, 0.00541)	0.00266 (0.00240, 0.00292)	0.00507 (0.00470, 0.00541)
coding	tau_s	20	0.00150 (0.00139, 0.00162)	0.00476 (0.00445, 0.00504)	0.00239 (0.00209, 0.00267)	0.00476 (0.00445, 0.00504)
coding	tau_s	21	0.00362 (0.00324, 0.00398)	0.00430 (0.00392, 0.00464)	0.00432 (0.00392, 0.00468)	0.00436 (0.00399, 0.00472)
coding	tau_c	1	n/a	0.00052 (0.00042, 0.00061)	0.00021 (8e-05, 0.00033)	0.00053 (0.00043, 0.00062)
coding	tau_c	2	n/a	4e-05 (3e-05, 6e-05)	2e-05 (0.00000, 4e-05)	4e-05 (2e-05, 7e-05)
coding	tau_c	3	n/a	0.00043 (0.00028, 0.00058)	8e-05 (0.00000, 0.00016)	0.00045 (0.00029, 6e-04)
coding	tau_c	4	n/a	8e-04 (0.00058, 0.00101)	0.00022 (4e-05, 0.00042)	0.00083 (6e-04, 0.00106)
coding	tau_c	5	n/a	0.00099 (0.00082, 0.00114)	0.00044 (0.00000, 0.00077)	0.00104 (0.00086, 0.00123)
coding	tau_c	6	n/a	0.00042 (0.00029, 0.00055)	6e-05 (1e-05, 0.00012)	0.00043 (3e-04, 0.00056)
coding	tau_c	7	n/a	4e-04 (0.00025, 0.00056)	1e-04 (2e-05, 0.00019)	0.00041 (0.00025, 0.00058)
coding	tau_c	8	n/a	0.00106 (0.00088, 0.00123)	0.00073 (0.00042, 0.00107)	0.00110 (0.00091, 0.00128)
coding	tau_c	9	n/a	3e-05 (2e-05, 5e-05)	2e-05 (0.00000, 4e-05)	4e-05 (2e-05, 7e-05)
coding	tau_c	10	n/a	5e-05 (2e-05, 7e-05)	1e-05 (0.00000, 3e-05)	4e-05 (3e-05, 6e-05)
coding	tau_c	11	n/a	0.00072 (0.00058, 0.00086)	6e-04 (0.00036, 0.00082)	0.00081 (0.00062, 0.00099)
coding	tau_c	12	n/a	0.00041 (3e-04, 5e-04)	0.00013 (4e-05, 0.00022)	0.00041 (0.00031, 0.00051)
coding	tau_c	13	n/a	0.00115 (0.00103, 0.00128)	0.00147 (0.00131, 0.00160)	0.00116 (0.00103, 0.00128)
coding	tau_c	14	n/a	0.00058 (0.00041, 0.00074)	8e-05 (1e-05, 0.00016)	6e-04 (0.00042, 0.00077)
coding	tau_c	15	n/a	0.00094 (0.00069, 0.00118)	0.00064 (0.00039, 0.00088)	0.00096 (0.00071, 0.00120)
coding	tau_c	16	n/a	3e-05 (1e-05, 6e-05)	2e-05 (0.00000, 4e-05)	4e-05 (2e-05, 7e-05)
coding	tau_c	17	n/a	0.00098 (8e-04, 0.00115)	4e-04 (0.00022, 0.00057)	0.00100 (0.00082, 0.00117)
coding	tau_c	18	n/a	3e-05 (1e-05, 5e-05)	1e-05 (0.00000, 3e-05)	3e-05 (1e-05, 5e-05)
coding	tau_c	19	n/a	0.00091 (0.00075, 0.00106)	6e-04 (4e-04, 8e-04)	0.00091 (0.00075, 0.00106)
coding	tau_c	20	n/a	0.00082 (0.00069, 0.00094)	0.00065 (0.00049, 0.00082)	0.00082 (0.00069, 0.00095)
coding	tau_c	21	n/a	9e-04 (6e-05, 0.00172)	0.00092 (6e-05, 0.00174)	0.00106 (0.00019, 0.00191)
coding	phi_c<-m	1	n/a	n/a	0.13595 (0.07830, 0.19593)	0.00728 (0.00000, 0.01937)
coding	phi_c<-m	2	n/a	n/a	0.10401 (0.00518, 0.20372)	0.00698 (0.00000, 0.02301)
coding	phi_c<-m	3	n/a	n/a	0.09711 (0.00000, 0.19803)	0.01299 (0.00000, 0.03940)
coding	phi_c<-m	4	n/a	n/a	0.32695 (0.23205, 0.42542)	0.02137 (0.00000, 0.06803)
coding	phi_c<-m	5	n/a	n/a	0.31102 (0.15996, 0.46466)	0.04204 (0.00000, 0.11832)
coding	phi_c<-m	6	n/a	n/a	0.02639 (0.01151, 0.04218)	0.00282 (0.00000, 0.00841)
coding	phi_c<-m	7	n/a	n/a	0.07026 (0.03709, 0.10615)	0.00466 (0.00000, 0.01384)
coding	phi_c<-m	8	n/a	n/a	0.24908 (0.12407, 0.38234)	0.03137 (0.00000, 0.09080)
coding	phi_c<-m	9	n/a	n/a	0.15048 (0.06718, 0.24245)	0.01214 (0.00000, 0.03687)
coding	phi_c<-m	10	n/a	n/a	0.04169 (0.00785, 0.07886)	0.00386 (0.00000, 0.01209)
coding	phi_c<-m	11	n/a	n/a	0.13794 (0.00000, 0.27567)	0.05520 (0.00000, 0.12933)
coding	phi_c<-m	12	n/a	n/a	0.10137 (0.05642, 0.15020)	0.00281 (0.00000, 0.00859)
coding	phi_c<-m	13	n/a	n/a	0.19210 (0.00176, 0.35197)	0.00367 (0.00000, 0.01101)
coding	phi_c<-m	14	n/a	n/a	0.18839 (0.10709, 0.27442)	0.02317 (0.00000, 0.06168)
coding	phi_c<-m	15	n/a	n/a	0.15730 (0.07160, 0.24443)	0.01095 (0.00000, 0.03343)
coding	phi_c<-m	16	n/a	n/a	0.15124 (0.04559, 0.25918)	0.01604 (0.00000, 0.04798)
coding	phi_c<-m	17	n/a	n/a	0.07873 (0.03629, 0.12391)	0.00528 (0.00000, 0.01605)
coding	phi_c<-m	18	n/a	n/a	0.05697 (0.01679, 0.10592)	0.00448 (0.00000, 0.01414)
coding	phi_c<-m	19	n/a	n/a	0.11574 (0.07158, 0.16181)	0.00370 (0.00000, 0.01106)
coding	phi_c<-m	20	n/a	n/a	0.17410 (0.11990, 0.23178)	0.00442 (0.00000, 0.01314)
coding	phi_c<-m	21	n/a	n/a	0.32184 (0.03771, 0.73888)	0.49378 (0.01240, 0.96903)
coding	phi_m<-c	1	n/a	0.51186 (0.47796, 0.54512)	n/a	0.50635 (0.47215, 0.54122)
coding	phi_m<-c	2	n/a	0.44214 (0.37537, 0.50479)	n/a	0.43340 (0.36168, 0.50215)
coding	phi_m<-c	3	n/a	0.53862 (0.49402, 0.58446)	n/a	0.53205 (0.48471, 0.57871)
coding	phi_m<-c	4	n/a	0.61804 (0.54779, 0.68624)	n/a	0.60871 (0.53344, 0.68180)
coding	phi_m<-c	5	n/a	0.64934 (0.60737, 0.69024)	n/a	0.63273 (0.58153, 0.68286)
coding	phi_m<-c	6	n/a	0.31478 (0.25730, 0.37125)	n/a	0.31434 (0.25751, 0.37057)
coding	phi_m<-c	7	n/a	0.36471 (0.29479, 0.43596)	n/a	0.36202 (0.29172, 0.43635)
coding	phi_m<-c	8	n/a	0.57075 (0.52290, 0.61786)	n/a	0.55627 (0.50236, 0.61042)
coding	phi_m<-c	9	n/a	0.42973 (0.36364, 0.49511)	n/a	0.42014 (0.35121, 0.48919)
coding	phi_m<-c	10	n/a	0.35381 (0.31389, 0.39544)	n/a	0.34922 (0.30748, 0.38952)
coding	phi_m<-c	11	n/a	0.57335 (0.52111, 0.62507)	n/a	0.55867 (0.49750, 0.61703)
coding	phi_m<-c	12	n/a	0.43229 (0.39754, 0.46689)	n/a	0.43008 (0.39587, 0.46511)
coding	phi_m<-c	13	n/a	0.50966 (0.47918, 0.54042)	n/a	0.50719 (0.47672, 0.53863)
coding	phi_m<-c	14	n/a	0.60629 (0.55528, 0.65595)	n/a	0.58939 (0.53058, 0.64735)
coding	phi_m<-c	15	n/a	0.38112 (0.30490, 0.45615)	n/a	0.37351 (0.29823, 0.45334)
coding	phi_m<-c	16	n/a	0.37992 (0.30787, 0.45231)	n/a	0.36546 (0.28624, 0.44105)
coding	phi_m<-c	17	n/a	0.43462 (0.38634, 0.47966)	n/a	0.43319 (0.38545, 0.47877)
coding	phi_m<-c	18	n/a	0.34645 (0.29424, 0.39884)	n/a	0.34238 (0.28776, 0.39390)
coding	phi_m<-c	19	n/a	0.39945 (0.35239, 0.44644)	n/a	0.39598 (0.34791, 0.44359)

coding	phi_m<-c	20	n/a	0.42318 (0.38219, 0.46390)	n/a	0.41928 (0.37683, 0.46020)
coding	phi_m<-c	21	n/a	0.30713 (0.03269, 0.73111)	n/a	0.50759 (0.04163, 0.99984)

Table S6. Bayes factors for comparing the four models (I, O, B, 0) using coding and noncoding data on each chromosome from Heliconius

chr	Coding loci			Noncoding loci			Coding loci			Noncoding loci		
	ε = 1%	ε = 0.1%	ε = 0.01%	ε = 1%	ε = 0.1%	ε = 0.01%	ε = 1%	ε = 0.1%	ε = 0.01%	ε = 1%	ε = 0.1%	ε = 0.01%
	B_I0						B_O0					
1	∞	∞	∞	∞	∞	∞	∞	∞	∞	∞	∞	∞
2	∞	∞	∞	∞	∞	∞	0.7027	0.9524	1.6667	13.2100	∞	∞
3	∞	∞	∞	∞	∞	∞	0.3013	0.3356	0.2326	∞	∞	∞
4	∞	∞	∞	∞	∞	∞	∞	∞	∞	∞	∞	∞
5	∞	∞	∞	∞	∞	∞	∞	∞	∞	∞	∞	∞
6	∞	∞	∞	∞	∞	∞	1.1865	∞	∞	∞	∞	∞
7	∞	∞	∞	∞	∞	∞	500.0000	∞	∞	∞	∞	∞
8	∞	∞	∞	∞	∞	∞	5.2083	2.5000	2.0000	∞	∞	∞
9	∞	∞	∞	∞	∞	∞	769.2308	∞	∞	∞	∞	∞
10	∞	∞	∞	∞	∞	∞	0.6036	2.2883	2.5000	∞	∞	∞
11	∞	∞	∞	∞	∞	∞	0.3866	0.3191	0.2481	∞	∞	∞
12	∞	∞	∞	∞	∞	∞	∞	∞	∞	∞	∞	∞
13	∞	∞	∞	∞	∞	∞	0.5836	0.7722	0.7463	∞	∞	∞
14	∞	∞	∞	∞	∞	∞	∞	∞	∞	∞	∞	∞
15	∞	∞	∞	∞	∞	∞	∞	∞	∞	∞	∞	∞
16	∞	∞	∞	∞	∞	∞	4.7059	5.7143	∞	∞	∞	∞
17	∞	∞	∞	∞	∞	∞	149.2537	∞	∞	∞	∞	∞
18	∞	∞	∞	∞	∞	∞	2.4728	17.8571	∞	∞	∞	∞
19	∞	∞	∞	∞	∞	∞	∞	∞	∞	∞	∞	∞
20	∞	∞	∞	∞	∞	∞	∞	∞	∞	∞	∞	∞
21	227.2727	∞	∞	∞	∞	∞	250.0000	∞	∞	∞	∞	∞
	B_BI						B_BO					
1	0.0136	0.0090	0.0073	0.0101	0.0025	0.0021	∞	∞	∞	∞	∞	∞
2	0.0129	0.0063	0.0054	0.0116	0.0053	0.0046	∞	∞	∞	∞	∞	∞
3	0.0185	0.0130	0.0140	0.0109	0.0046	0.0043	∞	∞	∞	∞	∞	∞
4	0.0254	0.0196	0.0202	0.0111	0.0049	0.0046	∞	∞	∞	∞	∞	∞
5	0.0410	0.0325	0.0369	0.0122	0.0067	0.0063	∞	∞	∞	∞	∞	∞
6	0.0103	0.0034	0.0028	0.0100	0.0017	0.0011	∞	∞	∞	∞	∞	∞
7	0.0113	0.0051	0.0047	0.0104	0.0036	0.0032	∞	∞	∞	∞	∞	∞
8	0.0357	0.0306	0.0284	0.0125	0.0067	0.0061	∞	∞	∞	∞	∞	∞
9	0.0177	0.0124	0.0133	0.0146	0.0093	0.0086	∞	∞	∞	∞	∞	∞
10	0.0109	0.0041	0.0033	0.0105	0.0038	0.0031	∞	∞	∞	∞	∞	∞
11	0.0406	0.0273	0.0188	0.0107	0.0041	0.0035	∞	∞	∞	∞	∞	∞
12	0.0103	0.0033	0.0028	0.0100	0.0018	0.0012	∞	∞	∞	∞	∞	∞
13	0.0107	0.0042	0.0040	0.0100	0.0019	0.0014	∞	∞	∞	∞	∞	∞
14	0.0313	0.0241	0.0200	0.0118	0.0059	0.0051	∞	∞	∞	∞	∞	∞
15	0.0164	0.0112	0.0104	0.0108	0.0046	0.0043	∞	∞	∞	∞	∞	∞
16	0.0216	0.0151	0.0132	0.0142	0.0086	0.0081	∞	∞	∞	∞	∞	∞
17	0.0117	0.0056	0.0052	0.0100	0.0021	0.0017	∞	∞	∞	∞	∞	∞
18	0.0113	0.0046	0.0041	0.0100	0.0016	0.0010	∞	∞	∞	∞	∞	∞
19	0.0107	0.0042	0.0040	0.0103	0.0039	0.0034	∞	∞	∞	∞	∞	∞
20	0.0111	0.0051	0.0046	0.0102	0.0031	0.0026	∞	∞	∞	∞	∞	∞
21	0.9251	1.0638	0.8333	1.9778	3.7453	4.5455	0.8897	0.8130	1.1111	1.6667	1.8762	1.4925
	B_IO						B_B0					
1	n/a	n/a	n/a	n/a	n/a	n/a	∞	∞	∞	∞	∞	∞
2	n/a	n/a	n/a	n/a	n/a	n/a	∞	∞	∞	∞	∞	∞
3	n/a	n/a	n/a	n/a	n/a	n/a	∞	∞	∞	∞	∞	∞
4	n/a	n/a	n/a	n/a	n/a	n/a	∞	∞	∞	∞	∞	∞
5	n/a	n/a	n/a	n/a	n/a	n/a	∞	∞	∞	∞	∞	∞
6	n/a	n/a	n/a	n/a	n/a	n/a	∞	∞	∞	∞	∞	∞
7	n/a	n/a	n/a	n/a	n/a	n/a	∞	∞	∞	∞	∞	∞
8	n/a	n/a	n/a	n/a	n/a	n/a	∞	∞	∞	∞	∞	∞
9	n/a	n/a	n/a	n/a	n/a	n/a	∞	∞	∞	∞	∞	∞
10	n/a	n/a	n/a	n/a	n/a	n/a	∞	∞	∞	∞	∞	∞
11	n/a	n/a	n/a	n/a	n/a	n/a	∞	∞	∞	∞	∞	∞
12	n/a	n/a	n/a	n/a	n/a	n/a	∞	∞	∞	∞	∞	∞
13	n/a	n/a	n/a	n/a	n/a	n/a	∞	∞	∞	∞	∞	∞
14	n/a	n/a	n/a	n/a	n/a	n/a	∞	∞	∞	∞	∞	∞
15	n/a	n/a	n/a	n/a	n/a	n/a	∞	∞	∞	∞	∞	∞
16	n/a	n/a	n/a	n/a	n/a	n/a	∞	∞	∞	∞	∞	∞
17	n/a	n/a	n/a	n/a	n/a	n/a	∞	∞	∞	∞	∞	∞
18	n/a	n/a	n/a	n/a	n/a	n/a	∞	∞	∞	∞	∞	∞
19	n/a	n/a	n/a	n/a	n/a	n/a	∞	∞	∞	∞	∞	∞
20	n/a	n/a	n/a	n/a	n/a	n/a	∞	∞	∞	∞	∞	∞
21	n/a	n/a	n/a	n/a	n/a	n/a	1.8147	1.8768	1.9444	3.6445	5.6215	6.0380
